# Global burden, risk factors, and temporal trends of ureteral cancer: a comprehensive analysis of cancer registries

**DOI:** 10.1186/s12916-024-03485-x

**Published:** 2024-06-24

**Authors:** Junjie Huang, Wing Sze Pang, Yat Ching Fung, Fung Yu Mak, Sze Chai Chan, Xianjing Liu, Lin Zhang, Don Eliseo Lucero-Prisno, Wanghong Xu, Zhi-Jie Zheng, Marco Moschini, Benjamin Pradere, Francesco Soria, Dmitry Enikeev, Morgan Roupret, Shahrokh Shariat, Anthony Chi-Fai Ng, Jeremy Yuen-Chun Teoh, Martin C. S. Wong

**Affiliations:** 1 Jockey Club School of Public Health and Primary Care, Faculty of Medicine, The Chinese University of Hong Kong, Hong Kong SAR, China; 2 Centre for Health Education and Health Promotion, Faculty of Medicine, The Chinese University of Hong Kong, Hong Kong SAR, China; 3 Department of Radiology and Nuclear Medicine, Erasmus MC University Medical Center, Rotterdam, the Netherlands; 4 Suzhou Industrial Park Monash Research Institute of Science and Technology, Suzhou, China; 5 The School of Public Health and Preventive Medicine, Monash University, Melbourne, VIC Australia; 6 Department of Global Health and Development, London School of Hygiene and Tropical Medicine, London, UK; 7 Faculty of Public Health, Mahidol University, Bangkok, Thailand; 8 School of Public Health, Fudan University, Shanghai, China; 9 Department of Global Health, School of Public Health, Peking University, Beijing, China; 10 Division of Oncology/Unit of Urology, Urological Research Institute, IRCCS Ospedale San Raffaele, Milan, Italy; 11 European Association of Urology – Young Academic Urologists (EAU-YAU), Arnhem, the Netherlands; 12 Department of Urology, La Croix du Sud Hôpital, Quint Fonsegrives, France; 13 Division of Urology, Department of Surgical Sciences, AOU Città Della Salute E Della Scienza Di Torino, Torino School of Medicine, Turin, Italy; 14 Department of Urology, Medical University of Vienna, Vienna, Austria; 15 Institute for Urology and Reproductive Health, Sechenov University, Moscow, Russia; 16 16GRC 5 Predictive Onco-Uro, Sorbonne University, AP-HP, Urology, Pitie-Salpetriere Hospital, Paris, France; 17 Departments of Urology, Weill Cornell Medical College, New York, NY USA; 18 Department of Urology, University of Texas Southwestern, Dallas, TX USA; 19 Department of Urology, Second Faculty of Medicine, Charles University, Prague, Czech Republic; 20 Division of Urology, Department of Special Surgery, Jordan University Hospital, The University of Jordan, Amman, Jordan; 21 Department of Surgery, S.H. Ho Urology Centre, The Chinese University of Hong Kong, Hong Kong SAR, China

**Keywords:** Ureteral cancer, Incidence, Risk factors, Temporal trends

## Abstract

**Background:**

Ureteral cancer is a rare cancer. This study aimed to provide an up-to-date and comprehensive analysis on the global trends of ureteral cancer incidence and its association with lifestyle and metabolic risk factors.

**Methods:**

The incidence of ureteral cancer was estimated from the Cancer Incidence in Five Continents Plus and Global Cancer Observatory databases. We analyzed the (1) global incidence of ureteral cancer by region, country, sex, and age group by age-standardized rates (ASR); (2) associated risk factors on a population level by univariable linear regression with logarithm transformation; and (3) incidence trend of ureteral cancer by sex and age group in different countries by Average Annual Percentage Change (AAPC).

**Results:**

The global age-standardized rate of ureteral cancer incidence in 2022 was 22.3 per 10,000,000 people. Regions with higher human development index (HDI), such as Europe, Northern America, and East Asia, were found to have a higher incidence of ureteral cancer. Higher HDI and gross domestic product (GDP) and a higher prevalence of smoking, alcohol drinking, physical inactivity, unhealthy dietary, obesity, hypertension, diabetes, and lipid disorder were associated with higher incidence of ureteral cancer. An overall increasing trend of ureteral cancer incidence was observed for the past decade, especially among the female population.

**Conclusions:**

Although ureteral cancer was relatively rare, the number of cases reported was rising over the world. The rising trends among females were more evident compared with the other subgroups, especially in European countries. Further studies could be conducted to examine the reasons behind these epidemiological changes and confirm the relationship with the risk factors identified.

**Supplementary Information:**

The online version contains supplementary material available at 10.1186/s12916-024-03485-x.

## Background

Ureteral cancer is an uncommon disease. The incidence rate increased from 0.69 to 0.91 per 100,000 over the past 30 years in the US [1]. Ureteral cancer is closely connected with bladder cancer, and they could occur synchronously or metachronously [2]. Ureteral cancer is a disease of the elderly. It was found that the mean age at the time of diagnosis increased from 68 to 73 years old in past 30 years [1]. Other risk factors include male gender [1], smoking [3, 4], chemical exposure [5], and some region-specific factors, such as the consumption of aristolochic acid in Eastern Asia [3].

Urothelial upper tract carcinoma is a rare disease, and hence the number of epidemiological studies on this disease entity is relatively limited. Some of the previous studies were also limited to a specific country or region [6, 7]. This study aims to fill the research gap by conducting a more comprehensive analysis from a global perspective. We will examine the (1) most recent global incidence of ureteral cancer by region, country, sex, and age group, (2) risk factors associated with ureteral cancer on a population level, and (3) incidence trend of ureteral cancer by sex and age group in different countries.

## Methods

### Data sources

The incidence of ureteral cancer in 185 countries in 2022 was estimated from the Global Cancer Observatory (GLOBOCAN) database [8]. To aid in cancer prevention and research, the GLOBOCAN is an online database that offers statistics on 26 different forms of cancer worldwide, broken down by sex and age group. The International Association of Cancer Registries created this database through partnerships with population-based cancer registries and the World Health Organization, or it is based on publicly available web data. The information was used to estimate incidence-to-mortality ratios, predict trends, and approximate neighboring countries using data from international or national cancer registries [9]. The 10-year cancer incidence was extracted from the Cancer Incidence in Five Continents Plus (CI5 Plus) database in order to determine the site-specific proportions of ureteral cancer incidence [10]. The CI5 Plus database, which contains annual cancer incidence from over 100 cancer registries by various population groups and includes both the most recent and older data available, was used for time trend analyses and to interpret changes over time. We used the proportional estimates (ureteral cancer incidence out of all cancers in urinary system) from CI5 database to estimate the incidence of ureteral cancer from the GLOBOCAN. The Global Burden of Disease (GBD) database was used to extract country-specific data for risk factor analysis, including the prevalence of smoking, alcohol consumption, unhealthy eating, physical inactivity, obesity, hypertension, diabetes, and lipid disorders [11]. The GBD database measures the health burden of diseases, injuries, and risk factors with the goal of enhancing healthcare systems and eliminating disparities. Seven thousand researchers in 156 countries and regions have gathered and examined data on early mortality and disability brought on by more than 350 illnesses and injuries. The United Nations (UN) and the World Bank, respectively, provided each country’s human development index (HDI) and gross domestic product (GDP) per capita data [12, 13].

### Statistical analysis

Univariable linear regression analysis by sex and age was used to analyze the relationships between ureteral cancer incidence and the dependent variables were HDI, GDP per capita, prevalence of smoking, alcohol consumption, unhealthy eating, physical inactivity, obesity, hypertension, diabetes, and lipid disorders for each nation. The Stata 16.0 was used to estimate the linear regression models. The log transformation of the ASR was used as dependent variable to reduce heteroscedasticity of the association of risk factors and incidence of ureteral cancer. The regression outcomes included beta coefficients (*β*) and the matching 95% confidence intervals (CI). The beta coefficient can be interpreted as the change in log(incidence) (ASR) associated with 1% increase of a certain risk factor. All confidence intervals (CI) are presented at the 95% level, and a *p* value of less than 0.05 was used to determine statistical significance.

Trend analysis was carried out using the SEER Program’s (National Cancer Institute of the United States) joinpoint regression analysis software. We performed the joinpoint regression by adopting the recommendations from the analysis guidelines. Firstly, we utilized the Grid Search method to determine the number of joinpoints needed to fit the model. The maximum number of joinpoints is determined based on the number of data points available. In our analysis, which consisted of 7–11 data points, the guideline recommended a maximum of one joinpoint, and we followed this recommendation [14]. Visual inspection and specific intervention were performed on results of joinpoint regression for each population. Outliers were removed when the extreme data occurred at either end of the time period. Piecewise joinpoint regression was conducted when the outliner appeared in the middle of the period. Secondly, we employed the weighted Bayesian information criterion (BIC) model selection method to determine the best model that fits the data, whether it includes a joinpoint or not. The BIC approach identifies the model with the best fit by penalizing the inclusion of extra parameters. According to the guideline, using the weighted BIC model selection method allows for the sensitive detection of joinpoints and provides flexibility in various scenarios [15]. The Average Annual Percentage Change was used to calculate the temporal trend of ureteral cancer incidence [15]. According to standard practice, data from the most recent 10-year period were used in study on cancer epidemiology. The incidence data had been transformed logarithmically, and the associated standard errors had been computed. Then, for various demographic groups, they were used to calculate the AAPC and the 95% CI. The AAPC shows the temporal trends of ureteral cancer; a positive AAPC implies a rising trend, and vice versa. The 95% confidence interval (CI) was employed as a tool to assess the accuracy of trend estimations; for instance, an interval that overlaps with 0 suggests a steady trend without a discernible increase or drop. Additionally, trends in the incidence of ureteral cancer in various demographic groups were investigated by age groups (all population: 0–85 + , younger population: 15–49, older population: 50–74), sexes (male and female), and geographic areas (Asia, Oceania, America, Europe, and Africa). We used 50 as the cut-off to define younger and older populations as it was considered the cut-off for early-onset cancer in the previous literature [16].

## Results

### Global ureteral cancer incidence in 2022

In 2022, the global number of ureteral cancer cases reported was 23,353, with an ASR of 22.3 per 10,000,000 persons (Supplementary Table 1). The greatest ASR of regions was located in Northern Europe (49.2), Eastern Asia (42.6), and Southern Europe (36.2); meanwhile, the lowest ASR was located in Sub-Saharan Africa (1.5), South-Central Asia (2.6), and South America (5.2). In terms of countries, the highest ASR were found in Japan (85.8), UK (56.2), and Republic of Korea (51.2). The lowest ASR of countries were found in Sierra Leone (0.05), the Republic of Congo, (0.50), and Guinea (0.50).

**Fig. 1:**
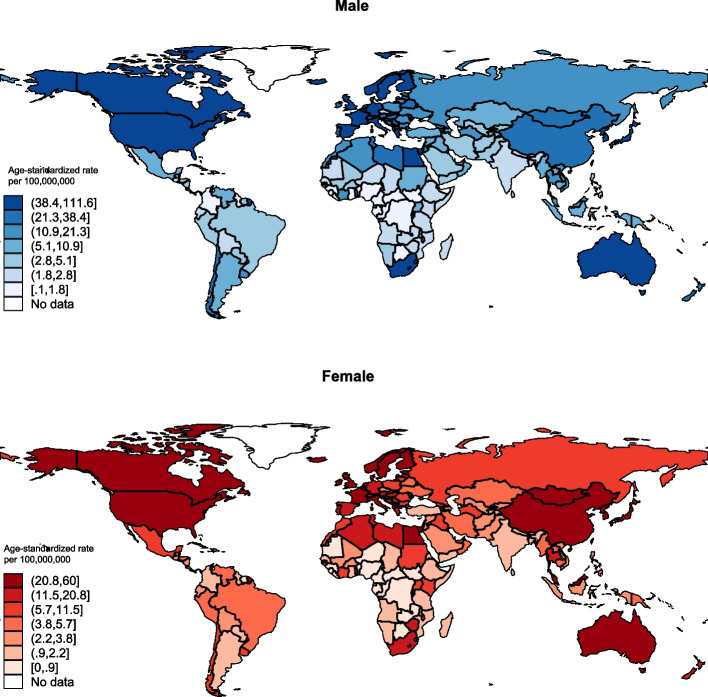
Global incidence of ureteral cancer by sex, all ages, in 2022

Globally, the ASR incidence of ureteral cancer was 28.3 (13,600 cases) per 10,000,000 persons in the male population (Fig. 1), which was higher than that of the females (ASR: 17.2 per 10,000,000 persons; 9753). In the male population, the highest incidence was found in Northern Europe (67.4), followed by Southern Europe (54.3) and Eastern Asia (50.3); in the female population, Eastern Asia (35.5), Northern Europe (32.2), and Western Europe (22.8) have had the highest incidence.

**Fig. 2:**
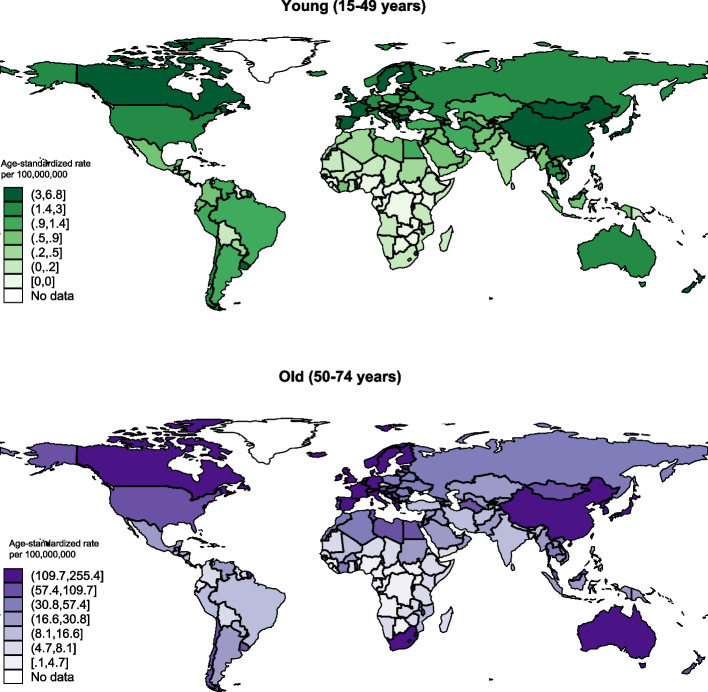
Global incidence of ureteral cancer by age, both sexes, in 2022

In terms of age groups, the incidence of ureteral cancer in the older population aged 50–74 (age truncated incidence rate = 75.2 per 10,000,000 persons; 12,756 cases) was much higher than the younger population aged 15–49 (age truncated incidence rate = 1.6 per 10,000,000 persons; 672 cases) (Fig. 2). For the different regions, the highest ASR incidence in the older population was reported in Eastern Europe (age truncated incidence rate = 160.2 vs. 4.7 in young per 10,000,000 persons), Eastern Asia (age truncated incidence rate = 139.5 vs. 3.57 in young per 10,000,000 persons), and Southern Europe (age truncated incidence rate = 136.6 vs. 3.17 in young per 10,000,000 persons).

### Associations of risk factors with ureteral *cancer* incidence

Overall, the following risk factors were associated with higher ureteral cancer incidence: higher HDI (*β* = 0.501, CI: 0.398 to 0.605, *p* < 0.001), GDP per capita (*β* = 0.315, CI: 0.230 to 0.401, *p* < 0.001), higher prevalence of smoking (*β* = 0.177, CI: 0.154 to 0.200, *p* < 0.001), alcohol drinking (*β* = 0.140, CI: 0.108 to 0.171, *p* < 0.001), unhealthy dietary (*β* = 0.024, CI: 0.006 to 0.041, *p* = 0.008), physical inactivity (*β* = 0.124, CI: 0.064 to 0.184, *p* < 0.001), obesity (*β* = 0.041, CI: 0.024 to 0.058, *p* < 0.001), hypertension (*β* = 0.056, CI: 0.035 to 0.077, *p* < 0.001), diabetes (*β* = 0.094, CI: 0.061 to 0.128, *p* < 0.001), and lipid disorder (*β* = 0.079, CI: 0.067 to 0.091, *p* < 0.001) (Supplementary Table 2).

### Associations of risk factors with ureteral *cancer* incidence by subgroup

**Fig. 3:**
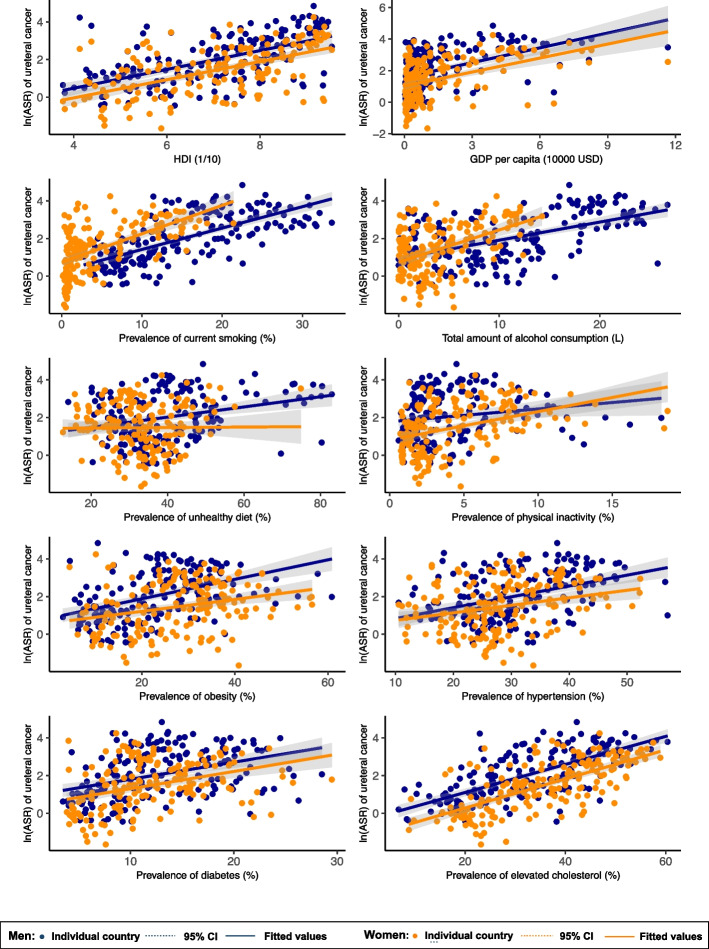
Associations between risk factors and ureteral cancer by sex

**Fig. 4:**
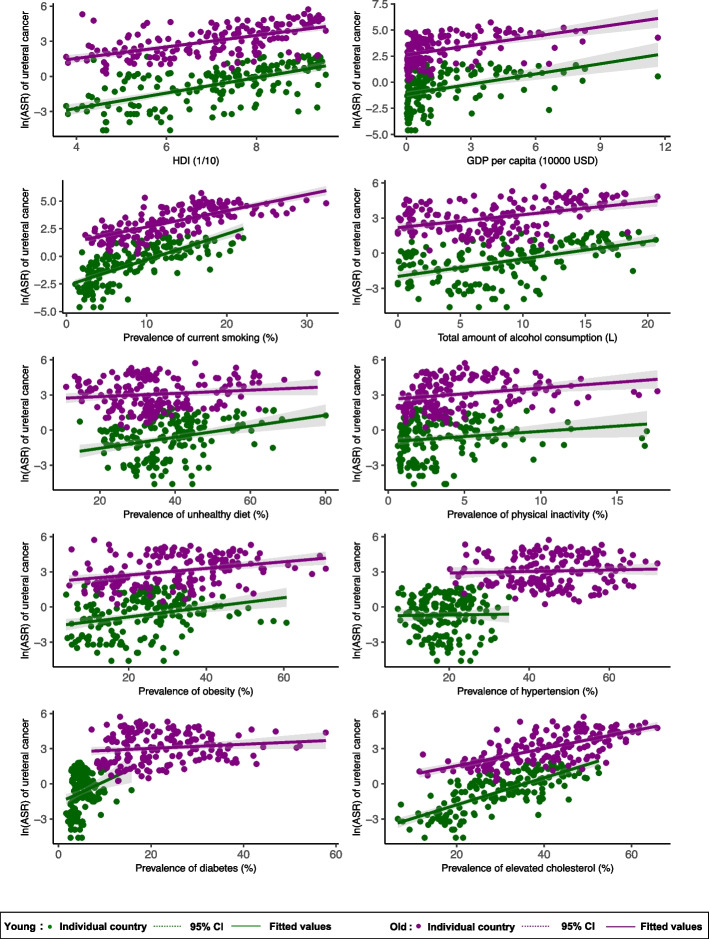
Associations between risk factors and ureteral cancer by age

In the male population (Fig. 3, Supplementary Table 2), higher ureteral cancer incidence was associated with higher HDI (*β* = 0.500, CI: 0.397 to 0.603, *p* < 0.001), GDP per capita (*β* = 0.312, CI: 0.228 to 0.397, *p* < 0.001), higher prevalence of smoking (*β* = 0.114, CI: 0.095 to 0.132, *p* < 0.001), alcohol drinking (*β* = 0.096, CI: 0.074 to 0.119, *p* < 0.001), unhealthy dietary (*β* = 0.027, CI: 0.014 to 0.040, *p* < 0.001), physical inactivity (*β* = 0.068, CI: 0.007 to 0.129, *p* = 0.030), obesity (*β* = 0.052, CI: 0.036 to 0.068, *p* < 0.001), hypertension (*β* = 0.058, CI: 0.038 to 0.077, *p* < 0.001), diabetes (*β* = 0.091, CI: 0.059 to 0.122, *p* < 0.001), and lipid disorder (*β* = 0.075, CI: 0.063 to 0.086, *p* < 0.001). In the female population, higher ureteral cancer incidence was associated with higher HDI (*β* = 0.483, CI: 0.379 to 0.588, *p* < 0.001), GDP per capita (*β* = 0.295, CI: 0.208 to 0.381, *p* < 0.001), higher prevalence of smoking (*β* = 0.151, CI: 0.120 to 0.182, *p* < 0.001), alcohol drinking (*β* = 0.171, CI: 0.125 to 0.218, *p* < 0.001), physical inactivity (*β* = 0.150, CI: 0.096 to 0.204, *p* < 0.001), obesity (*β* = 0.032, CI: 0.015 to 0.048, *p* < 0.001), hypertension (*β* = 0.040, CI: 0.018 to 0.062, *p* < 0.001), diabetes (*β* = 0.092, CI: 0.057 to 0.127, *p* < 0.001), and lipid disorder (*β* = 0.077, CI: 0.065 to 0.090, *p* < 0.001). For the younger population aged 15–49 (Fig. 4), ureteral cancer incidence was associated with higher HDI (*β* = 0.655, CI: 0.530 to 0.780, *p* < 0.001), GDP per capita (*β* = 0.325, CI: 0.214 to 0.436, *p* < 0.001), prevalence of smoking (*β* = 0.233, CI: 0.200 to 0.267, *p* < 0.001), alcohol drinking (*β* = 0.150, CI: 0.111 to 0.189, *p* < 0.001), unhealthy dietary (*β* = 0.047, CI: 0.026 to 0.068, *p* < 0.001), physical inactivity (*β* = 0.089, CI: 0.009 to 0.169, *p* = 0.029), diabetes (*β* = 0.183, CI: 0.075 to 0.292, *p* = 0.001), and lipid disorder (*β* = 0.115, CI: 0.099 to 0.131, *p* < 0.001). For the older population aged 50–74, ureteral cancer incidence was associated with higher HDI (*β* = 0.490, CI: 0.388 to 0.592, *p* < 0.001), GDP per capita (*β* = 0.299, CI: 0.215 to 0.383, *p* < 0.001), prevalence of smoking (*β* = 0.147, CI: 0.127 to 0.167, *p* < 0.001), alcohol drinking (*β* = 0.110, CI: 0.076 to 0.145, *p* < 0.001), unhealthy dietary (*β* = 0.014, CI: − 0.001 to 0.029, *p* = 0.074), physical inactivity (*β* = 0.098, CI: 0.041 to 0.155, *p* = 0.001), obesity (*β* = 0.028, CI: 0.015 to 0.042, *p* < 0.001), and lipid disorder (*β* = 0.074, CI: 0.062 to 0.086, *p* < 0.001).

### Trend analysis of ureteral *cancer* incidence

**Fig. 5:**
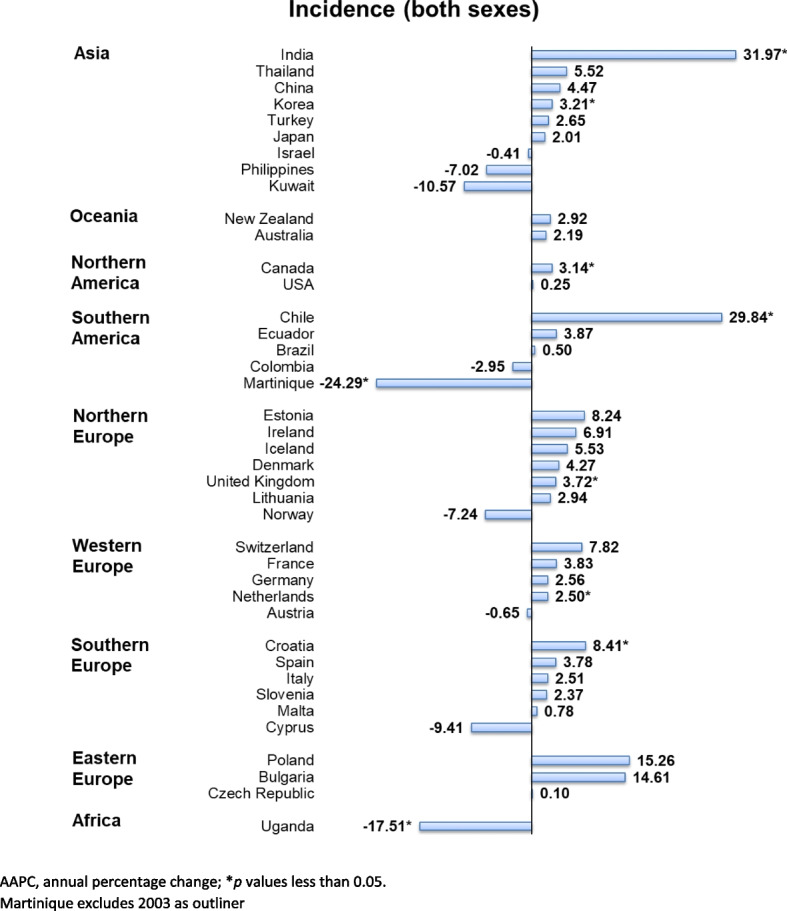
AAPC of the incidence of ureter cancer of both sexes, all ages

Overall, there was an increasing trend in ureteral cancer; 7 countries showed increasing trends and 2 countries showed decreasing trends (Fig. 5). The increasing trends were found in India (AAPC: 31.97, 95% CI 10.33 to 57.85, *p* = 0.002), Chile (AAPC: 29.84, 95% CI 25.74 to 34.06, *p* < 0.001), Croatia (AAPC: 8.41, 95% CI 5.42 to 11.49, *p* < 0.001), UK (AAPC: 3.72, 95% CI 2.60 to 4.86, *p* < 0.001), Korea (AAPC: 3.21, 95% CI 0.71 to 5.77, *p* = 0.018), Canada (AAPC: 3.14, 95% CI 1.07 to 5.25, *p* = 0.008), and Netherlands (AAPC: 2.50, 95% CI 0.25 to 4.81, *p* = 0.034). Meanwhile, the decreasing trends were found in Uganda (AAPC: − 17.51, 95% CI − 18.53 to − 16.48, *p* < 0.001) and Martinique (AAPC: − 24.29, 95% CI − 26.59 to − 21.91, *p* < 0.001) (Supplementary Table 3).

**Fig. 6:**
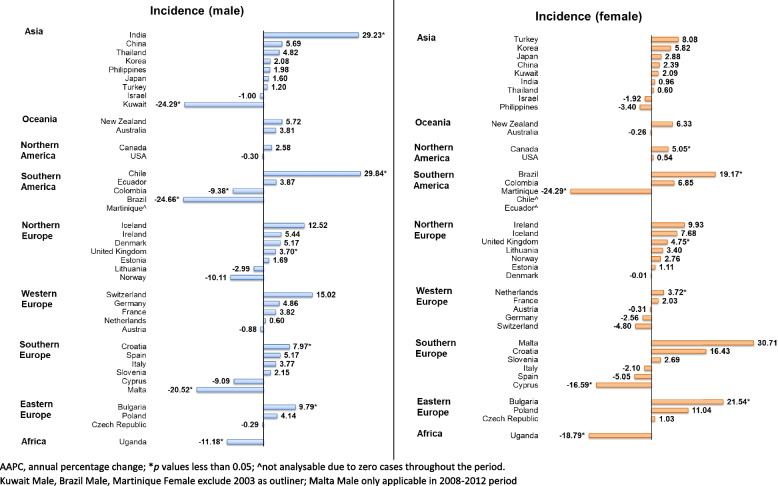
AAPC of ureteral cancer incidence by sex, all ages

In the male population, there was a slight increase in incidence trend, in which 5 country trends were rising and 5 countries were declining (Fig. 6). An obvious rising trend was found in Chile (AAPC: 29.84, 95% CI: 25.74 to 34.06, *p* < 0.001), followed by India (AAPC: 29.23, 95% CI: 12.37 to 48.62, *p* < 0.001), Bulgaria (AAPC: 9.79, 95% CI: 0.98 to 19.36, *p* = 0.033), Croatia (AAPC: 7.97, 95% CI: 3.70 to 12.42, *p* = 0.002), and UK (AAPC: 3.70, 95% CI: 2.28 to 5.14, *p* < 0.001). Brazil (AAPC: − 24.66, 95% CI: − 26.56 to − 22.72, *p* < 0.001), Kuwait (AAPC: − 24.29, 95% CI: − 26.59 to − 21.91, *p* < 0.001), Malta (AAPC: − 20.52, 95% CI: − 35.28 to − 2.40, *p* = 0.038), Uganda (AAPC: − 11.18, 95% CI: − 11.79 to − 10.57, *p* < 0.001), and Colombia (AAPC: − 9.38, 95% CI: − 9.57 to − 9.19, *p* < 0.001) showed declining trends. Meanwhile, there was a rising trend in the female population. The significant rises were presented in 6 countries, including Malta (AAPC: 30.71, 95% CI: 27.08 to 34.45, *p* < 0.001), Bulgaria (AAPC: 21.54, 95% CI: 0.15 to 47.50, *p* = 0.048), Brazil (AAPC: 19.17, 95% CI: 13.59 to 25.03, *p* < 0.001), Canada (AAPC: 5.05, 95% CI: 0.89 to 9.38, *p* = 0.023), UK (AAPC: 4.75, 95% CI: 0.85 to 8.81, *p* = 0.017), and Netherlands (AAPC: 3.72, 95% CI: 0.55 to 6.98, *p* = 0.026). Only 3 countries presented the declining trends: Uganda (AAPC: − 18.79, 95% CI: − 21.95 to − 15.50, *p* < 0.001), Cyprus (AAPC: − 16.59, 95% CI: − 18.84 to − 14.28, *p* < 0.001), and Martinique (AAPC: − 24.29, 95% CI: − 26.59 to − 21.71, *p* < 0.001).

**Fig. 7:**
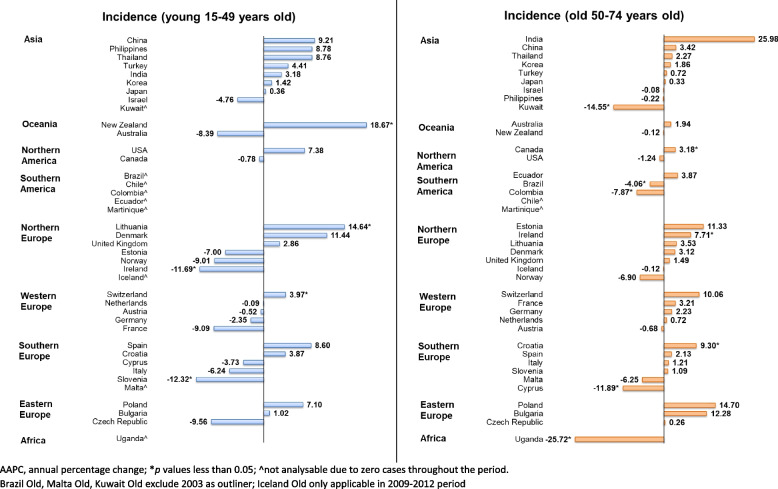
AAPC of ureteral cancer incidence by ages, both sexes

Regarding age group, both the older and younger population presented mixed trends (Fig. 7). In the older population, the increasing trends were found in Croatia (AAPC: 9.30, 95% CI: 5.63 to 13.10, *p* < 0.001), Ireland (AAPC: 7.71, 95% CI: 1.63 to 14.14, *p* = 0.018), and Canada (AAPC: 3.18, 95% CI: 1.07 to 5.34, *p* = 0.008) while the decreasing trends were found in Uganda (AAPC: − 25.72, 95% CI: − 27.73 to − 23.66, *p* < 0.001), Cyprus (AAPC: − 11.89, 95% CI: − 19.56 to − 3.48, *p* = 0.013), Colombia (AAPC: − 7.87, 95% CI: − 11.56 to − 4.02, *p* = 0.002), and Brazil (AAPC: − 4.06, 95% CI: − 4.18 to − 3.93, *p* < 0.001). In the younger population, 3 countries including New Zealand (AAPC: 18.67, 95% CI: 0.98 to 39.45, *p* = 0.038), Lithuania (AAPC: 14.64, 95% CI: 1.77 to 29.14, *p* = 0.024), and Switzerland (AAPC: 3.97, 95% CI: 3.63 to 4.31, *p* < 0.001) reported the rising trends. Meanwhile, Slovenia (AAPC: − 12.32, 95% CI: − 16.12 to − 8.35, *p* < 0.001) and Ireland (AAPC: − 11.69, 95% CI: − 18.93 to − 3.79, *p* = 0.004) presented the declining trends.

## Discussion

### Summary of major findings

This study examines the prevalence, risk factors, and trends of ureteral cancer on a global scale. First, there is a significant regional variation in the prevalence of ureteral cancer; a higher incidence was found in more developed regions such as Eastern Asia and Northern Europe. Second, significant associations were observed between ureteral cancer and several risk factors, including a higher HDI and GDP per capita, higher prevalence of smoking, alcohol drinking, obesity, and lipid disorder. Third, a mixed trend of ureteral cancer incidence was observed globally. However, a remarkable rising trend was found in the female population.

### Variation in the disease burden

The prevalence of ureteral cancer varied significantly by region in 2022. Eastern Asia, Northern Europe, Western Europe, Southern Europe, and Northern America were found to have the greatest disease burden. While the use of aristolochic acid has most likely contributed to the higher incidence of ureteral cancer in Eastern Asia, particularly in Taiwan [17], the higher prevalence of cigarette smoking in the 1990s might have explained the higher incidence in Europe and Northern America [18]. Male seems to be more susceptible to ureteral cancer; men were two times more likely than women suffering from upper tract urothelial carcinoma, as suggested by prior studies [3, 19]. The gender difference may be caused by other risk factors, such as smoking and occupational exposure [20, 21]. Meanwhile, the higher incidence found among the older population was likely a result of the longer exposure to carcinogens including aristolochic acid and cigarette smoking.

### Factors associated with ureteral cancer incidence

Smoking was proven to be one of the factors associated with ureteral cancer incidence in the current study. Such a result aligned with a previous German study (OR = 6.20, 95% CI = 2.04–18.81) [22]. Previous research suggested that the level of aromatic amines in urine was associated with the level of tobacco consumption [23]. Aromatic amines are carcinogenic chemicals which were proven to increase the risk of bladder cancer [24–26]. As the ureter is also exposed to carcinogens in the urine, it is possible that aromatic amine may explain the positive association between smoking and ureteral cancer.

Alcohol drinking was also an associated risk factor of ureteral cancer with an OR of 1.23 (95% CI, 1.08–1.40; *p*= 0.001) in a previous study [27]. It might due to the existence of acetaldehyde, an intermediate carcinogenic substance during the catalyzation of alcohol, in human urine [28, 29]. Notably, a research discovered that the enzyme ALDH2*2, a catalytically inactive subunit, was more prevalent in East Asians than in Caucasians and Africans [30, 31]. When alcohol was consumed and ALDH2*2 disfunction to catalyze, acetaldehyde accumulates rapidly and might cause the carcinogenic effect. Due to the higher prevalence of ADLH2*2 in East Asians, it was possible that the level of acetaldehyde was higher in uterus when compared with other races, leaded to a higher disease burden.

In some specific regions, Chinese herb nephropathy and Balkan endemic nephropathy were also related to ureteral cancer. The use of aristolochic acid–containing plants was popular in the Balkans [32]. Aristolochic acid has been linked to the development of Chinese herb nephropathy and renal failure; it is possible that aristolochic acid might be carcinogenic in individuals who have previously been diagnosed with Chinese herb nephropathy [33]. The regional use of aristolochic acid–containing plants during diet shall explain the higher disease burden in East Asia and Southern Europe.

On the population level, we identified hypertension as a risk factor of ureteral cancer, which was in line with a previous study (OR = 1.3, 95% CI = 1.0–1.8) [34]. Notably risk factors such as higher HDI [35], GDP [36], unhealthy dietary [35], physical inactivity [37], obesity [38], diabetes [39], and lipid diorders [40] were also risk factors of bladder cancers, which further strengthen the association between bladder cancer and ureteral cancer [2]. However, the underlying mechanism remains uncertain and further studies are needed.

### Trends in incidence

The overall trend of ureteral cancer was increasing; the significant rises were found in countries in different regions. The expanding global use of herbal medicine could be the reason. A growing public interest and acceptance of natural therapies has been observed recently in both developed and developing nations [41]. In developing countries, herbal medicine is commonly employed as their primary health care for rural populations [42]. Also, there is a traditional and widespread use of herbal products in developed countries, such as the UK [43] and European countries [44]. Although aristolochic acid has been prohibited in many countries, it is still easily accessible and available on the internet [45, 46]. In addition, misidentification of medicinal plants and contamination with impurities may cause mistaken intake of poison substances [41, 47].

Female was reported to have the most significant rising trend, while the greatest rise was generally found in European countries. This was in line with a previous study which found a 30-fold increase of pelvis and ureteral cancer in Denmark (ASR_1944-1948_: 0.4; ASR_1999-2003_: 12.5) [6]. Although a significant rise was not reported in Denmark based on our study, this may suggest some geographical characteristics shared by European countries as the risk factors. Further studies may need to be conducted to explore the related issues.

### Strength and limitations

This study uses high-quality cancer data from 186 countries to conduct an extensive analysis of the disease burden, risk factors, and temporal trends of mesothelioma. Despite that, a few limitations should be noted in this study. Due to the substandard quality of cancer data, registry coverage, and analytical capacity, underreported or misclassified cancer cancers may possibly happen, especially in low- and low-middle-income countries (LMICS). Also, the risk factor association might be overestimated or underestimated because of the confounding. Since our results were mainly based on univariate analysis, non-linear associations between the examined factors and ureteral cancer were not accessed. In addition, cancer registries may change in some countries over time, so direct comparisons may not be suitable. However, results based on comparisons of nations, regions, and sexes at the same period should be comparatively reliable.

## Conclusions

Although ureteral cancer was relatively rare, the number of cases reported was rising over the world. The association between smoking, alcohol drinking, and the consumption of herbal medicine containing aristolochic acid with ureteral cancer was well established in past studies. The rising trends among females were more evident compared with the other subgroups, especially in European countries. Due to the scarcity of ureteral cancer cases, very limited research has been done to examine the biological mechanism behind the association. Further studies could be conducted to examine the recent trend in European countries and confirm the relationship of the risk factors identified in our study, but not previously proved. Also, the association of herbal medication usage and incidence of ureteral cancer should be conduct in further research to evaluate the effect of herbal medication usage and ureteral cancer trend in low and middle-low countries.

### Supplementary Information

Additional file1: Supplementary Table 1 Global incidence of ureteral cancer. Supplementary Table 2 Log transformation data association of risk factors with ureteral cancer incidence. Supplementary Table 3 Results of joinpoint regression for trend analysis. Supplementary Fig. 1 Incidence trends for individual countries. Supplementary Fig. 2 Plots of joinpoint regression for trend analysis. 

## Data Availability

The data that support findings of this study are available from the corresponding author, upon reasonable request. The data supporting findings can be found in GLOBOCAN database, GBD database, within the manuscript, and within the supplementary materials.

## Abbreviations

AAPC: Average Annual Percentage Change
ASR: Age-standardized rates
CI: Confidence interval
CI5 Plus: Cancer Incidence in Five Continents Plus
GBD: Global Burden of Disease
GDP: Gross domestic products
GLOBOCAN: Global Cancer Observatory
HDI: Human development index
WHO: World Health Organization
