# Supplementary material for: Global burden, risk factors, and temporal trends of ureteral cancer: a comprehensive analysis of cancer registries
Source: BMC Med. 2024 Jun 24;22:264. doi: 10.1186/s12916-024-03485-x (PMC11197334; doi:10.1186/s12916-024-03485-x)
Supplement: Supplementary file 1 — Additional file1: Supplementary Table 1 Global incidence of ureteral cancer. Supplementary Table 2 Log transformation data association of risk factors with ureteral cancer incidence. Supplementary Table 3 Results of joinpoint regression for trend analysis. Supplementary Fig. 1 Incidence trends for individual countries. Supplementary Fig. 2 Plots of joinpoint regression for trend analysis. [file 12916_2024_3485_MOESM1_ESM.docx]

**Supplementary Legend**

**Supplementary Table 1.** Global incidence of ureter cancer

**Supplementary Table 2.** Log transformation data association of risk factors with ureteral cancer incidence

**Supplementary Table 3.** Results of Joinpoint regression for trend analysis

**Supplementary Figure 1.** Incidence trends for individual countries

**Supplementary Figure 2.** Plots of Joinpoint regression for trend analysis

### **Table 1** Global incidence of ureter cancer

1. Sex

| Region | Both sexes | | Males | | Females | |
| --- | --- | --- | --- | --- | --- | --- |
|  | **New cases** | **ASR** | **New cases** | **ASR** | **New cases** | **ASR*** |
| World | 22,588 | 23.4 | 13,082 | 29.8 | 9,506 | 18.3 |
| Asia | 14,202 | 26.0 | 7,789 | 31.2 | 6,413 | 22.2 |
| Eastern Asia | 12,912 | 46.5 | 7,032 | 56.1 | 5,880 | 39.9 |
| China | 8,336 | 36.9 | 4,318 | 39.0 | 4,018 | 34.6 |
| Japan | 3,857 | 97.6 | 2,289 | 127.0 | 1,568 | 69.8 |
| Korea, Democratic Republic of | 149 | 38.6 | 72 | 49.0 | 77 | 35.5 |
| Korea, Republic of | 560 | 55.9 | 350 | 74.9 | 210 | 39.5 |
| Mongolia | 10 | 30.8 | 3 | 25.6 | 7 | 47.0 |
| South-Eastern Asia | 589 | 8.2 | 319 | 9.2 | 270 | 7.0 |
| Brunei Darussalam | 1 | 13.0 | 1 | 27.8 | 0 | 12.7 |
| Cambodia | 7 | 6.5 | 4 | 8.2 | 3 | 4.5 |
| Indonesia | 172 | 7.2 | 111 | 8.7 | 61 | 4.5 |
| Lao People's Democratic Republic | 4 | 6.6 | 2 | 7.2 | 2 | 5.7 |
| Malaysia | 60 | 18.7 | 22 | 13.1 | 38 | 22.9 |
| Myanmar | 29 | 5.3 | 13 | 5.9 | 16 | 5.4 |
| Philippines | 26 | 2.8 | 10 | 2.5 | 16 | 3.0 |
| Singapore | 22 | 20.9 | 11 | 21.0 | 11 | 20.3 |
| Thailand | 187 | 14.0 | 83 | 14.3 | 104 | 14.4 |
| Timor-Leste | 0 | 5.3 | 0 | 6.3 | 0 | 3.1 |
| Viet Nam | 81 | 7.8 | 62 | 12.4 | 19 | 3.3 |
| South-Central Asia | 482 | 2.6 | 281 | 3.2 | 201 | 2.0 |
| Afghanistan | 8 | 4.0 | 5 | 5.3 | 3 | 3.0 |
| Bangladesh | 25 | 1.8 | 15 | 2.1 | 10 | 1.4 |
| Bhutan | 0 | 0.9 | 0 | 0.6 | 0 | 1.5 |
| India | 315 | 2.4 | 181 | 2.7 | 134 | 2.1 |
| Iran, Islamic Republic of | 34 | 1.9 | 18 | 4.1 | 16 | 3.8 |
| Kazakhstan | 13 | 6.2 | 8 | 9.5 | 5 | 3.9 |
| Kyrgyzstan | 2 | 3.9 | 1 | 5.4 | 1 | 3.0 |
| Maldives | 0 | 1.9 | 0 | 3.1 | 0 | 0.0 |
| Nepal | 5 | 1.9 | 3 | 2.3 | 2 | 1.5 |
| Pakistan | 57 | 3.7 | 34 | 4.4 | 23 | 2.8 |
| Sri Lanka | 7 | 2.6 | 5 | 3.6 | 2 | 1.7 |
| Tajikistan | 2 | 2.8 | 1 | 3.8 | 1 | 1.8 |
| Turkmenistan | 3 | 5.5 | 2 | 7.3 | 1 | 4.4 |
| Uzbekistan | 11 | 4.0 | 8 | 5.8 | 3 | 2.2 |
| Western Asia | 219 | 10.4 | 157 | 15.1 | 62 | 6.3 |
| Armenia | 5 | 10.5 | 3 | 17.1 | 2 | 6.4 |
| Azerbaijan | 9 | 7.5 | 6 | 10.8 | 3 | 5.2 |
| Bahrain | 0 | 6.6 | 0 | 8.6 | 0 | 3.8 |
| Gaza Strip and West Bank | 3 | 8.1 | 2 | 11.6 | 1 | 4.8 |
| Georgia | 7 | 9.8 | 5 | 16.8 | 2 | 4.8 |
| Iraq | 19 | 7.5 | 11 | 9.8 | 8 | 6.2 |
| Israel | 28 | 21.7 | 18 | 29.7 | 10 | 14.7 |
| Jordan | 6 | 8.1 | 5 | 14.2 | 1 | 2.6 |
| Kuwait | 2 | 2.7 | 1 | 7.5 | 1 | 6.9 |
| Lebanon | 8 | 10.2 | 5 | 13.3 | 3 | 7.7 |
| Oman | 2 | 5.9 | 1 | 7.4 | 1 | 3.9 |
| Qatar | 2 | 12.8 | 2 | 24.8 | 0 | 4.8 |
| Saudi Arabia | 13 | 4.9 | 8 | 5.1 | 5 | 4.2 |
| Syrian Arab Republic | 12 | 8.8 | 8 | 12.3 | 4 | 6.0 |
| Turkey | 96 | 9.5 | 77 | 16.9 | 19 | 3.4 |
| United Arab Emirates | 3 | 7.1 | 2 | 7.3 | 1 | 7.3 |
| Yemen | 4 | 2.5 | 3 | 3.7 | 1 | 1.5 |
| Oceania | 215 | 31.5 | 137 | 41.5 | 78 | 21.9 |
| Australia | 188 | 38.3 | 119 | 49.7 | 69 | 27.1 |
| Fiji | 0 | 6.5 | 0 | 7.9 | 0 | 6.0 |
| France, New Caledonia | 1 | 33.1 | 1 | 47.4 | 0 | 17.1 |
| French Polynesia | 0 | 20.0 | 0 | 27.5 | 0 | 11.1 |
| Guam | 0 | 11.0 | 0 | 18.6 | 0 | 2.5 |
| New Zealand | 22 | 23.0 | 14 | 30.9 | 8 | 15.8 |
| Papua New Guinea | 4 | 8.3 | 3 | 11.7 | 1 | 4.8 |
| Samoa | 0 | 29.5 | 0 | 30.6 | 0 | 31.3 |
| Solomon Islands | 0 | 3.4 | 0 | 5.3 | 0 | 0.7 |
| Vanuatu | 0 | 2.5 | 0 | 4.2 | 0 | 0.0 |
| Northern America | 2,328 | 32.1 | 1,399 | 40.4 | 929 | 25.0 |
| Canada | 286 | 35.4 | 172 | 43.7 | 114 | 27.3 |
| United States of America | 2,042 | 31.9 | 1,227 | 40.2 | 815 | 24.8 |
| Latin America and the Caribbean | 416 | 5.6 | 213 | 7.0 | 203 | 4.6 |
| Central America & Caribbean | 121 | 5.7 | 56 | 6.0 | 65 | 5.7 |
| Bahamas | 0 | 2.4 | 0 | 2.3 | 0 | 2.7 |
| Barbados | 0 | 5.3 | 0 | 6.1 | 0 | 3.6 |
| Belize | 0 | 0.2 | 0 | 0.0 | 0 | 0.7 |
| Costa Rica | 2 | 0.6 | 2 | 5.9 | 0 | 1.1 |
| Cuba | 2 | 11.3 | 1 | 11.4 | 1 | 11.6 |
| Dominican Republic | 3 | 2.9 | 1 | 2.5 | 2 | 3.7 |
| El Salvador | 3 | 3.0 | 1 | 2.8 | 2 | 3.7 |
| France, Guadeloupe | 0 | 5.5 | 0 | 6.1 | 0 | 4.9 |
| France, Martinique | 0 | 6.2 | 0 | 6.1 | 0 | 7.0 |
| Guatemala | 3 | 2.5 | 1 | 2.2 | 2 | 3.1 |
| Haiti | 3 | 3.5 | 1 | 3.2 | 2 | 4.3 |
| Honduras | 3 | 3.7 | 1 | 3.5 | 2 | 4.5 |
| Jamaica | 2 | 4.2 | 1 | 3.6 | 1 | 5.4 |
| Mexico | 87 | 6.3 | 41 | 6.5 | 46 | 6.2 |
| Nicaragua | 2 | 3.1 | 1 | 3.5 | 1 | 2.8 |
| Panama | 2 | 4.5 | 1 | 4.3 | 1 | 5.2 |
| Puerto Rico | 7 | 9.7 | 3 | 10.5 | 4 | 9.7 |
| Saint Lucia | 0 | 3.6 | 0 | 3.0 | 0 | 5.1 |
| Trinidad and Tobago | 2 | 7.1 | 1 | 7.3 | 1 | 7.0 |
| South America | 295 | 5.6 | 157 | 7.4 | 138 | 4.1 |
| Argentina | 34 | 5.5 | 26 | 9.4 | 8 | 2.5 |
| Bolivia, Plurinational State of | 3 | 2.2 | 1 | 2.2 | 2 | 2.4 |
| Brazil | 130 | 4.7 | 49 | 3.9 | 81 | 5.3 |
| Chile | 64 | 20.5 | 50 | 36.4 | 14 | 8.3 |
| Colombia | 9 | 1.5 | 3 | 1.1 | 6 | 1.9 |
| Ecuador | 5 | 0.9 | 2 | 2.2 | 3 | 2.5 |
| French Guiana | 1 | 22.1 | 0 | 7.2 | 1 | 41.8 |
| Guyana | 0 | 1.2 | 0 | 1.0 | 0 | 1.8 |
| Paraguay | 2 | 2.7 | 1 | 3.8 | 1 | 1.6 |
| Peru | 21 | 5.0 | 9 | 4.7 | 12 | 5.8 |
| Suriname | 0 | 2.1 | 0 | 3.5 | 0 | 0.8 |
| Uruguay | 9 | 15.8 | 6 | 23.3 | 3 | 10.0 |
| Venezuela, Bolivarian Republic of | 17 | 5.3 | 10 | 7.0 | 7 | 3.9 |
| Europe | 4,682 | 28.4 | 3,093 | 41.8 | 1,589 | 17.7 |
| Northern Europe | 927 | 40.9 | 579 | 54.0 | 348 | 29.1 |
| Denmark | 40 | 30.3 | 26 | 40.5 | 14 | 20.5 |
| Estonia | 10 | 33.5 | 5 | 44.2 | 5 | 25.0 |
| Finland | 49 | 34.2 | 32 | 48.6 | 17 | 21.6 |
| Iceland | 3 | 45.7 | 2 | 47.8 | 1 | 42.5 |
| Ireland | 29 | 33.9 | 18 | 42.6 | 11 | 25.5 |
| Latvia | 11 | 25.4 | 6 | 34.9 | 5 | 19.0 |
| Lithuania | 7 | 11.8 | 4 | 16.1 | 3 | 9.0 |
| Norway | 48 | 43.3 | 31 | 57.0 | 17 | 29.9 |
| Sweden | 94 | 40.4 | 63 | 54.5 | 31 | 26.9 |
| United Kingdom | 636 | 41.6 | 392 | 54.0 | 244 | 30.8 |
| Western Europe | 1,898 | 36.4 | 1,250 | 50.9 | 648 | 23.8 |
| Austria | 49 | 23.4 | 29 | 30.0 | 20 | 17.8 |
| Belgium | 128 | 48.4 | 84 | 67.7 | 44 | 31.9 |
| France | 720 | 46.7 | 497 | 70.2 | 223 | 26.8 |
| Germany | 769 | 35.3 | 489 | 48.4 | 280 | 24.1 |
| Luxembourg | 3 | 22.2 | 2 | 32.2 | 1 | 12.8 |
| Switzerland | 47 | 22.5 | 29 | 29.5 | 18 | 16.9 |
| The Netherlands | 182 | 45.0 | 120 | 61.0 | 62 | 30.4 |
| Southern Europe | 1,277 | 33.5 | 902 | 50.6 | 375 | 19.0 |
| Albania | 8 | 14.6 | 6 | 21.5 | 2 | 8.1 |
| Bosnia and Herzegovina | 14 | 20.1 | 9 | 28.7 | 5 | 13.4 |
| Croatia | 34 | 15.2 | 22 | 20.7 | 12 | 10.2 |
| Cyprus | 43 | 17.3 | 28 | 25.8 | 15 | 10.7 |
| Greece | 104 | 40.9 | 80 | 67.4 | 24 | 16.5 |
| Italy | 602 | 37.3 | 410 | 54.8 | 192 | 22.8 |
| Malta | 2 | 13.9 | 1 | 13.9 | 1 | 15.1 |
| Montenegro | 3 | 28.7 | 2 | 43.2 | 1 | 16.5 |
| North Macedonia | 6 | 13.7 | 3 | 14.6 | 3 | 12.3 |
| Portugal | 55 | 20.4 | 36 | 30.6 | 19 | 12.6 |
| Serbia | 53 | 30.4 | 35 | 44.4 | 18 | 19.1 |
| Slovenia | 15 | 30.3 | 10 | 42.5 | 5 | 19.2 |
| Spain | 338 | 31.0 | 260 | 50.8 | 78 | 13.8 |
| Central and Eastern Europe | 580 | 12.8 | 362 | 19.6 | 218 | 8.2 |
| Belarus | 17 | 10.1 | 13 | 18.8 | 4 | 4.4 |
| Bulgaria | 24 | 15.7 | 16 | 23.1 | 8 | 9.9 |
| Czechia | 4 | 2.4 | 2 | 2.0 | 2 | 3.1 |
| Hungary | 38 | 18.3 | 22 | 25.5 | 16 | 13.2 |
| Poland | 99 | 11.9 | 61 | 17.1 | 38 | 8.1 |
| Republic of Moldova | 6 | 9.4 | 4 | 15.9 | 2 | 4.4 |
| Romania | 54 | 13.9 | 38 | 21.8 | 16 | 7.0 |
| Russian Federation | 245 | 9.6 | 145 | 14.3 | 100 | 6.5 |
| Slovakia | 28 | 26.7 | 18 | 39.0 | 10 | 17.1 |
| Ukraine | 65 | 8.3 | 43 | 13.4 | 22 | 4.9 |
| Africa | 745 | 6.5 | 451 | 8.7 | 294 | 5.1 |
| Northern Africa | 444 | 20.4 | 249 | 25.2 | 195 | 17.7 |
| Algeria | 70 | 16.9 | 43 | 21.0 | 27 | 12.6 |
| Egypt | 248 | 29.0 | 136 | 36.1 | 112 | 25.6 |
| Libya | 11 | 20.9 | 7 | 28.4 | 4 | 13.7 |
| Morocco | 56 | 14.0 | 33 | 17.7 | 23 | 11.5 |
| Sudan | 27 | 7.3 | 10 | 7.4 | 17 | 10.4 |
| Tunisia | 32 | 21.3 | 20 | 28.6 | 12 | 14.4 |
| Sub-Saharan Africa | 301 | 1.5 | 202 | 2.3 | 99 | 1.0 |
| Angola | 3 | 1.4 | 2 | 1.9 | 1 | 0.9 |
| Benin | 0 | 0.8 | 0 | 1.4 | 0 | 0.3 |
| Botswana | 0 | 0.8 | 0 | 1.0 | 0 | 0.7 |
| Burkina Faso | 3 | 2.8 | 2 | 3.5 | 1 | 2.1 |
| Burundi | 0 | 1.2 | 0 | 1.6 | 0 | 0.9 |
| Cabo Verde | 0 | 2.7 | 0 | 5.1 | 0 | 1.1 |
| Cameroon | 1 | 0.7 | 1 | 1.2 | 0 | 0.4 |
| Central African Republic | 0 | 0.9 | 0 | 1.3 | 0 | 0.5 |
| Chad | 1 | 1.1 | 1 | 1.7 | 0 | 0.7 |
| Comoros | 0 | 2.0 | 0 | 3.0 | 0 | 1.3 |
| Congo, Democratic Republic of | 5 | 0.9 | 3 | 1.2 | 2 | 0.6 |
| Congo, Republic of | 0 | 0.5 | 0 | 0.7 | 0 | 0.4 |
| Côte d'Ivoire | 9 | 8.7 | 7 | 15.8 | 2 | 3.9 |
| Djibouti | 0 | 1.5 | 0 | 2.4 | 0 | 0.8 |
| Equatorial Guinea | 0 | 0.6 | 0 | 1.0 | 0 | 0.2 |
| Eritrea | 0 | 1.7 | 0 | 2.6 | 0 | 1.0 |
| Eswatini | 0 | 0.8 | 0 | 1.1 | 0 | 0.4 |
| Ethiopia | 13 | 1.8 | 9 | 2.8 | 4 | 1.1 |
| France, La Réunion | 0 | 3.2 | 0 | 6.0 | 0 | 1.2 |
| Gabon | 0 | 1.1 | 0 | 1.3 | 0 | 0.8 |
| Ghana | 3 | 1.1 | 2 | 1.3 | 1 | 0.9 |
| Guinea | 0 | 0.5 | 0 | 0.7 | 0 | 0.3 |
| Guinea-Bissau | 0 | 0.7 | 0 | 1.0 | 0 | 0.5 |
| Kenya | 19 | 1.2 | 2 | 1.9 | 17 | 10.1 |
| Lesotho | 0 | 0.7 | 0 | 0.7 | 0 | 0.7 |
| Liberia | 0 | 0.6 | 0 | 0.7 | 0 | 0.6 |
| Madagascar | 3 | 2.2 | 2 | 2.9 | 1 | 1.5 |
| Malawi | 3 | 2.7 | 2 | 3.5 | 1 | 2.0 |
| Mali | 3 | 3.2 | 2 | 4.3 | 1 | 2.4 |
| Mauritania | 0 | 1.2 | 0 | 1.8 | 0 | 0.8 |
| Mauritius | 0 | 2.1 | 0 | 3.6 | 0 | 0.9 |
| Mozambique | 4 | 2.3 | 2 | 3.1 | 2 | 1.8 |
| Namibia | 0 | 2.2 | 0 | 3.6 | 0 | 1.2 |
| Niger | 2 | 1.3 | 1 | 1.9 | 1 | 0.8 |
| Nigeria | 14 | 1.3 | 9 | 1.7 | 5 | 0.9 |
| Rwanda | 0 | 0.7 | 0 | 0.7 | 0 | 0.7 |
| Sao Tome and Principe | 0 | 3.9 | 0 | 7.4 | 0 | 1.3 |
| Senegal | 2 | 1.5 | 1 | 2.1 | 1 | 1.1 |
| Sierra Leone | 0 | 1.3 | 0 | 1.9 | 0 | 0.9 |
| Somalia | 2 | 1.6 | 1 | 2.5 | 1 | 1.0 |
| South Africa | 178 | 20.9 | 141 | 68.8 | 37 | 13.0 |
| South Sudan | 1 | 1.2 | 1 | 1.8 | 0 | 0.8 |
| Tanzania, United Republic of | 6 | 1.9 | 4 | 2.7 | 2 | 1.3 |
| The Republic of the Gambia | 0 | 0.6 | 0 | 1.0 | 0 | 0.2 |
| Togo | 0 | 1.0 | 0 | 1.5 | 0 | 0.6 |
| Uganda | 17 | 1.8 | 5 | 4.6 | 12 | 7.9 |
| Zambia | 1 | 1.6 | 1 | 2.4 | 0 | 1.0 |
| Zimbabwe | 8 | 1.4 | 1 | 1.7 | 7 | 13.7 |

*ASR: per 10,000,000 persons

1. Age

| Region | Young | | Old | |
| --- | --- | --- | --- | --- |
|  | **New cases** | **ASR** | **New cases** | **ASR** |
| World | 692 | 1.6 | 12,530 | 79.1 |
| Asia | 489 | 1.8 | 8,197 | 86.9 |
| Eastern Asia | 376 | 3.3 | 7,245 | 152.6 |
| China | 312 | 3.5 | 5,318 | 127.7 |
| Japan | 43 | 5.9 | 1,487 | 306.5 |
| Korea, Democratic Republic of | 4 | 2.9 | 84 | 136.4 |
| Korea, Republic of | 16 | 5.0 | 351 | 206.1 |
| Mongolia | 1 | 3.0 | 5 | 102.9 |
| South-Eastern Asia | 37 | 1.2 | 440 | 32.6 |
| Brunei Darussalam | 0 | 2.6 | 1 | 87.7 |
| Cambodia | 1 | 0.9 | 6 | 25.1 |
| Indonesia | 13 | 0.9 | 154 | 32.3 |
| Lao People's Democratic Republic | 0 | 1.3 | 2 | 24.5 |
| Malaysia | 3 | 1.7 | 49 | 84.1 |
| Myanmar | 2 | 0.8 | 21 | 21.9 |
| Philippines | 3 | 0.5 | 13 | 7.8 |
| Singapore | 1 | 3.3 | 17 | 85.5 |
| Thailand | 7 | 1.7 | 97 | 47.6 |
| Timor-Leste | 0 | 0.2 | 0 | 17.4 |
| Viet Nam | 7 | 1.3 | 80 | 41.0 |
| South-Central Asia | 58 | 0.5 | 364 | 11.9 |
| Afghanistan | 2 | 1.2 | 6 | 18.1 |
| Bangladesh | 3 | 0.4 | 19 | 8.4 |
| Bhutan | 0 | 0.0 | 0 | 4.7 |
| India | 35 | 0.5 | 241 | 10.4 |
| Iran, Islamic Republic of | 5 | 1.0 | 17 | 11.8 |
| Kazakhstan | 1 | 1.2 | 12 | 31.5 |
| Kyrgyzstan | 0 | 0.7 | 2 | 19.2 |
| Maldives | 0 | 0.2 | 0 | 9.5 |
| Nepal | 1 | 0.4 | 4 | 8.5 |
| Pakistan | 7 | 0.8 | 45 | 17.4 |
| Sri Lanka | 1 | 0.7 | 6 | 11.2 |
| Tajikistan | 0 | 1.0 | 1 | 10.7 |
| Turkmenistan | 1 | 2.1 | 2 | 23.5 |
| Uzbekistan | 2 | 1.0 | 9 | 18.5 |
| Western Asia | 18 | 1.2 | 148 | 42.3 |
| Armenia | 0 | 0.9 | 3 | 39.1 |
| Azerbaijan | 1 | 1.0 | 6 | 29.5 |
| Bahrain | 0 | 0.5 | 0 | 25.7 |
| Gaza Strip and West Bank | 0 | 1.1 | 1 | 31.2 |
| Georgia | 0 | 0.9 | 5 | 43.6 |
| Iraq | 2 | 1.2 | 11 | 30.4 |
| Israel | 1 | 2.1 | 17 | 86.7 |
| Jordan | 1 | 1.1 | 4 | 35.8 |
| Kuwait | 0 | 0.3 | 1 | 16.6 |
| Lebanon | 0 | 0.9 | 5 | 42.2 |
| Oman | 0 | 1.1 | 1 | 19.6 |
| Qatar | 0 | 0.3 | 2 | 79.4 |
| Saudi Arabia | 2 | 0.9 | 12 | 27.7 |
| Syrian Arab Republic | 1 | 1.1 | 8 | 35.7 |
| Turkey | 10 | 2.1 | 68 | 40.2 |
| United Arab Emirates | 0 | 0.3 | 2 | 26.2 |
| Yemen | 0 | 0.4 | 2 | 9.3 |
| Oceania | 4 | 1.8 | 95 | 91.0 |
| Australia | 3 | 2.0 | 81 | 109.8 |
| Fiji | 0 | 0.6 | 0 | 21.8 |
| France, New Caledonia | 0 | 0.3 | 1 | 104.8 |
| French Polynesia | 0 | 1.0 | 0 | 68.5 |
| Guam | 0 | 0.6 | 0 | 27.6 |
| New Zealand | 1 | 4.1 | 10 | 68.7 |
| Papua New Guinea | 0 | 0.2 | 3 | 26.7 |
| Samoa | 0 | 1.3 | 0 | 103.3 |
| Solomon Islands | 0 | 0.0 | 0 | 13.4 |
| Vanuatu | 0 | 0.0 | 0 | 10.9 |
| Northern America | 45 | 2.4 | 1,161 | 100.1 |
| Canada | 5 | 2.8 | 144 | 113.8 |
| United States of America | 40 | 2.4 | 1,017 | 99.3 |
| Latin America and the Caribbean | 33 | 0.9 | 201 | 19.6 |
| Central America & Caribbean | 4 | 0.5 | 59 | 17.9 |
| Bahamas | 0 | 0.2 | 0 | 7.3 |
| Barbados | 0 | 1.0 | 0 | 13.7 |
| Belize | 0 | 0.1 | 0 | 0.0 |
| Costa Rica | 0 | 0.4 | 0 | 3.5 |
| Cuba | 0 | 1.2 | 1 | 33.3 |
| Dominican Republic | 0 | 0.3 | 1 | 7.1 |
| El Salvador | 0 | 0.3 | 1 | 7.7 |
| France, Guadeloupe | 0 | 0.3 | 0 | 18.8 |
| France, Martinique | 0 | 0.4 | 0 | 18.6 |
| Guatemala | 0 | 0.3 | 1 | 7.3 |
| Haiti | 0 | 0.3 | 1 | 8.1 |
| Honduras | 0 | 0.3 | 1 | 10.0 |
| Jamaica | 0 | 0.3 | 1 | 11.8 |
| Mexico | 4 | 0.5 | 46 | 20.2 |
| Nicaragua | 0 | 0.3 | 1 | 9.3 |
| Panama | 0 | 0.4 | 1 | 13.3 |
| Puerto Rico | 0 | 0.6 | 3 | 26.9 |
| Saint Lucia | 0 | 0.5 | 0 | 11.0 |
| Trinidad and Tobago | 0 | 0.5 | 1 | 23.0 |
| South America | 29 | 1.1 | 142 | 20.2 |
| Argentina | 4 | 1.5 | 22 | 22.9 |
| Bolivia, Plurinational State of | 0 | 0.3 | 1 | 7.6 |
| Brazil | 13 | 1.1 | 46 | 10.1 |
| Chile | 2 | 1.7 | 41 | 84.8 |
| Colombia | 3 | 1.0 | 6 | 6.1 |
| Ecuador | 1 | 0.6 | 1 | 3.1 |
| French Guiana | 0 | 16.9 | 0 | 77.0 |
| Guyana | 0 | 0.0 | 0 | 5.4 |
| Paraguay | 0 | 0.5 | 1 | 10.1 |
| Peru | 3 | 1.4 | 6 | 9.6 |
| Suriname | 0 | 0.5 | 0 | 6.1 |
| Uruguay | 1 | 4.6 | 7 | 79.3 |
| Venezuela, Bolivarian Republic of | 2 | 1.3 | 11 | 19.2 |
| Europe | 112 | 2.7 | 2,413 | 98.5 |
| Northern Europe | 20 | 3.6 | 442 | 134.5 |
| Denmark | 1 | 4.8 | 23 | 116.9 |
| Estonia | 0 | 3.1 | 6 | 141.1 |
| Finland | 1 | 2.6 | 23 | 111.2 |
| Iceland | 0 | 2.5 | 2 | 184.4 |
| Ireland | 2 | 5.2 | 18 | 133.2 |
| Latvia | 1 | 6.1 | 5 | 84.0 |
| Lithuania | 0 | 2.3 | 4 | 48.3 |
| Norway | 1 | 2.5 | 24 | 137.6 |
| Sweden | 2 | 3.6 | 45 | 135.2 |
| United Kingdom | 12 | 3.4 | 292 | 135.2 |
| Western Europe | 33 | 3.2 | 915 | 125.6 |
| Austria | 1 | 1.6 | 24 | 80.2 |
| Belgium | 2 | 3.9 | 64 | 168.5 |
| France | 12 | 3.6 | 345 | 155.9 |
| Germany | 16 | 3.8 | 368 | 126.3 |
| Luxembourg | 0 | 1.7 | 1 | 71.1 |
| Switzerland | 0 | 0.8 | 22 | 75.7 |
| The Netherlands | 2 | 2.7 | 91 | 147.3 |
| Southern Europe | 33 | 3.3 | 671 | 124.8 |
| Albania | 0 | 1.0 | 4 | 49.7 |
| Bosnia and Herzegovina | 1 | 3.1 | 9 | 74.2 |
| Croatia | 1 | 1.3 | 21 | 60.3 |
| Cyprus | 2 | 3.4 | 32 | 86.1 |
| Greece | 2 | 3.2 | 57 | 161.0 |
| Italy | 12 | 3.4 | 296 | 136.0 |
| Malta | 0 | 2.0 | 1 | 43.8 |
| Montenegro | 0 | 1.6 | 2 | 122.0 |
| North Macedonia | 0 | 1.1 | 3 | 49.3 |
| Portugal | 1 | 2.2 | 26 | 71.7 |
| Serbia | 2 | 4.1 | 35 | 117.0 |
| Slovenia | 1 | 5.4 | 7 | 96.4 |
| Spain | 11 | 3.8 | 178 | 116.8 |
| Central and Eastern Europe | 26 | 1.8 | 385 | 51.3 |
| Belarus | 1 | 2.6 | 13 | 42.7 |
| Bulgaria | 2 | 5.2 | 15 | 57.6 |
| Czechia | 0 | 0.2 | 1 | 6.0 |
| Hungary | 2 | 2.5 | 24 | 73.5 |
| Poland | 3 | 1.5 | 62 | 47.6 |
| Republic of Moldova | 0 | 0.9 | 5 | 41.3 |
| Romania | 2 | 1.9 | 37 | 56.7 |
| Russian Federation | 10 | 1.2 | 160 | 35.4 |
| Slovakia | 2 | 5.5 | 18 | 101.6 |
| Ukraine | 4 | 1.5 | 50 | 36.2 |
| Africa | 9 | 0.2 | 463 | 23.0 |
| Northern Africa | 9 | 0.7 | 242 | 67.4 |
| Algeria | 1 | 0.5 | 39 | 56.5 |
| Egypt | 5 | 1.0 | 141 | 98.2 |
| Libya | 0 | 0.7 | 5 | 62.7 |
| Morocco | 1 | 0.4 | 31 | 44.7 |
| Sudan | 1 | 0.5 | 9 | 20.0 |
| Tunisia | 1 | 0.9 | 17 | 66.4 |
| Sub-Saharan Africa | 0 | 0.1 | 221 | 5.9 |
| Angola | 0 | 0.1 | 1 | 5.2 |
| Benin | 0 | 0.0 | 0 | 3.6 |
| Botswana | 0 | 0.0 | 0 | 2.8 |
| Burkina Faso | 0 | 0.2 | 2 | 12.2 |
| Burundi | 0 | 0.1 | 0 | 3.9 |
| Cabo Verde | 0 | 0.1 | 0 | 14.7 |
| Cameroon | 0 | 0.1 | 1 | 2.9 |
| Central African Republic | 0 | 0.0 | 0 | 3.2 |
| Chad | 0 | 0.1 | 1 | 4.4 |
| Comoros | 0 | 0.1 | 0 | 7.0 |
| Congo, Democratic Republic of | 0 | 0.0 | 2 | 2.9 |
| Congo, Republic of | 0 | 0.0 | 0 | 1.6 |
| Côte d'Ivoire | 0 | 0.3 | 6 | 41.7 |
| Djibouti | 0 | 0.1 | 0 | 6.1 |
| Equatorial Guinea | 0 | 0.0 | 0 | 2.8 |
| Eritrea | 0 | 0.1 | 0 | 6.2 |
| Eswatini | 0 | 0.0 | 0 | 4.5 |
| Ethiopia | 0 | 0.1 | 7 | 6.9 |
| France, La Réunion | 0 | 0.1 | 0 | 14.3 |
| Gabon | 0 | 0.0 | 0 | 4.8 |
| Ghana | 0 | 0.1 | 1 | 4.3 |
| Guinea | 0 | 0.0 | 0 | 1.3 |
| Guinea-Bissau | 0 | 0.0 | 0 | 3.3 |
| Kenya | 0 | 0.0 | 2 | 4.9 |
| Lesotho | 0 | 0.0 | 0 | 3.2 |
| Liberia | 0 | 0.0 | 0 | 1.9 |
| Madagascar | 0 | 0.1 | 2 | 7.8 |
| Malawi | 0 | 0.3 | 1 | 9.4 |
| Mali | 0 | 0.2 | 2 | 13.8 |
| Mauritania | 0 | 0.1 | 0 | 5.3 |
| Mauritius | 0 | 0.1 | 0 | 8.2 |
| Mozambique | 0 | 0.1 | 2 | 9.0 |
| Namibia | 0 | 0.1 | 0 | 9.4 |
| Niger | 0 | 0.1 | 1 | 5.3 |
| Nigeria | 0 | 0.0 | 9 | 5.4 |
| Rwanda | 0 | 0.0 | 0 | 2.0 |
| Sao Tome and Principe | 0 | 0.0 | 0 | 25.6 |
| Senegal | 0 | 0.1 | 1 | 6.3 |
| Sierra Leone | 0 | 0.1 | 0 | 5.8 |
| Somalia | 0 | 0.1 | 1 | 6.3 |
| South Africa | 0 | 0.1 | 173 | 200.8 |
| South Sudan | 0 | 0.1 | 0 | 4.4 |
| Tanzania, United Republic of | 0 | 0.1 | 3 | 6.3 |
| The Republic of the Gambia | 0 | 0.0 | 0 | 2.6 |
| Togo | 0 | 0.0 | 0 | 3.9 |
| Uganda | 0 | 0.0 | 1 | 2.9 |
| Zambia | 0 | 0.1 | 1 | 6.7 |
| Zimbabwe | 0 | 0.0 | 1 | 5.1 |

*ASR: per 10,000,000 persons

**Supplementary Table 2.** **Log transformation data association of risk factors with ureteral cancer incidence**

| **Outcome** | **Risk factor** | **Overall** | | | |
| --- | --- | --- | --- | --- | --- |
|  |  | ***β*** | ***95% CI*** | | ***P*** |
| **All Sexes and ages** | HDI | 0.501 | 0.398 | 0.605 | <0.001* |
|  | GDP per capita | 0.315 | 0.230 | 0.401 | <0.001* |
|  | Smoking | 0.177 | 0.154 | 0.200 | <0.001* |
|  | Alcohol drinking | 0.140 | 0.108 | 0.171 | <0.001* |
|  | Dietary | 0.024 | 0.006 | 0.041 | 0.008* |
|  | Physical inactivity | 0.124 | 0.064 | 0.184 | <0.001* |
|  | Obesity | 0.041 | 0.024 | 0.058 | <0.001* |
|  | Hypertension | 0.056 | 0.035 | 0.077 | <0.001* |
|  | Diabetes | 0.094 | 0.061 | 0.128 | <0.001* |
|  | Lipid | 0.079 | 0.067 | 0.091 | <0.001* |
|  |  |  |  |  |  |
| **Male** | HDI | 0.500 | 0.397 | 0.603 | <0.001* |
|  | GDP per capita | 0.312 | 0.228 | 0.397 | <0.001* |
|  | Smoking | 0.114 | 0.095 | 0.132 | <0.001* |
|  | Alcohol drinking | 0.096 | 0.074 | 0.119 | <0.001* |
|  | Dietary | 0.027 | 0.014 | 0.040 | <0.001* |
|  | Physical inactivity | 0.068 | 0.007 | 0.129 | 0.030* |
|  | Obesity | 0.052 | 0.036 | 0.068 | <0.001* |
|  | Hypertension | 0.058 | 0.038 | 0.077 | <0.001* |
|  | Diabetes | 0.091 | 0.059 | 0.122 | <0.001* |
|  | Lipid | 0.075 | 0.063 | 0.086 | <0.001* |
|  |  |  |  |  |  |
|  | HDI | 0.483 | 0.379 | 0.588 | <0.001* |
|  | GDP per capita | 0.295 | 0.208 | 0.381 | <0.001* |
|  | Smoking | 0.151 | 0.120 | 0.182 | <0.001* |
|  | Alcohol drinking | 0.171 | 0.125 | 0.218 | <0.001* |
|  | Dietary | 0.001 | -0.020 | 0.022 | 0.902 |
| **Female** | Physical inactivity | 0.150 | 0.096 | 0.204 | <0.001* |
|  | Obesity | 0.032 | 0.015 | 0.048 | <0.001* |
|  | Hypertension | 0.040 | 0.018 | 0.062 | <0.001* |
|  | Diabetes | 0.092 | 0.057 | 0.127 | <0.001* |
|  | Lipid | 0.077 | 0.065 | 0.090 | <0.001* |
|  |  |  |  |  |  |
| **Young** | HDI | 0.655 | 0.530 | 0.780 | <0.001* |
|  | GDP per capita | 0.325 | 0.214 | 0.436 | <0.001* |
|  | Smoking | 0.233 | 0.200 | 0.267 | <0.001* |
|  | Alcohol drinking | 0.150 | 0.111 | 0.189 | <0.001* |
|  | Dietary | 0.047 | 0.026 | 0.068 | <0.001* |
|  | Physical inactivity | 0.089 | 0.009 | 0.169 | 0.029* |
|  | Obesity | 0.041 | 0.020 | 0.062 | <0.001* |
|  | Hypertension | 0.005 | -0.038 | 0.047 | 0.834 |
|  | Diabetes | 0.183 | 0.075 | 0.292 | 0.001* |
|  | Lipid | 0.115 | 0.099 | 0.131 | <0.001* |
|  |  |  |  |  |  |
| **Old** | HDI | 0.490 | 0.388 | 0.592 | <0.001* |
|  | GDP per capita | 0.299 | 0.215 | 0.383 | <0.001* |
|  | Smoking | 0.147 | 0.127 | 0.167 | <0.001* |
|  | Alcohol drinking | 0.110 | 0.076 | 0.145 | <0.001* |
|  | Dietary | 0.014 | -0.001 | 0.029 | 0.074 |
|  | Physical inactivity | 0.098 | 0.041 | 0.155 | 0.001* |
|  | Obesity | 0.028 | 0.015 | 0.042 | <0.001* |
|  | Hypertension | 0.007 | -0.011 | 0.024 | 0.474 |
|  | Diabetes | 0.018 | -0.002 | 0.037 | 0.075 |
|  | Lipid | 0.074 | 0.062 | 0.086 | <0.001* |

The analysis was conducted using negative binomial regression model at a country level with log transformation.

β, beta coefficient. The beta coefficient can be interpreted as the change in log (incidence) associated with one percent increase of a certain risk factor.

CI, confidence interval; ASR, age-standardized rate; HDI, human development index; GDP, gross domestic products.

* p values less than 0.05.

**Supplementary Table 3. Results of Joinpoint regression for trend analysis**

a) Male

| **Region** | **AAPC** | **Lower CI** | **Upper CI** | **p-value** | **Significant** |
| --- | --- | --- | --- | --- | --- |
| ***Asia*** |  |  |  |  |  |
| China | 5.69 | -1.33 | 13.21 | 0.100 |  |
| India | 29.23 | 12.37 | 48.62 | 0.000 | * |
| Israel | -1.00 | -5.09 | 3.27 | 0.598 |  |
| Japan | 1.60 | -0.92 | 4.18 | 0.184 |  |
| Korea | 2.08 | -2.07 | 6.40 | 0.287 |  |
| Kuwait | -13.17 | -24.36 | -0.34 | 0.045 | * |
| Philippines | 1.98 | -23.24 | 35.49 | 0.892 |  |
| Thailand | 4.82 | -11.00 | 23.45 | 0.525 |  |
| Turkey | 1.20 | -7.70 | 10.95 | 0.773 |  |
| ***Oceania*** |  |  |  |  |  |
| Australia | 3.81 | -2.19 | 10.18 | 0.185 |  |
| New Zealand | 5.72 | -8.01 | 21.50 | 0.433 |  |
| ***Northern America*** |  |  |  |  |  |
| Canada | 2.58 | -0.88 | 6.17 | 0.146 |  |
| USA | -0.30 | -2.07 | 1.49 | 0.706 |  |
| ***Southern America*** |  |  |  |  |  |
| Brazil | -24.66 | -26.61 | -22.82 | <0.001 | * |
| Chile | 29.84 | 25.74 | 34.06 | 0.000 | * |
| Colombia | -9.38 | -9.57 | -9.19 | 0.000 | * |
| Ecuador | 3.87 | -5.80 | 14.55 | 0.446 |  |
| Martinique | NA | NA | NA | NA | NA |
| ***Northern Europe*** |  |  |  |  |  |
| Denmark | 5.17 | -2.89 | 13.91 | 0.183 |  |
| Estonia | 1.69 | -3.77 | 7.45 | 0.504 |  |
| Iceland | 12.52 | -13.05 | 45.61 | 0.370 |  |
| Ireland | 5.44 | -4.24 | 16.10 | 0.240 |  |
| Lithuania | -2.99 | -31.30 | 37.00 | 0.863 |  |
| Norway | -10.11 | -25.18 | 7.99 | 0.255 |  |
| United Kingdom | 3.70 | 2.28 | 5.14 | 0.000 | * |
| ***Western Europe*** |  |  |  |  |  |
| Austria | -0.88 | -6.64 | 5.23 | 0.742 |  |
| France | 3.82 | -6.69 | 15.53 | 0.491 |  |
| Germany | 4.86 | -1.39 | 11.50 | 0.113 |  |
| Netherlands | 0.60 | -5.72 | 7.34 | 0.857 |  |
| Switzerland | 15.02 | -9.32 | 45.88 | 0.249 |  |
| ***Southern Europe*** |  |  |  |  |  |
| Croatia | 7.97 | 3.70 | 12.42 | 0.002 | * |
| Cyprus | -9.09 | -17.92 | 0.70 | 0.064 |  |
| Italy | 3.77 | -1.86 | 9.71 | 0.165 |  |
| Malta | -8.14 | -20.76 | 6.49 | 0.260 |  |
| Slovenia | 2.15 | -7.23 | 12.48 | 0.624 |  |
| Spain | 5.17 | -6.51 | 18.31 | 0.402 |  |
| ***Eastern Europe*** |  |  |  |  |  |
| Bulgaria | 9.79 | 0.98 | 19.36 | 0.033 | * |
| Czech Republic | -0.29 | -4.29 | 3.87 | 0.872 |  |
| Poland | 4.14 | -17.67 | 31.72 | 0.735 |  |
| ***Africa*** |  |  |  |  |  |
| Uganda | -11.18 | -11.79 | -10.57 | 0.000 | * |

AAPC, annual percentage change; CI, confidence interval; * p values less than 0·05. NA, not available as it reported zero cases during the period and joinpoint regression could not be performed in such circumstances.

b) Female

| **Region** | **AAPC** | **Lower CI** | **Upper CI** | **p-value** | **Significant** |
| --- | --- | --- | --- | --- | --- |
| ***Asia*** |  |  |  |  |  |
| China | 2.39 | -3.84 | 9.02 | 0.411 |  |
| India | 0.96 | -17.44 | 23.48 | 0.926 |  |
| Israel | -1.92 | -12.13 | 9.47 | 0.729 |  |
| Japan | 2.88 | -2.56 | 8.63 | 0.306 |  |
| Korea | 5.82 | -0.11 | 12.10 | 0.053 |  |
| Kuwait | 2.09 | -15.12 | 22.78 | 0.826 |  |
| Philippines | -3.40 | -11.15 | 5.02 | 0.417 |  |
| Thailand | 0.60 | -8.75 | 10.91 | 0.891 |  |
| Turkey | 8.08 | -1.96 | 19.16 | 0.103 |  |
| ***Oceania*** |  |  |  |  |  |
| Australia | -0.26 | -4.50 | 4.17 | 0.894 |  |
| New Zealand | 6.33 | -16.20 | 34.92 | 0.614 |  |
| ***Northern America*** |  |  |  |  |  |
| Canada | 5.05 | 0.89 | 9.38 | 0.023 | * |
| USA | 0.54 | -8.95 | 11.02 | 0.915 |  |
| ***Southern America*** |  |  |  |  |  |
| Brazil | 19.17 | 13.59 | 25.03 | 0.000 | * |
| Chile | NA | NA | NA | NA | NA |
| Colombia | 6.85 | -3.61 | 18.45 | 0.207 |  |
| Ecuador | NA | NA | NA | NA | NA |
| Martinique | -13.17 | -24.36 | -0.34 | 0.045 | * |
| ***Northern Europe*** |  |  |  |  |  |
| Denmark | -0.01 | -12.11 | 13.76 | 0.999 |  |
| Estonia | 1.11 | -12.94 | 17.44 | 0.869 |  |
| Iceland | 7.68 | -8.29 | 26.44 | 0.319 |  |
| Ireland | 9.93 | -0.06 | 20.93 | 0.051 |  |
| Lithuania | 3.40 | -8.63 | 17.02 | 0.550 |  |
| Norway | 2.76 | -9.79 | 17.05 | 0.643 |  |
| United Kingdom | 4.75 | 0.85 | 8.81 | 0.017 | * |
| ***Western Europe*** |  |  |  |  |  |
| Austria | -0.31 | -6.51 | 6.31 | 0.915 |  |
| France | 2.03 | -5.82 | 10.54 | 0.579 |  |
| Germany | -2.56 | -9.63 | 5.06 | 0.450 |  |
| Netherlands | 3.72 | 0.55 | 6.98 | 0.026 | * |
| Switzerland | -4.80 | -16.78 | 8.90 | 0.423 |  |
| ***Southern Europe*** |  |  |  |  |  |
| Croatia | 16.43 | -4.68 | 42.21 | 0.136 |  |
| Cyprus | -16.59 | -18.84 | -14.28 | 0.000 | * |
| Italy | -2.10 | -11.80 | 8.67 | 0.652 |  |
| Malta | 30.71 | 27.08 | 34.45 | 0.000 | * |
| Slovenia | 2.69 | -12.53 | 20.55 | 0.713 |  |
| Spain | -5.05 | -15.51 | 6.70 | 0.336 |  |
| ***Eastern Europe*** |  |  |  |  |  |
| Bulgaria | 21.54 | 0.15 | 47.50 | 0.048 | * |
| Czech Republic | 1.03 | -2.42 | 4.60 | 0.516 |  |
| Poland | 11.04 | -3.68 | 28.02 | 0.128 |  |
| ***Africa*** |  |  |  |  |  |
| Uganda | -18.79 | -21.95 | -15.50 | 0.000 | * |

AAPC, annual percentage change; CI, confidence interval; * p values less than 0.05. NA, not available as it reported zero cases during the period and joinpoint regression could not be performed in such circumstances.

c) Both

| **Region** | **AAPC** | **Lower CI** | **Upper CI** | **p-value** | **Significant** |
| --- | --- | --- | --- | --- | --- |
| ***Asia*** |  |  |  |  |  |
| China | 4.47 | -0.25 | 9.42 | 0.061 |  |
| India | 31.97 | 10.33 | 57.85 | 0.002 | * |
| Israel | -0.41 | -4.03 | 3.35 | 0.806 |  |
| Japan | 2.01 | -0.02 | 4.07 | 0.052 |  |
| Korea | 3.21 | 0.71 | 5.77 | 0.018 | * |
| Kuwait | -10.57 | -24.30 | 5.64 | 0.189 |  |
| Philippines | -7.02 | -16.13 | 3.08 | 0.167 |  |
| Thailand | 5.52 | -3.26 | 15.09 | 0.192 |  |
| Turkey | 2.65 | -5.25 | 11.21 | 0.473 |  |
| ***Oceania*** |  |  |  |  |  |
| Australia | 2.19 | -2.10 | 6.68 | 0.278 |  |
| New Zealand | 2.92 | -4.50 | 10.93 | 0.401 |  |
| ***Northern America*** |  |  |  |  |  |
| Canada | 3.14 | 1.07 | 5.25 | 0.008 | * |
| USA | 0.25 | -4.28 | 4.99 | 0.915 |  |
| ***Southern America*** |  |  |  |  |  |
| Brazil | 0.50 | -4.49 | 5.75 | 0.827 |  |
| Chile | 29.84 | 25.74 | 34.06 | 0.000 | * |
| Colombia | -2.95 | -7.92 | 2.29 | 0.226 |  |
| Ecuador | 3.87 | -5.80 | 14.55 | 0.446 |  |
| Martinique | -13.17 | -24.36 | -0.34 | 0.045 | * |
| ***Northern Europe*** |  |  |  |  |  |
| Denmark | 4.27 | -2.44 | 11.43 | 0.185 |  |
| Estonia | 8.24 | -2.53 | 20.20 | 0.120 |  |
| Iceland | 5.53 | -25.46 | 49.41 | 0.762 |  |
| Ireland | 6.91 | -1.06 | 15.52 | 0.082 |  |
| Lithuania | 2.94 | -16.09 | 26.29 | 0.781 |  |
| Norway | -7.24 | -20.19 | 7.82 | 0.327 |  |
| United Kingdom | 3.72 | 2.60 | 4.86 | 0.000 | * |
| ***Western Europe*** |  |  |  |  |  |
| Austria | -0.65 | -5.55 | 4.50 | 0.773 |  |
| France | 3.83 | -4.53 | 12.92 | 0.380 |  |
| Germany | 2.56 | -3.91 | 9.47 | 0.397 |  |
| Netherlands | 2.50 | 0.25 | 4.81 | 0.034 | * |
| Switzerland | 7.82 | -8.93 | 27.66 | 0.382 |  |
| ***Southern Europe*** |  |  |  |  |  |
| Croatia | 8.41 | 5.42 | 11.49 | 0.000 | * |
| Cyprus | -9.41 | -19.26 | 1.65 | 0.083 |  |
| Italy | 2.51 | -2.04 | 7.28 | 0.244 |  |
| Malta | 0.78 | -10.63 | 13.64 | 0.886 |  |
| Slovenia | 2.37 | -5.19 | 10.54 | 0.501 |  |
| Spain | 3.78 | -4.95 | 13.30 | 0.408 |  |
| ***Eastern Europe*** |  |  |  |  |  |
| Bulgaria | 14.61 | -0.78 | 32.38 | 0.064 |  |
| Czech Republic | 0.10 | -2.75 | 3.04 | 0.937 |  |
| Poland | 15.26 | -4.31 | 38.83 | 0.116 |  |
| ***Africa*** |  |  |  |  |  |
| Uganda | -17.51 | -18.53 | -16.48 | 0.000 | * |

AAPC, annual percentage change; CI, confidence interval; * p values less than 0·05.

d) Young

| **Region** | **AAPC** | **Lower CI** | **Upper CI** | **p-value** | **Significant** |
| --- | --- | --- | --- | --- | --- |
| ***Asia*** |  |  |  |  |  |
| China | 9.21 | -9.70 | 32.08 | 0.316 |  |
| India | 3.18 | -5.05 | 12.11 | 0.461 |  |
| Israel | -4.76 | -14.80 | 6.45 | 0.342 |  |
| Japan | 0.36 | -16.49 | 20.62 | 0.965 |  |
| Korea | 1.42 | -13.26 | 18.59 | 0.840 |  |
| Kuwait | NA | NA | NA | NA | NA |
| Philippines | 8.78 | -1.91 | 20.64 | 0.111 |  |
| Thailand | 8.76 | -2.47 | 21.29 | 0.131 |  |
| Turkey | 4.41 | -8.20 | 18.75 | 0.511 |  |
| ***Oceania*** | NA | NA | NA | NA | NA |
| Australia | -8.39 | -19.21 | 3.89 | 0.147 |  |
| New Zealand | 18.67 | 0.98 | 39.45 | 0.038 | * |
| ***Northern America*** |  |  |  |  |  |
| Canada | -0.78 | -20.16 | 23.30 | 0.943 |  |
| USA | 7.38 | -4.95 | 21.31 | 0.215 |  |
| ***Southern America*** |  |  |  |  |  |
| Brazil | NA | NA | NA | NA | NA |
| Chile | NA | NA | NA | NA | NA |
| Colombia | NA | NA | NA | NA | NA |
| Ecuador | NA | NA | NA | NA | NA |
| Martinique | NA | NA | NA | NA | NA |
| ***Northern Europe*** |  |  |  |  |  |
| Denmark | 11.44 | -7.31 | 33.97 | 0.249 |  |
| Estonia | -7.00 | -19.98 | 8.09 | 0.344 |  |
| Iceland | NA | NA | NA | NA | NA |
| Ireland | -11.69 | -18.93 | -3.79 | 0.004 | * |
| Lithuania | 14.64 | 1.77 | 29.14 | 0.024 | * |
| Norway | -9.01 | -29.77 | 17.90 | 0.475 |  |
| United Kingdom | 2.86 | -6.49 | 13.13 | 0.515 |  |
| ***Western Europe*** |  |  |  |  |  |
| Austria | -0.52 | -12.89 | 13.60 | 0.930 |  |
| France | -9.09 | -34.12 | 25.44 | 0.562 |  |
| Germany | -2.35 | -11.76 | 8.06 | 0.603 |  |
| Netherlands | -0.09 | -10.76 | 11.85 | 0.985 |  |
| Switzerland | 3.97 | 3.63 | 4.31 | 0.000 | * |
| ***Southern Europe*** |  |  |  |  |  |
| Croatia | 3.87 | -1.71 | 9.76 | 0.152 |  |
| Cyprus | -3.73 | -12.70 | 6.16 | 0.446 |  |
| Italy | -6.24 | -12.98 | 1.03 | 0.082 |  |
| Malta | NA | NA | NA | NA | NA |
| Slovenia | -12.32 | -16.12 | -8.35 | 0.000 | * |
| Spain | 8.60 | -4.42 | 23.39 | 0.205 |  |
| ***Eastern Europe*** |  |  |  |  |  |
| Bulgaria | 1.02 | -12.78 | 17.01 | 0.877 |  |
| Czech Republic | -9.56 | -21.49 | 4.18 | 0.164 |  |
| Poland | 7.10 | -2.87 | 18.11 | 0.169 |  |
| ***Africa*** |  |  |  |  |  |
| Uganda | NA | NA | NA | NA | NA |

AAPC, annual percentage change; CI, confidence interval; * p values less than 0.05. NA, not available as it reported zero cases during the period and joinpoint regression could not be performed in such circumstances.

e) Old

| **Region** | **AAPC** | **Lower CI** | **Upper CI** | **p-value** | **Significant** |
| --- | --- | --- | --- | --- | --- |
| ***Asia*** |  |  |  |  |  |
| China | 3.42 | -1.29 | 8.36 | 0.135 |  |
| India | 25.98 | -2.20 | 62.27 | 0.074 |  |
| Israel | -0.08 | -5.42 | 5.56 | 0.975 |  |
| Japan | 0.33 | -2.52 | 3.27 | 0.798 |  |
| Korea | 1.86 | -0.91 | 4.72 | 0.162 |  |
| Kuwait | -14.55 | -15.90 | -13.15 | <0.001 | * |
| Philippines | -0.22 | -12.03 | 13.18 | 0.973 |  |
| Thailand | 2.27 | -6.53 | 11.90 | 0.581 |  |
| Turkey | 0.72 | -8.02 | 10.29 | 0.860 |  |
| ***Oceania*** |  |  |  |  |  |
| Australia | 1.94 | -3.44 | 7.62 | 0.437 |  |
| New Zealand | -0.12 | -8.95 | 9.56 | 0.977 |  |
| ***Northern America*** |  |  |  |  |  |
| Canada | 3.18 | 1.07 | 5.34 | 0.008 | * |
| USA | -1.24 | -4.92 | 2.59 | 0.473 |  |
| ***Southern America*** |  |  |  |  |  |
| Brazil | -4.06 | -4.17 | -3.94 | <0.001 | * |
| Chile | NA | NA | NA | NA | NA |
| Colombia | -7.87 | -11.56 | -4.02 | 0.002 | * |
| Ecuador | 3.87 | -5.80 | 14.55 | 0.446 |  |
| Martinique | NA | NA | NA | NA | NA |
| ***Northern Europe*** |  |  |  |  |  |
| Denmark | 3.12 | -5.04 | 11.99 | 0.415 |  |
| Estonia | 11.33 | -3.54 | 28.49 | 0.123 |  |
| Iceland | 4.66 | -16.09 | 30.54 | 0.686 |  |
| Ireland | 7.71 | 1.63 | 14.14 | 0.018 | * |
| Lithuania | 3.53 | -10.87 | 20.26 | 0.608 |  |
| Norway | -6.90 | -21.85 | 10.92 | 0.424 |  |
| United Kingdom | 1.49 | -0.43 | 3.45 | 0.112 |  |
| ***Western Europe*** |  |  |  |  |  |
| Austria | -0.68 | -7.11 | 6.20 | 0.820 |  |
| France | 3.21 | -7.69 | 15.40 | 0.579 |  |
| Germany | 2.23 | -4.61 | 9.57 | 0.483 |  |
| Netherlands | 0.72 | -1.86 | 3.38 | 0.540 |  |
| Switzerland | 10.06 | -16.36 | 44.84 | 0.494 |  |
| ***Southern Europe*** |  |  |  |  |  |
| Croatia | 9.30 | 5.63 | 13.10 | 0.000 | * |
| Cyprus | -11.89 | -19.56 | -3.48 | 0.013 | * |
| Italy | 1.21 | -4.72 | 7.51 | 0.659 |  |
| Malta | -6.25 | -13.30 | 1.38 | 0.099 |  |
| Slovenia | 1.09 | -9.90 | 13.43 | 0.833 |  |
| Spain | 2.13 | -10.37 | 16.36 | 0.752 |  |
| ***Eastern Europe*** |  |  |  |  |  |
| Bulgaria | 12.28 | -8.02 | 37.05 | 0.255 |  |
| Czech Republic | 0.26 | -3.53 | 4.19 | 0.882 |  |
| Poland | 14.70 | -13.74 | 52.52 | 0.346 |  |
| ***Africa*** |  |  |  |  |  |
| Uganda | -25.72 | -27.73 | -23.66 | 0.000 | * |

AAPC, annual percentage change; CI, confidence interval; * p values less than 0·05. NA, not available as it reported zero cases during the period and joinpoint regression could not be performed in such circumstances.

**Supplementary Figure 1:** Trend analysis of Ureter cancer by country

| **Asia** | | | |
| --- | --- | --- | --- |
|  |  |  | |
|  |  |  | |
|  |  |  | |
| **Oceania** | | |  |
|  |  |  |  |

| **Northern America** | | |
| --- | --- | --- |
|  |  |  |
| **Southern America** | | |
|  |  |  |
|  |  |  |

| **Northern Europe** | | |
| --- | --- | --- |
|  |  |  |
|  |  |  |
|  |  |  |

| **Western Europe** | | |
| --- | --- | --- |
|  |  |  |
|  |  |  |

| **Southern Europe** | | |
| --- | --- | --- |
|  |  |  |
|  |  |  |

| **Eastern Europe** | | | | |
| --- | --- | --- | --- | --- |
|  |  | |  | |
| **Africa** | | | | |
|  | |  | |  |

**Supplementary Figure 2.** Plots of Joinpoint regression for trend analysis

### Ureter - Male

| **Asia** | |
| --- | --- |
| 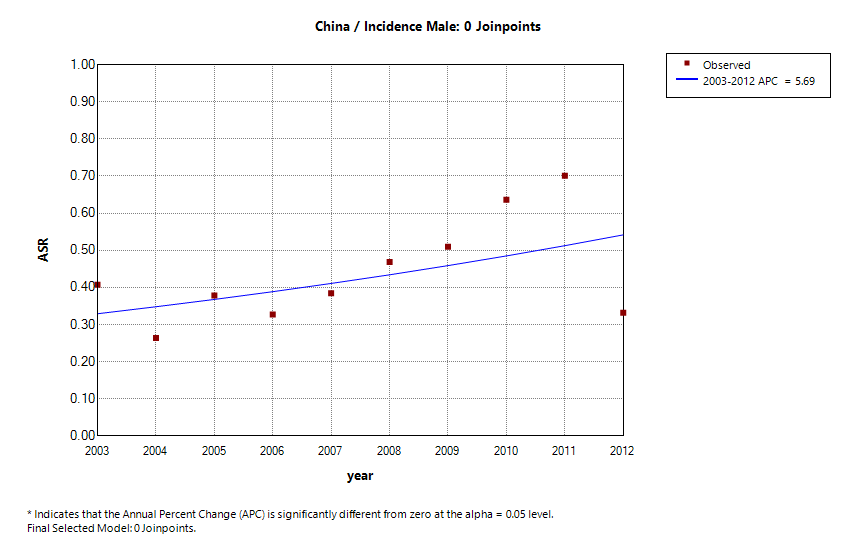 | 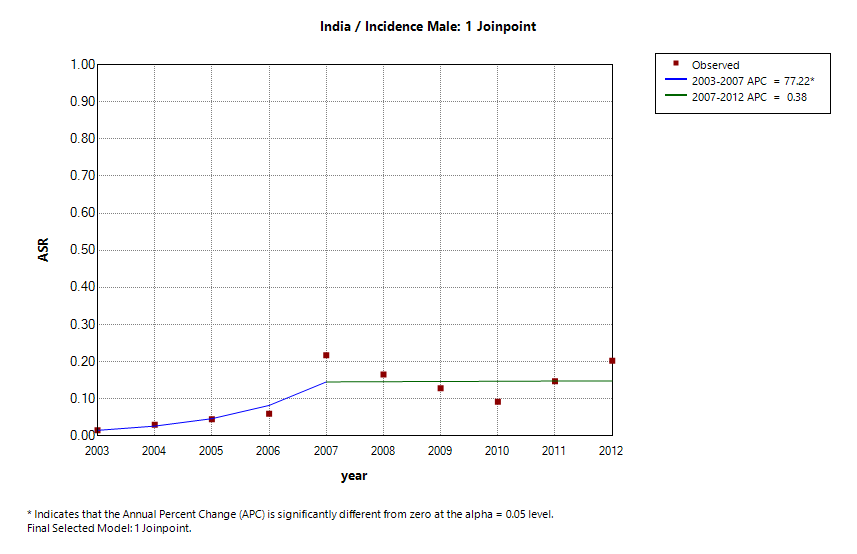 |
| 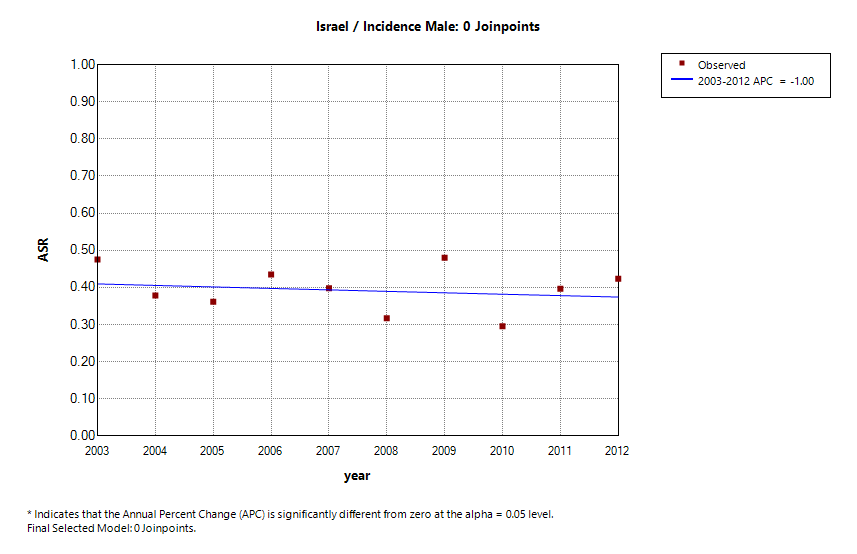 | 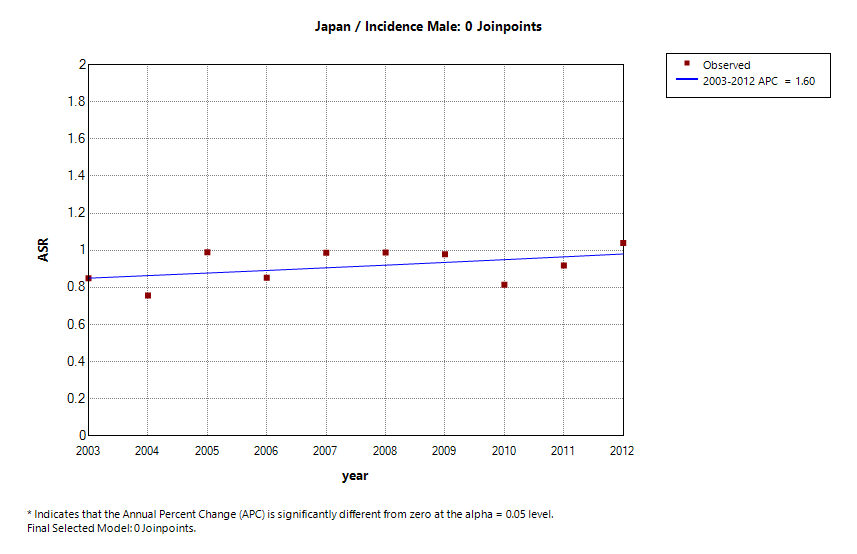 |
| 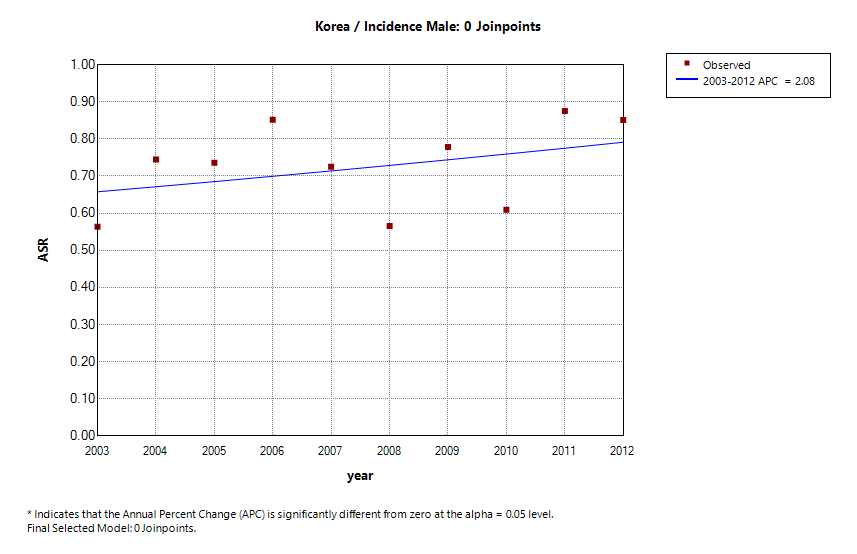 | 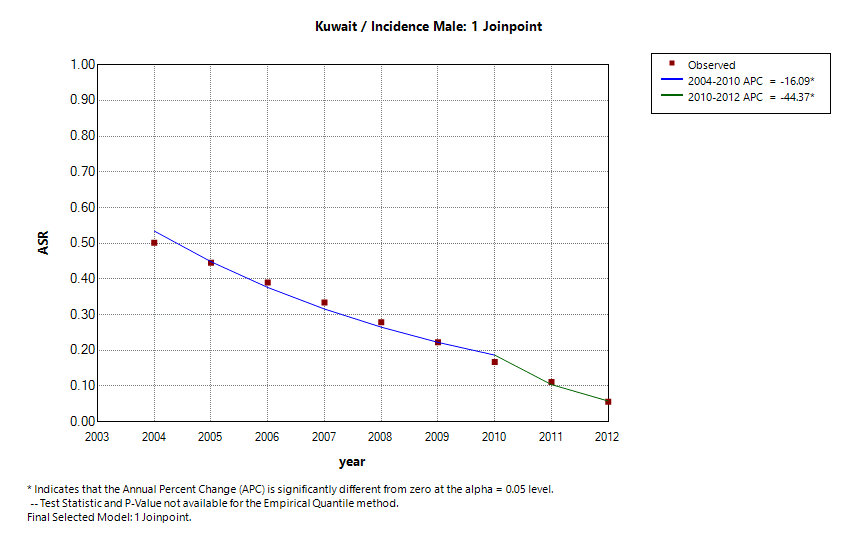  * 2003 data excluded as outliner |

| 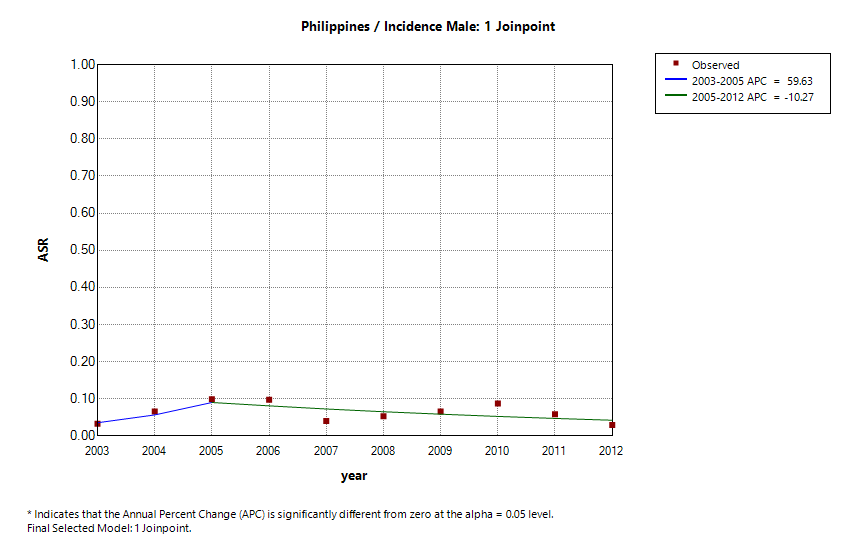 | 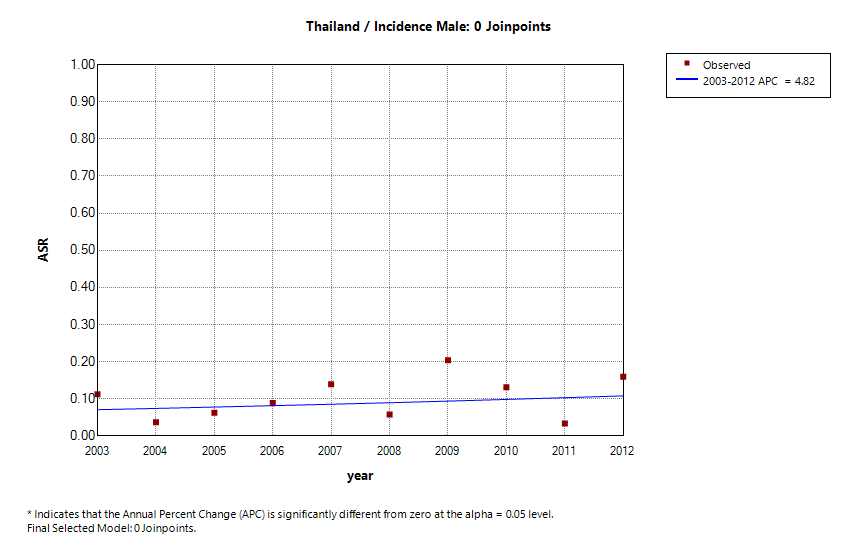 |
| --- | --- |
| 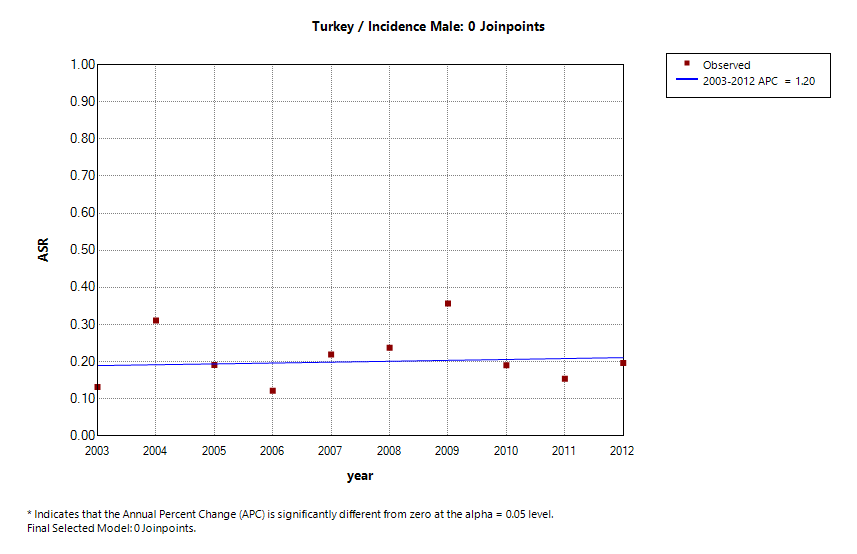 |  |
| **Oceania** | |
| 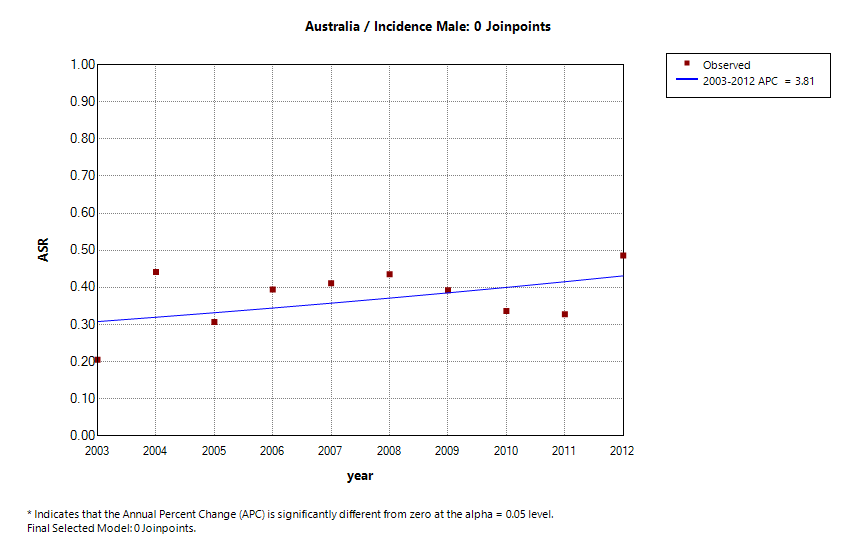 | 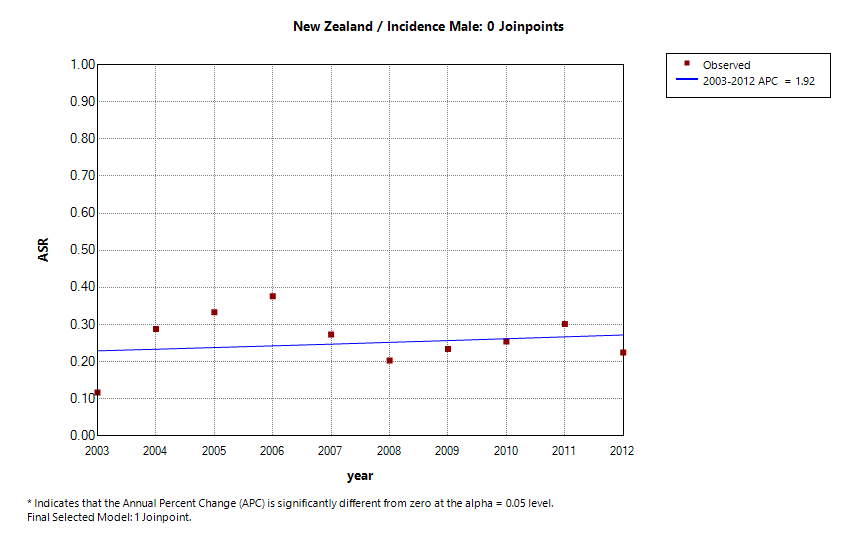 |

| **Northern America** | |
| --- | --- |
| 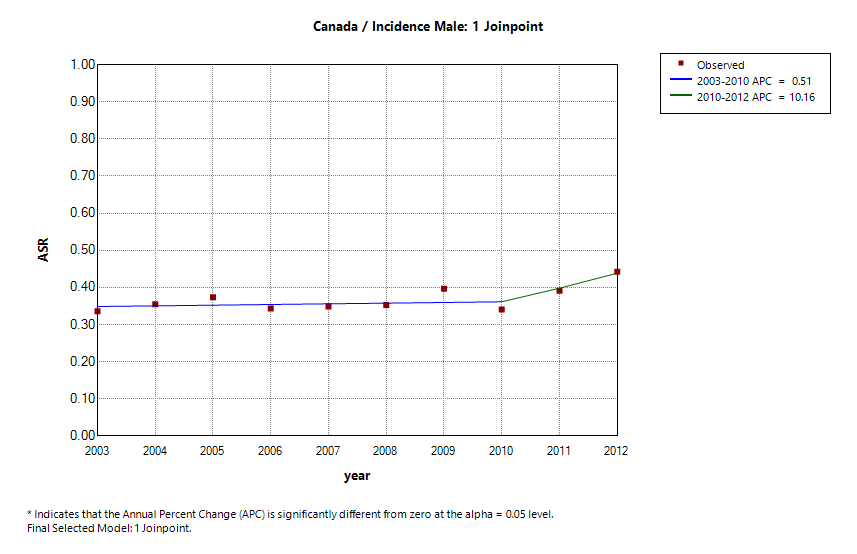 | 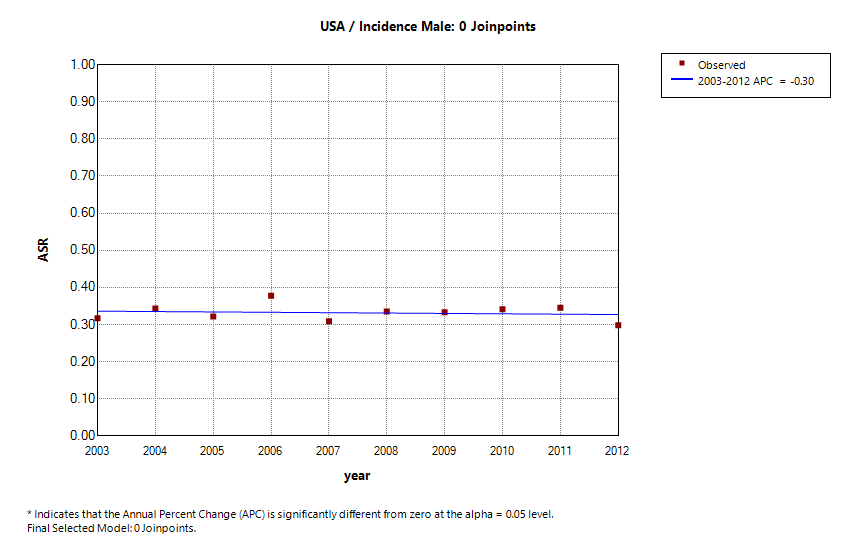 |
| **Southern America** | |
| 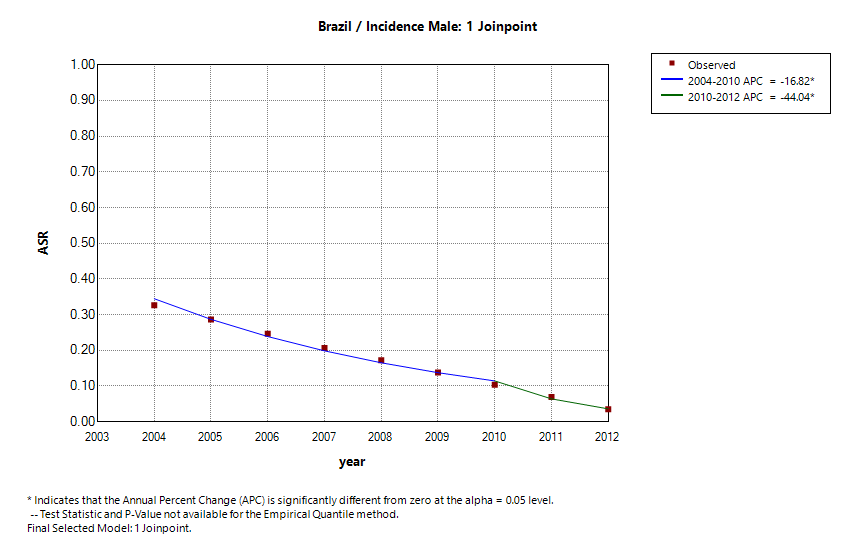  * 2003 data excluded as outliner | 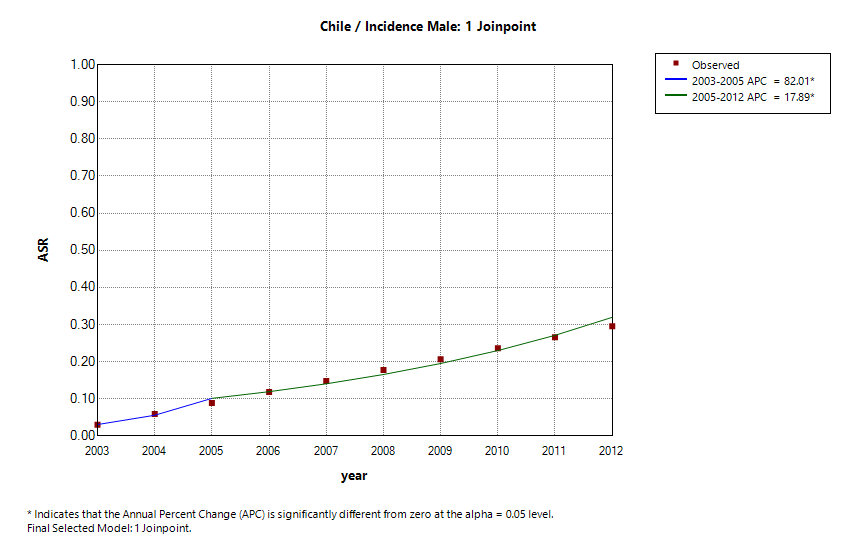 |
| 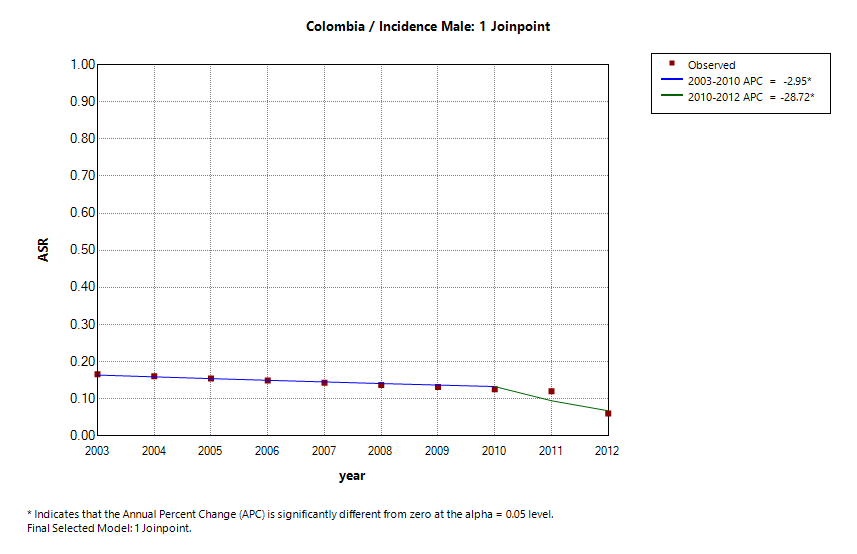 | 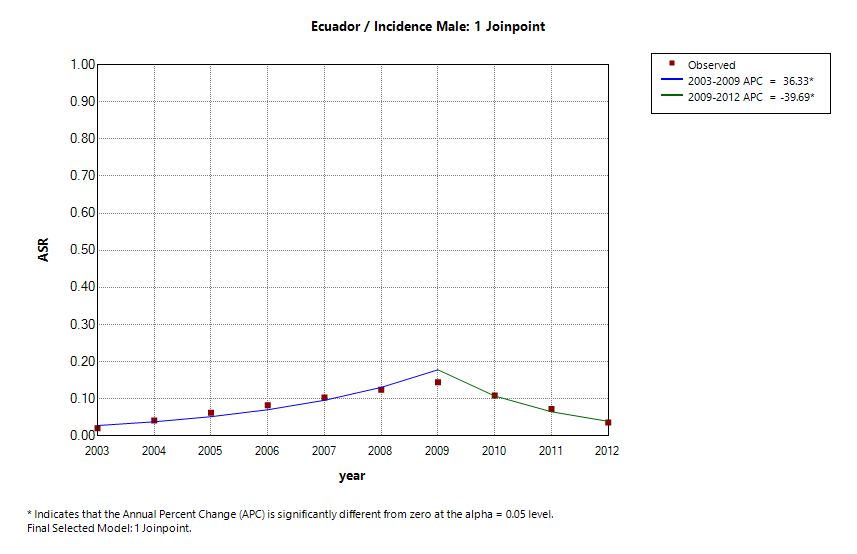 |

| **Northern Europe** | |
| --- | --- |
| 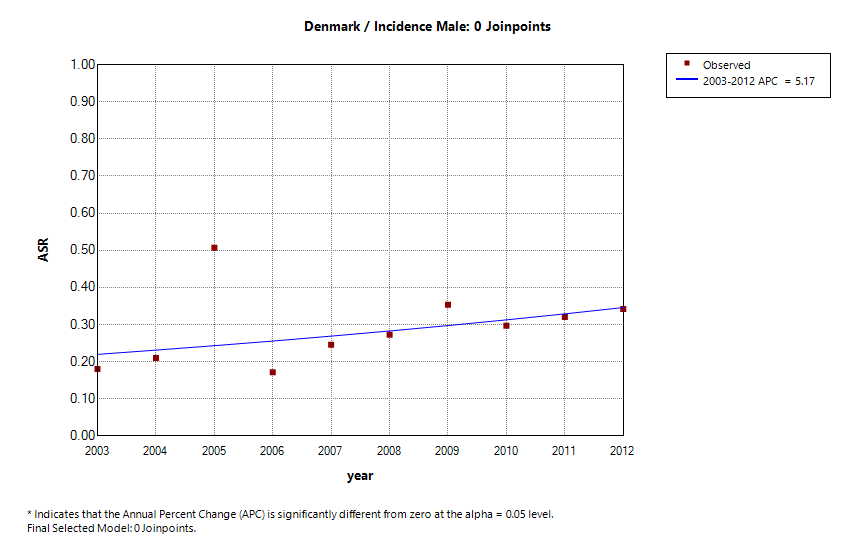 | 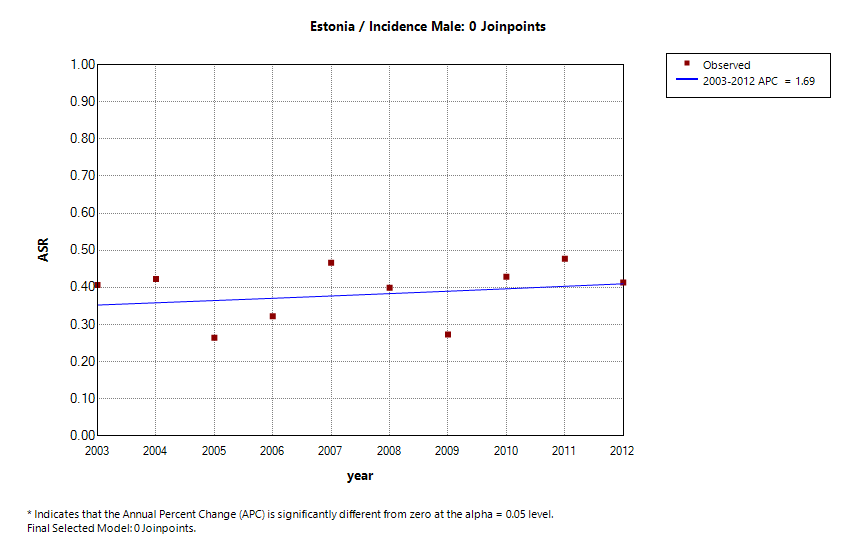 |
| 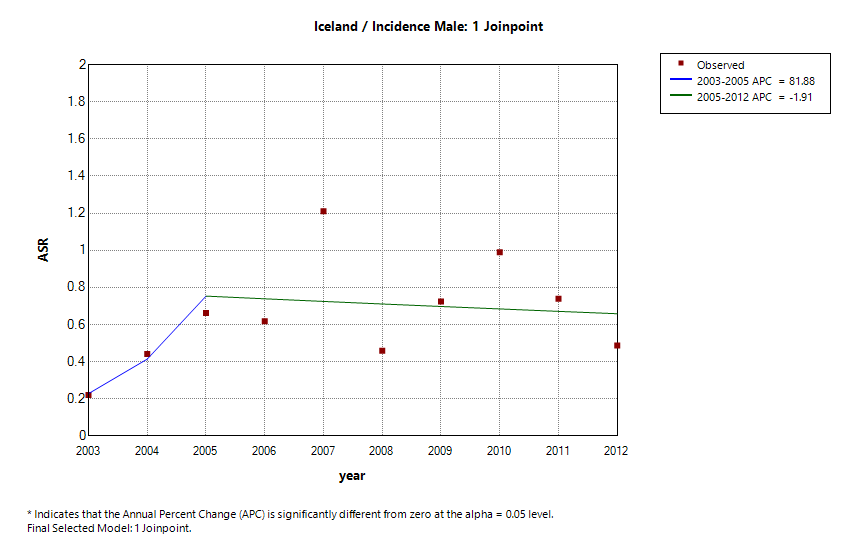 | 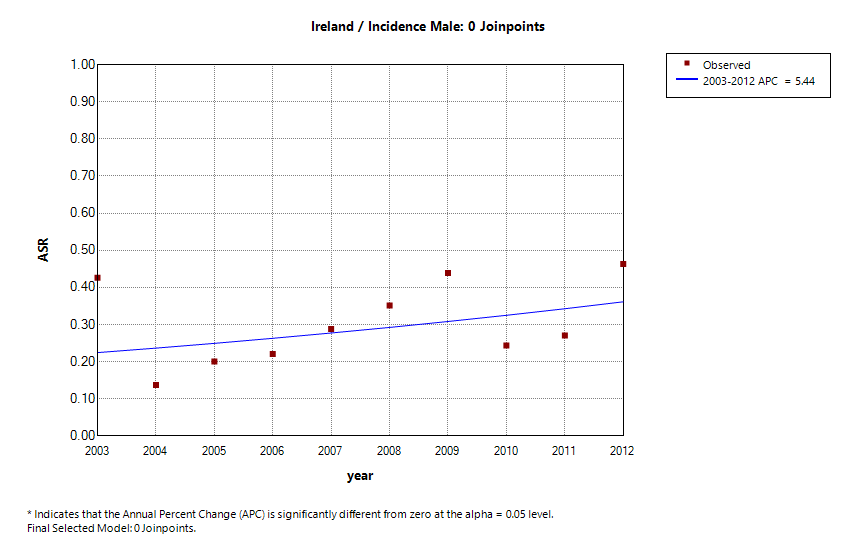 |
| 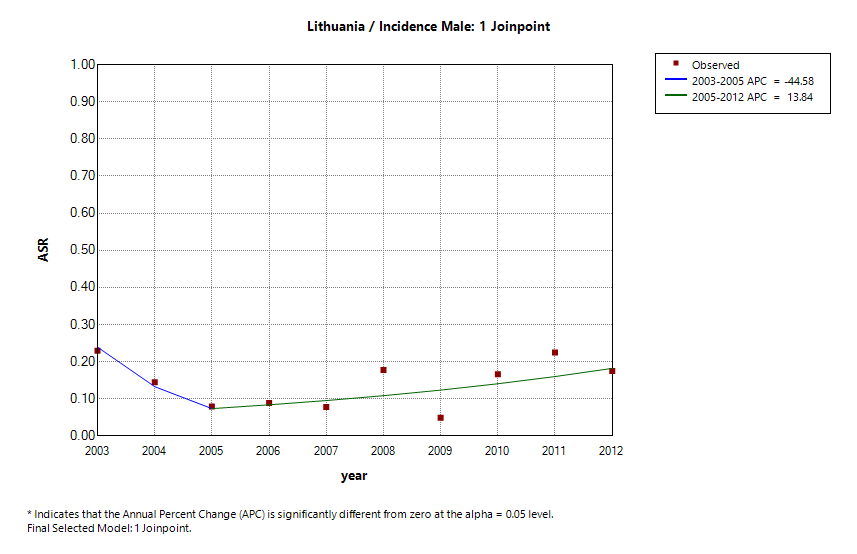 | 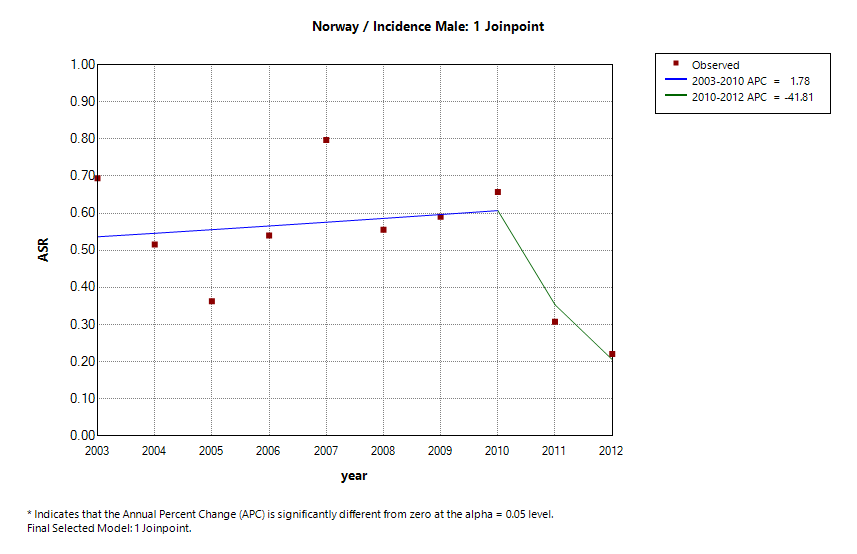 |

| 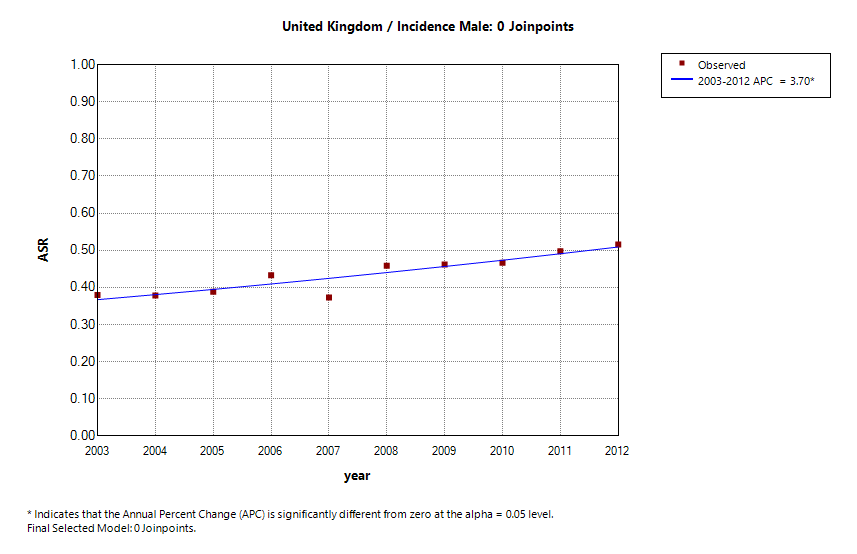 |  |
| --- | --- |
| **Western Europe** | |
| 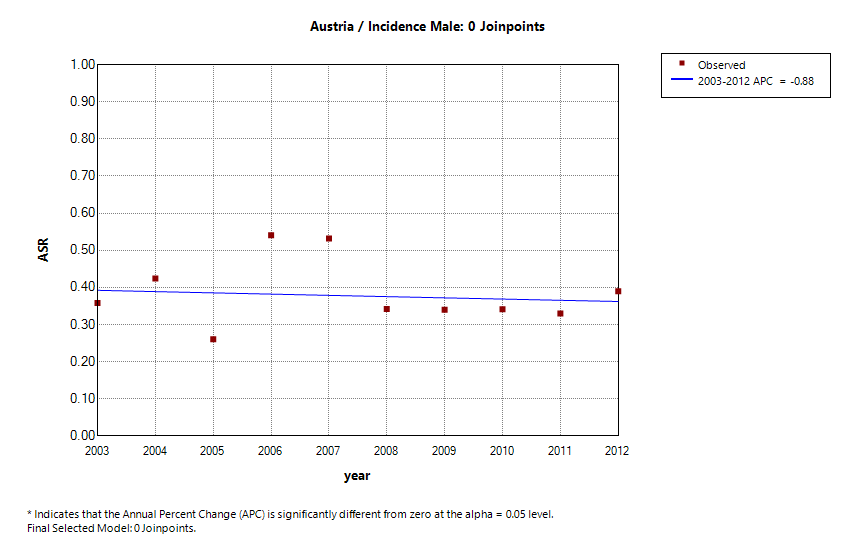 | 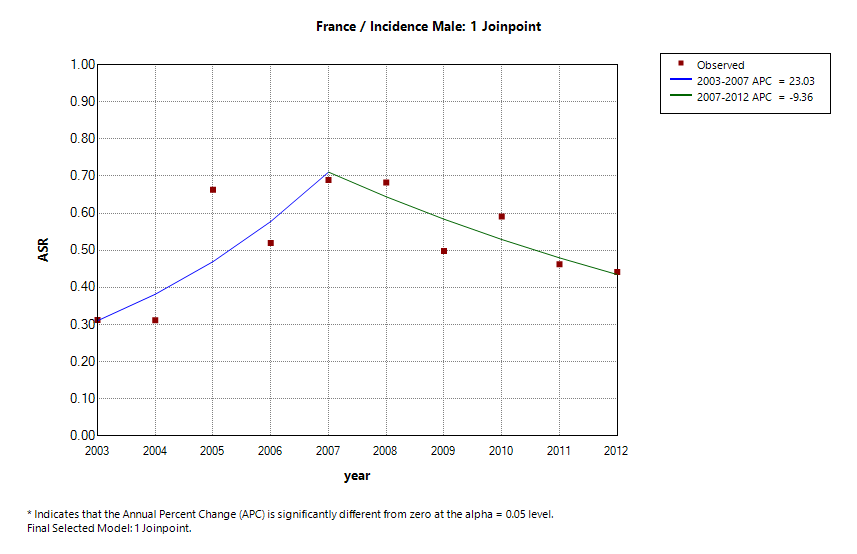 |
| 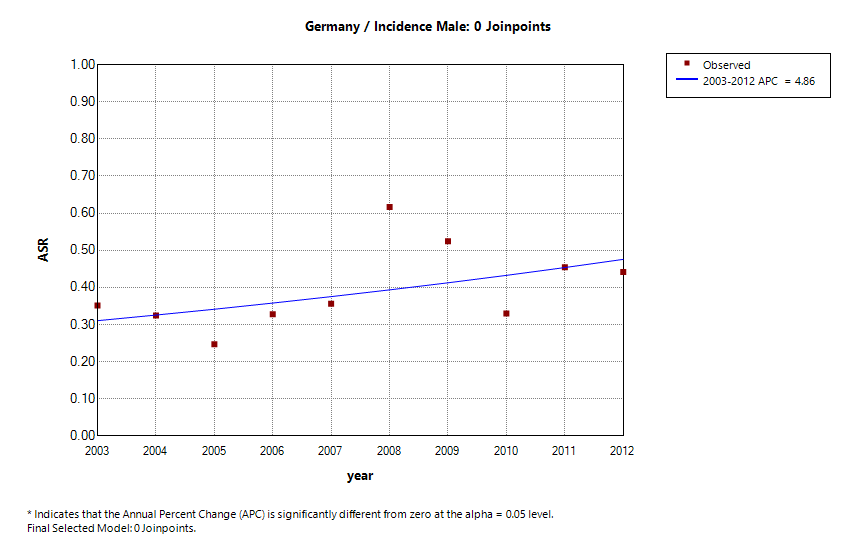 | 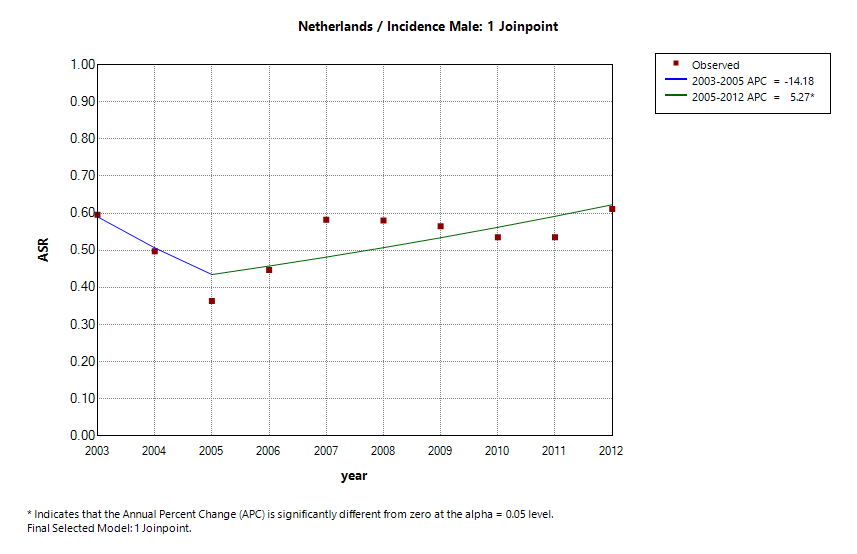 |

| 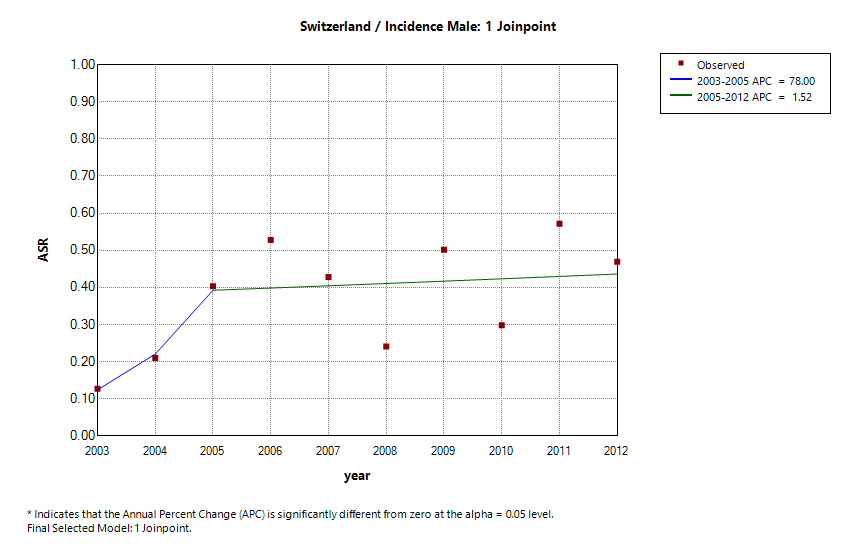 |  |
| --- | --- |
| **Southern Europe** | |
| 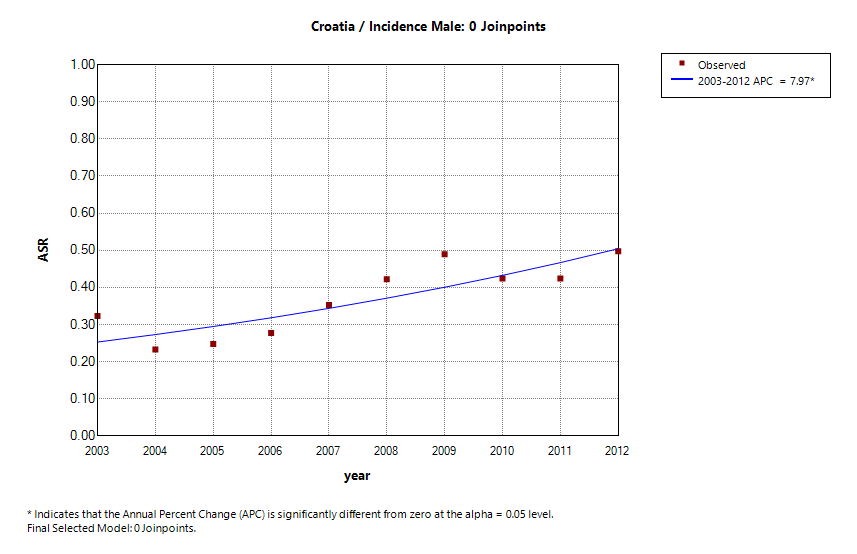 | 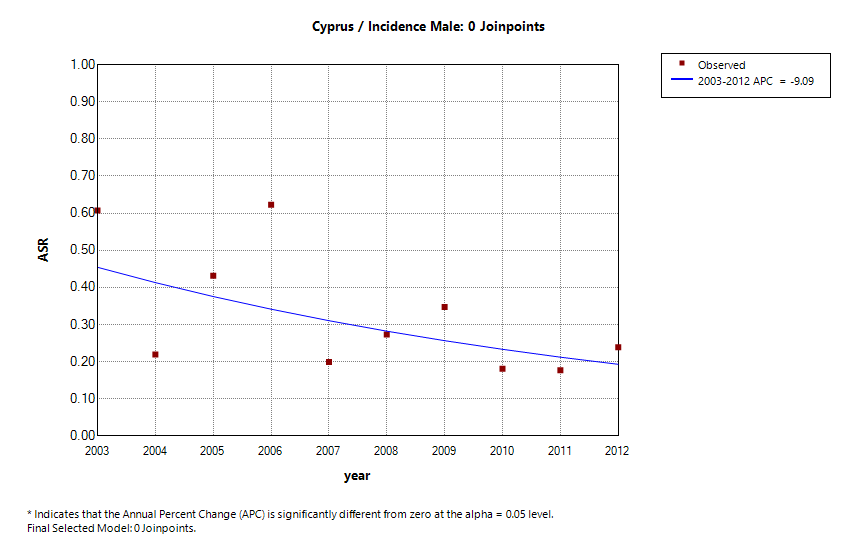 |
| 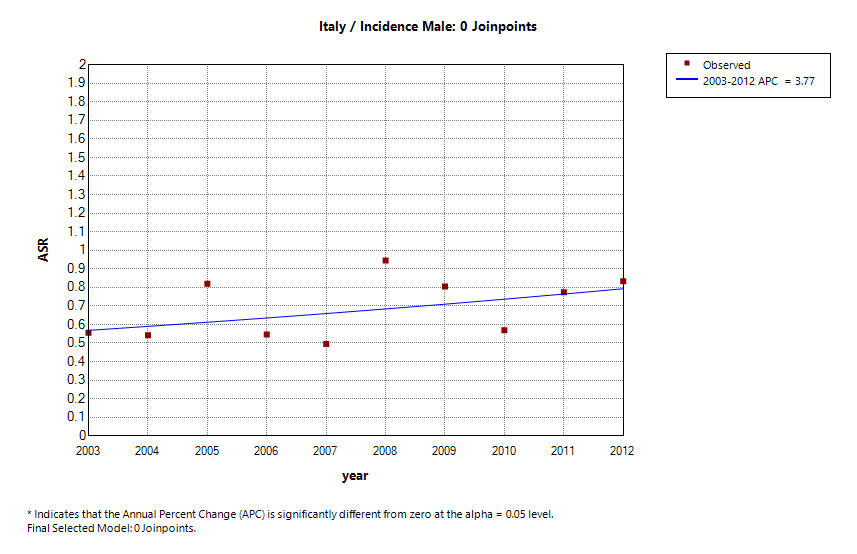 | 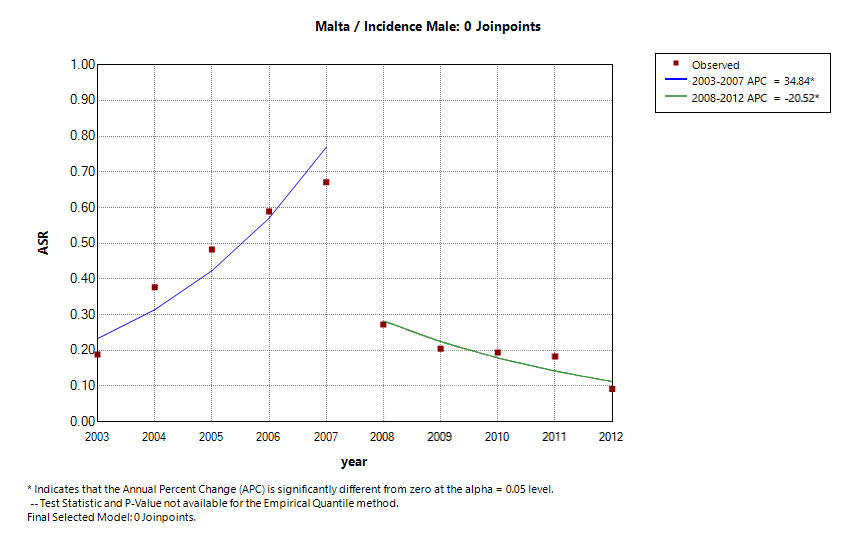 |

| 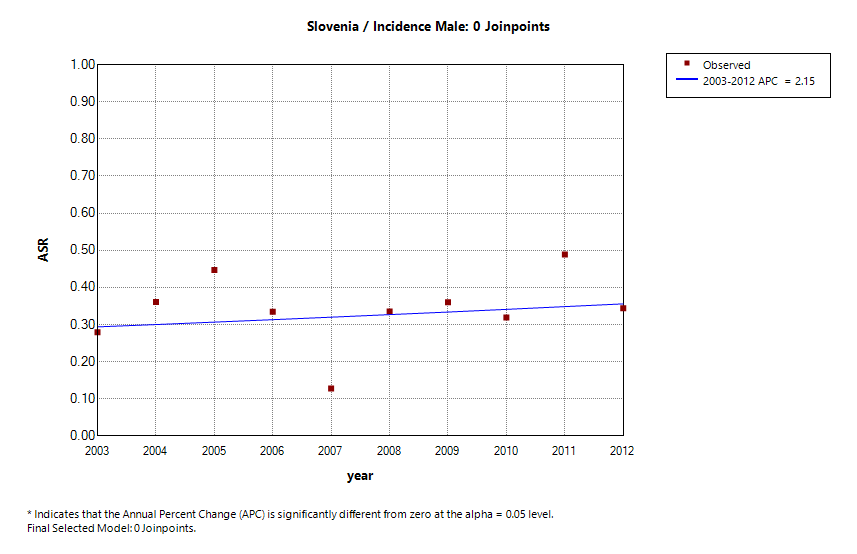 | 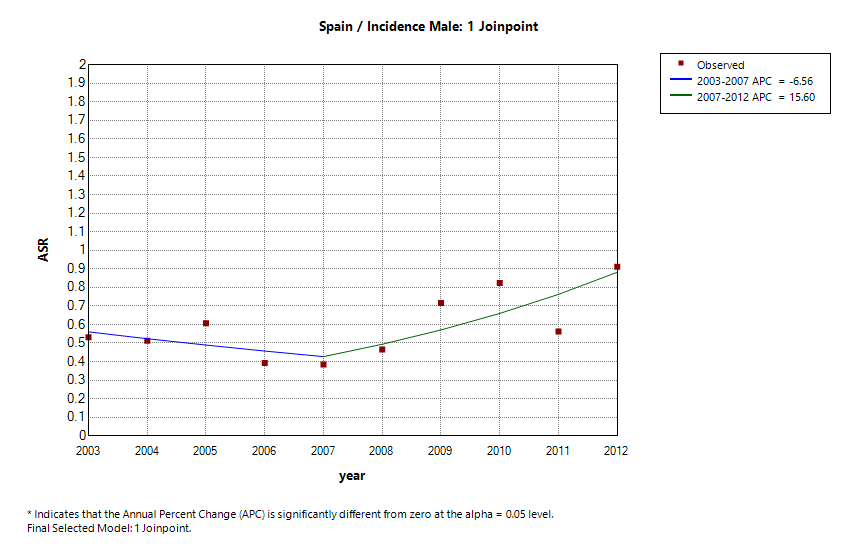 |
| --- | --- |
| **Eastern Europe** | |
| 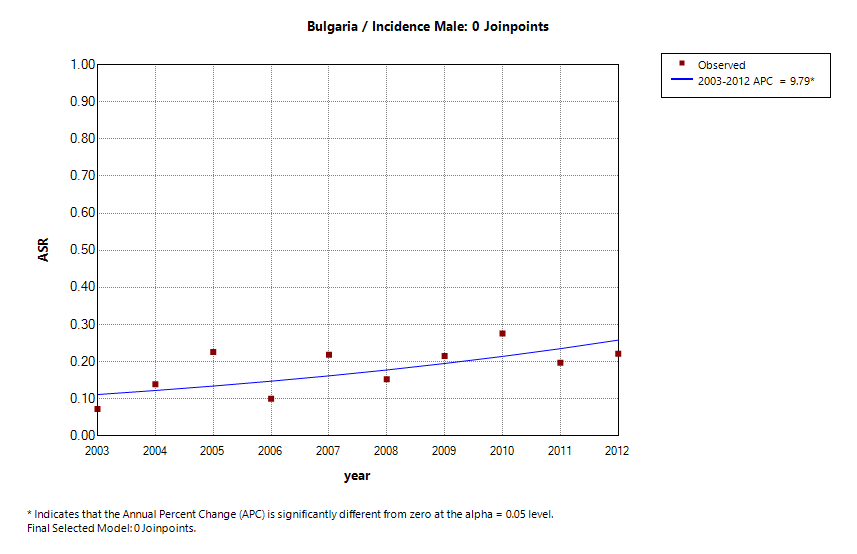 | 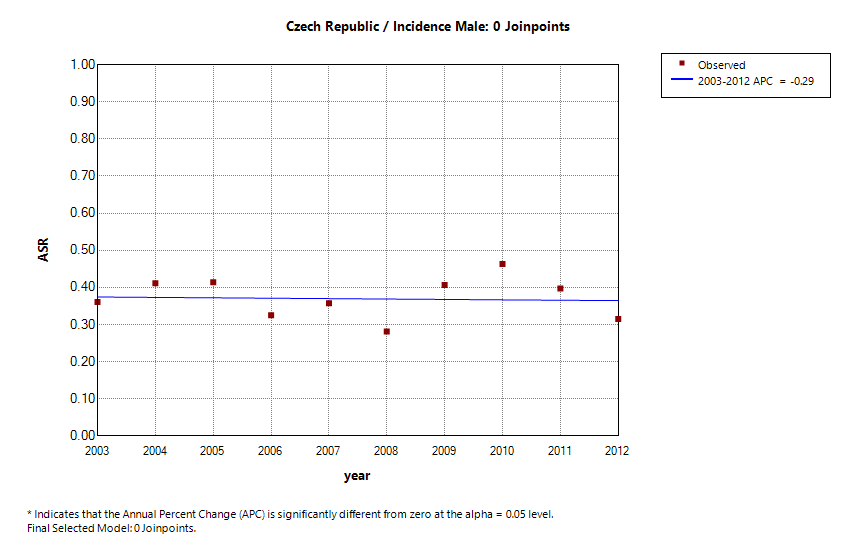 |
| 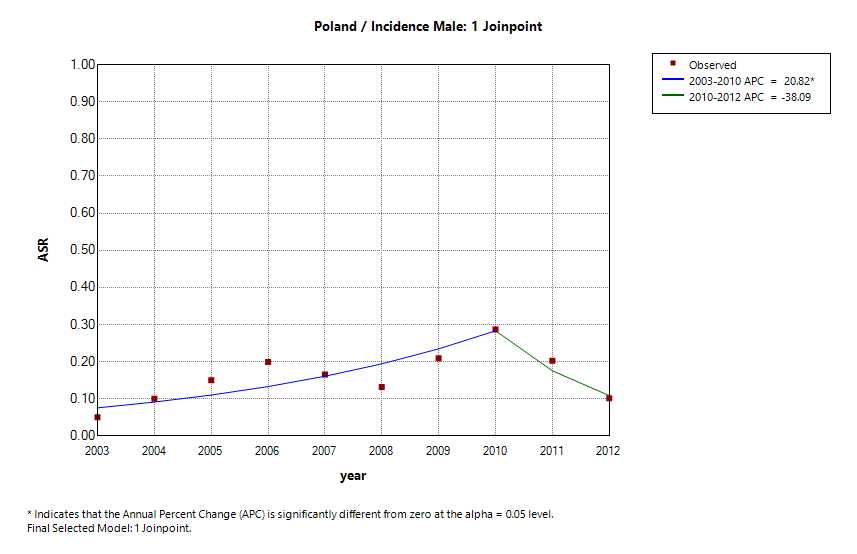 |  |

| **Africa** | |
| --- | --- |
| 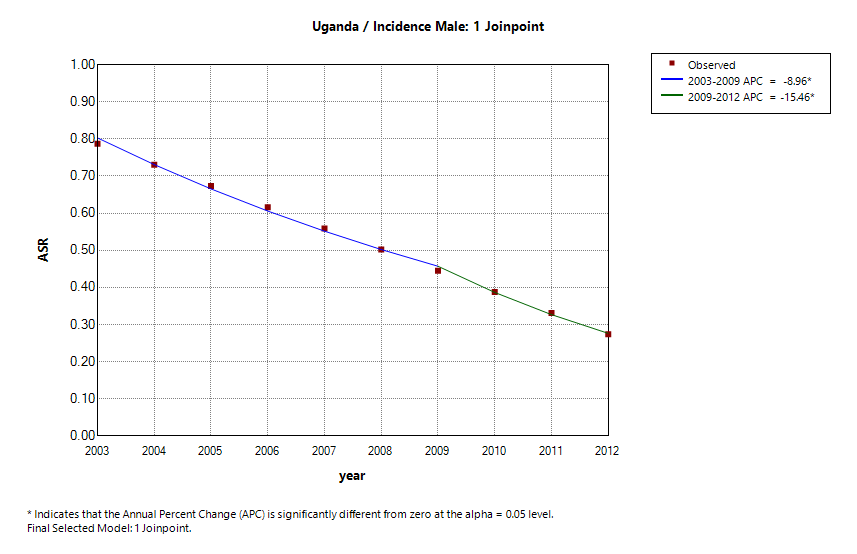 |  |

1. Ureter - Female

| **Asia** | |
| --- | --- |
| 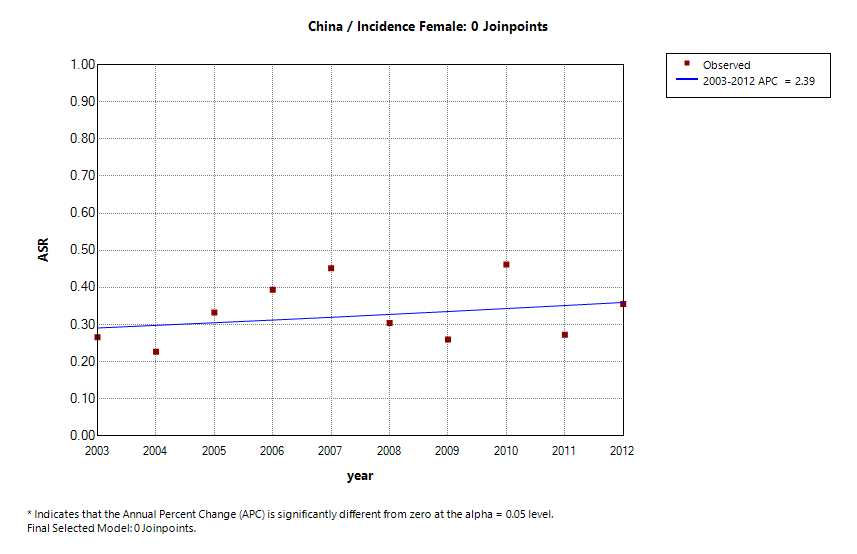 | 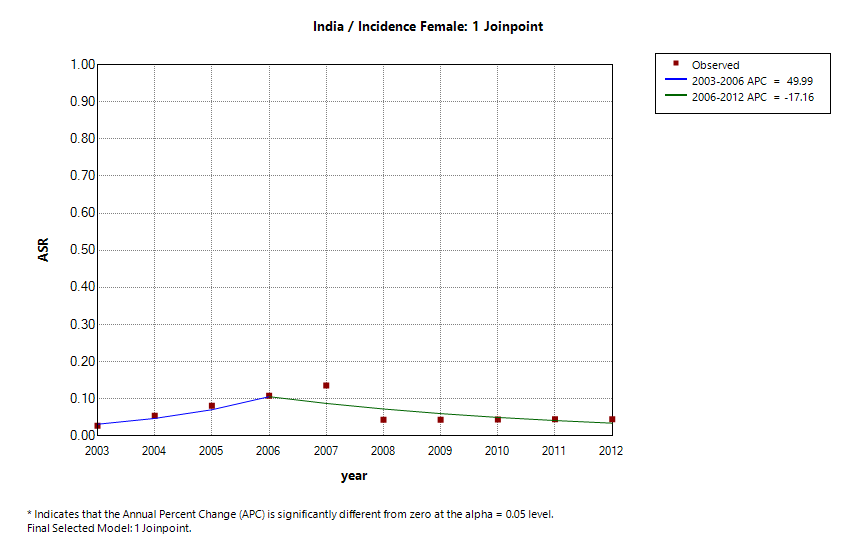 |
| 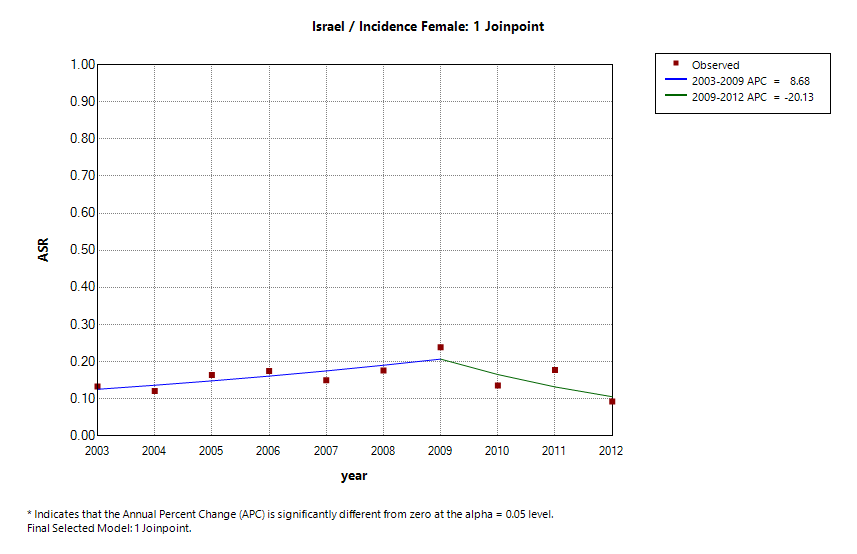 | 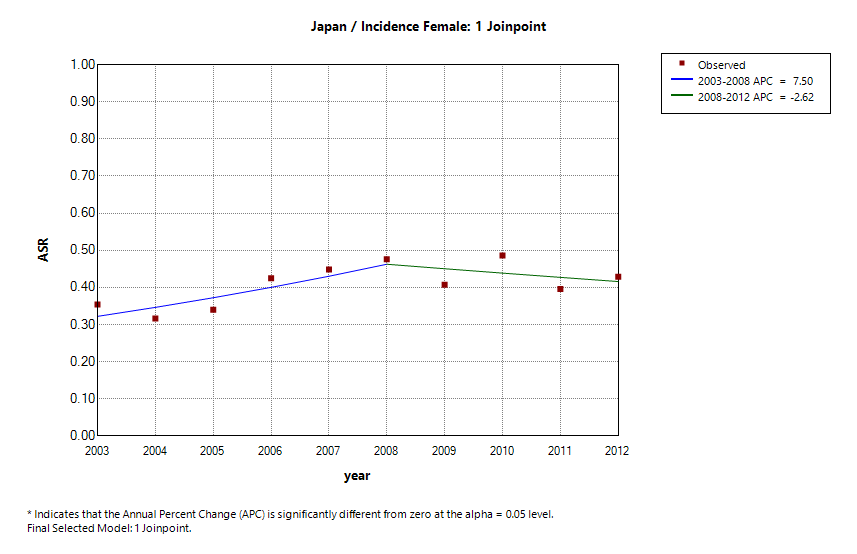 |
| 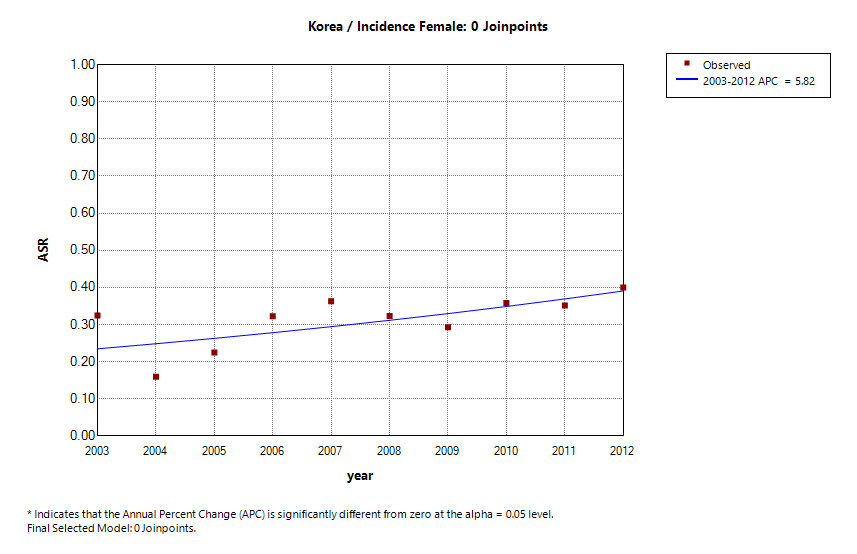 | 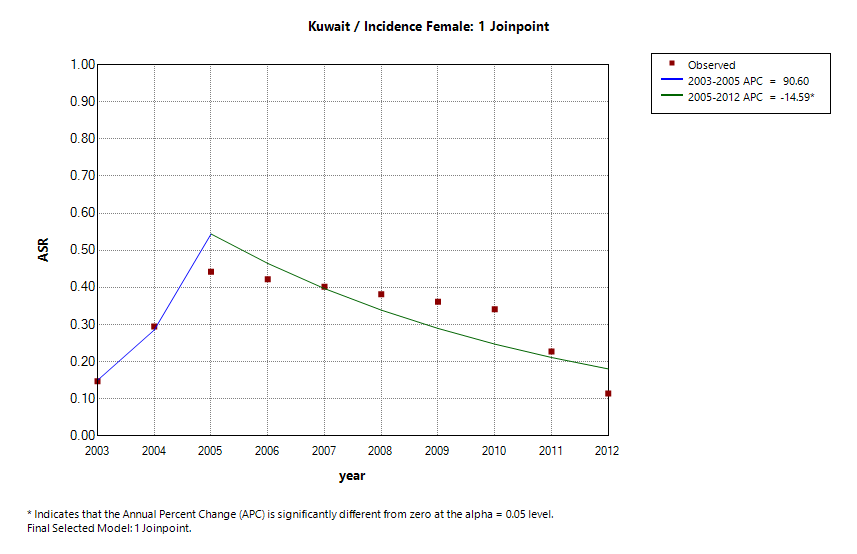 |

| 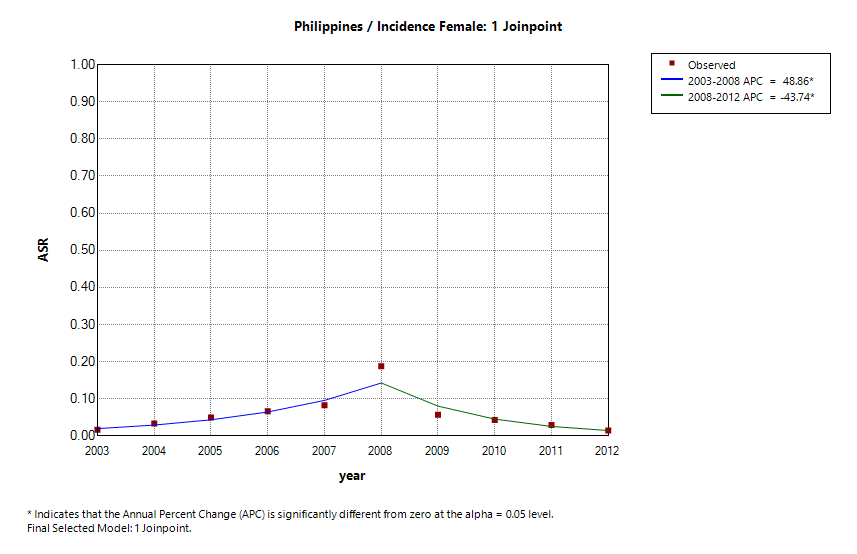 | 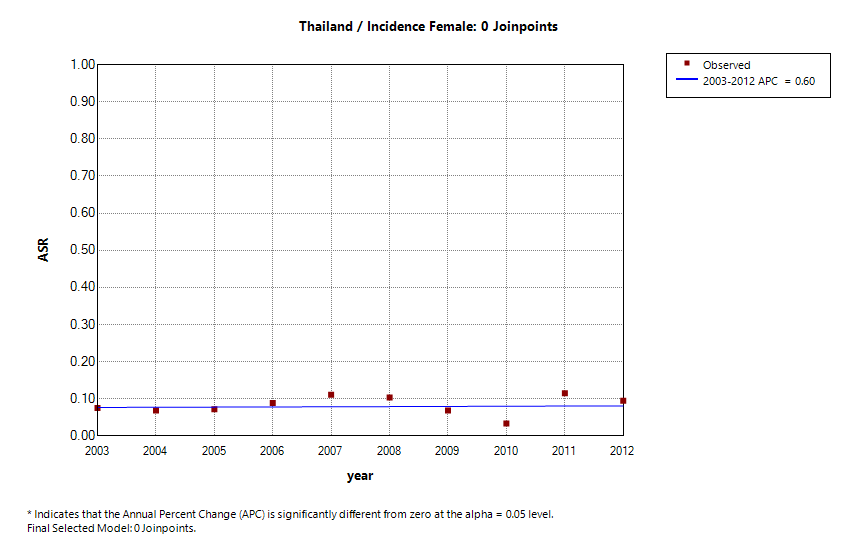 |
| --- | --- |
| 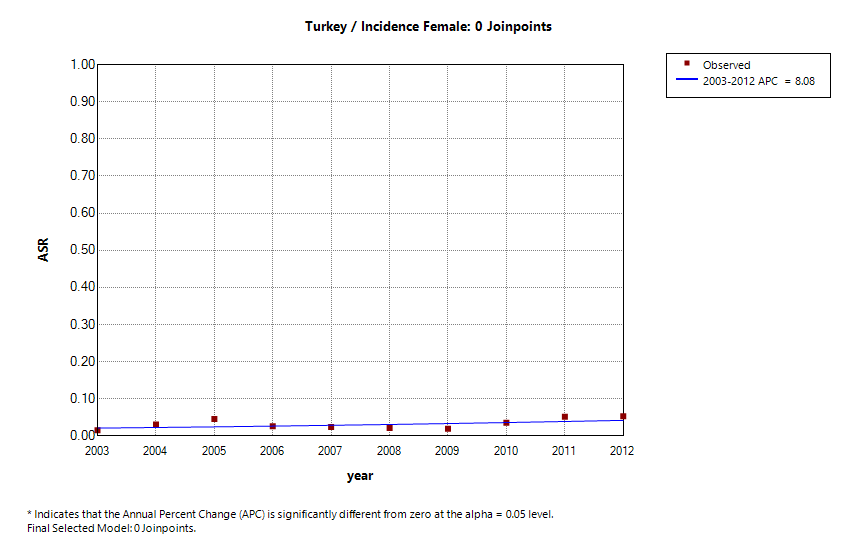 |  |
| **Oceania** | |
| 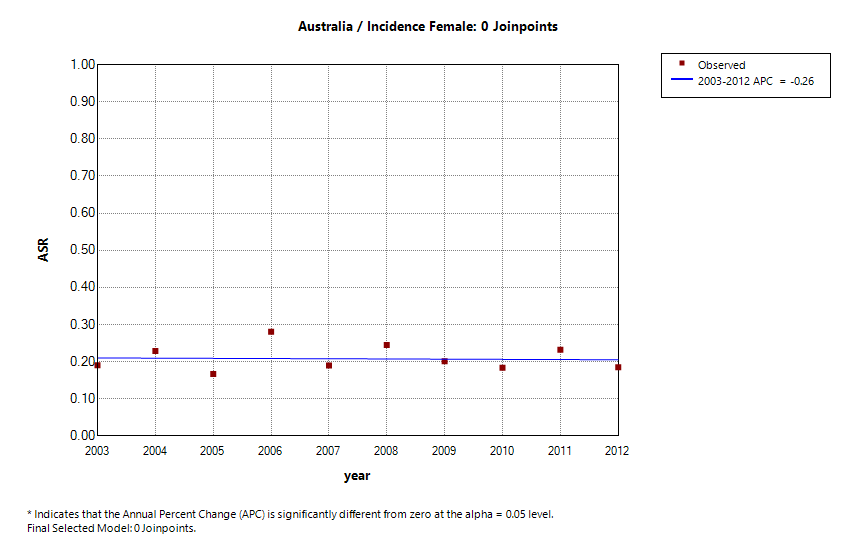 | 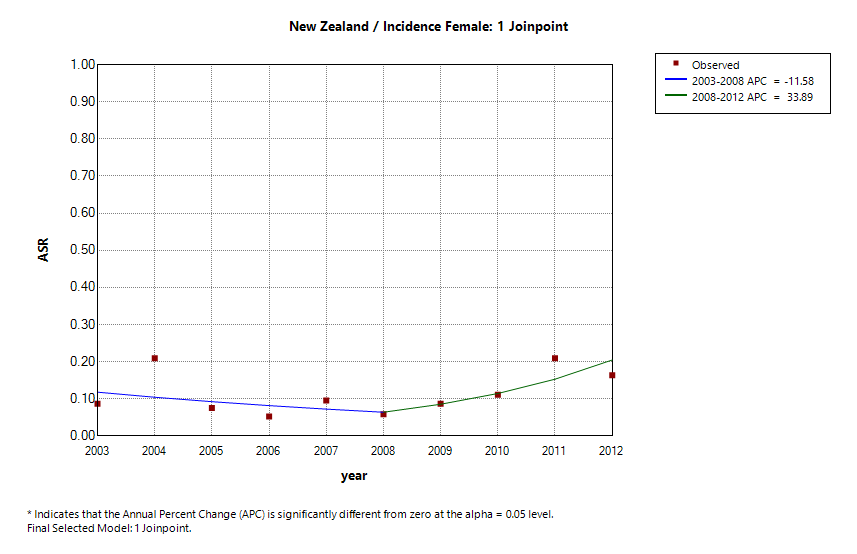 |

| **Northern America** | |
| --- | --- |
| 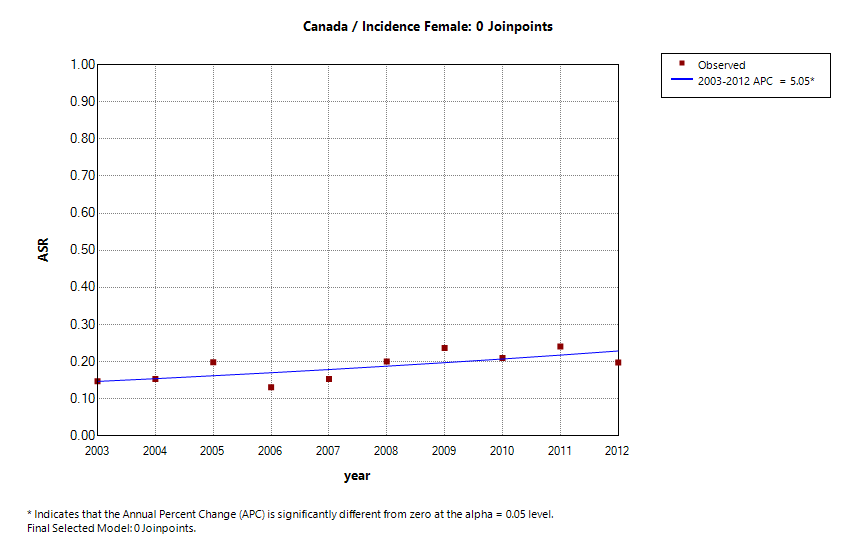 | 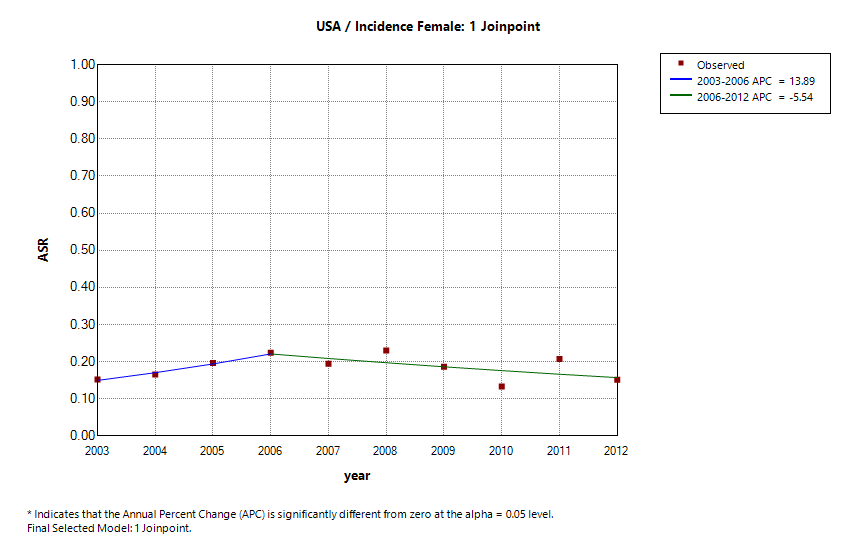 |
| **Southern America** | |
| 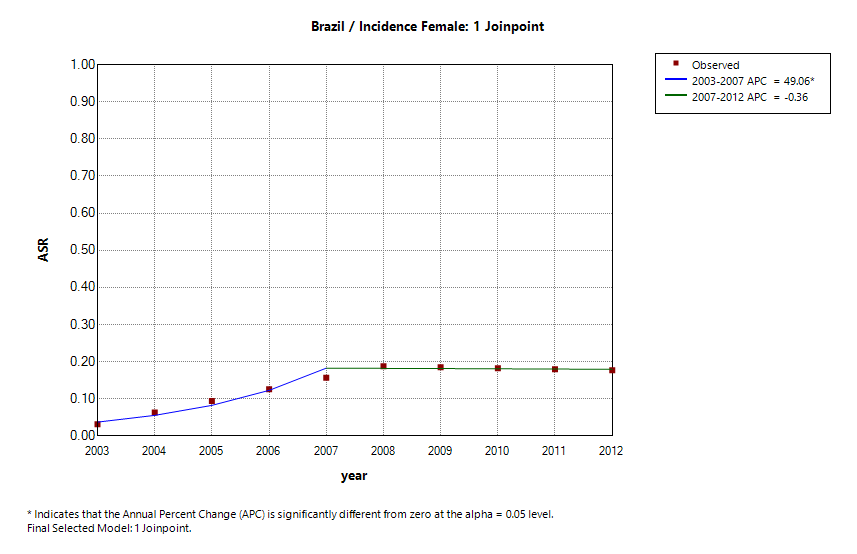 | 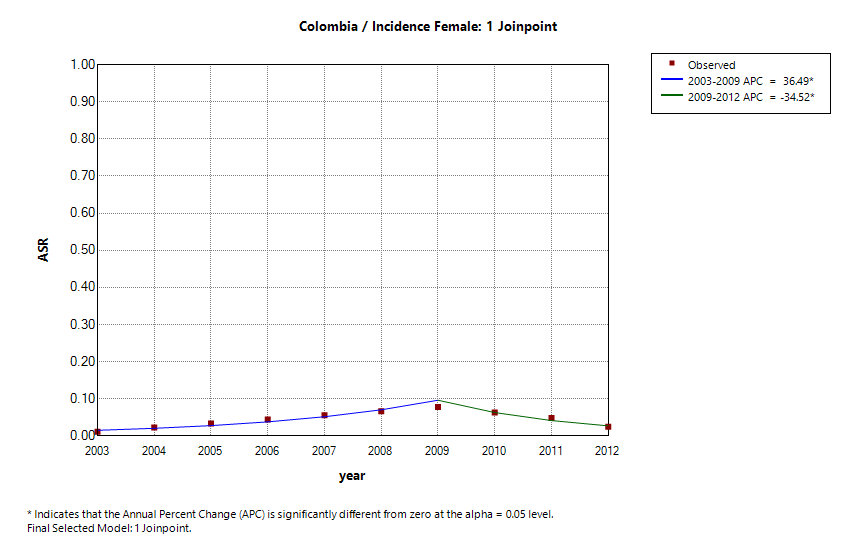 |
| 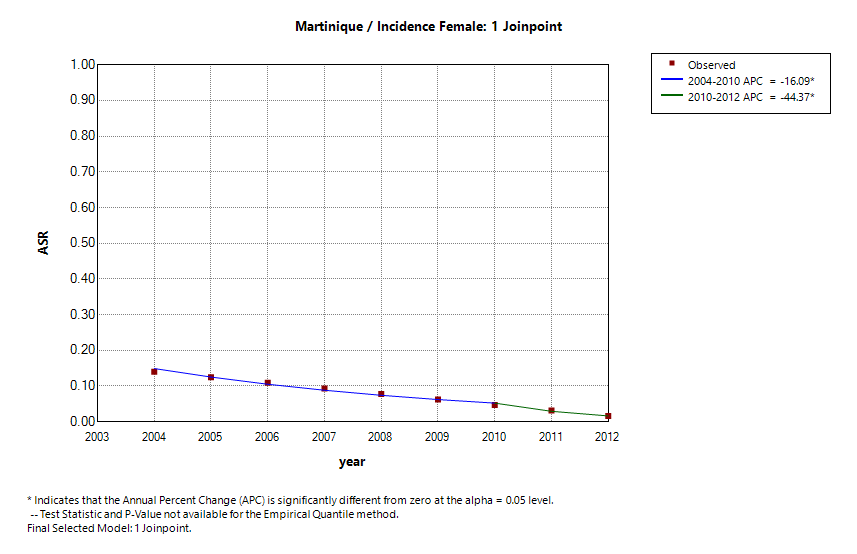  * 2003 data excluded as outliner |  |

| **Northern Europe** | |
| --- | --- |
| 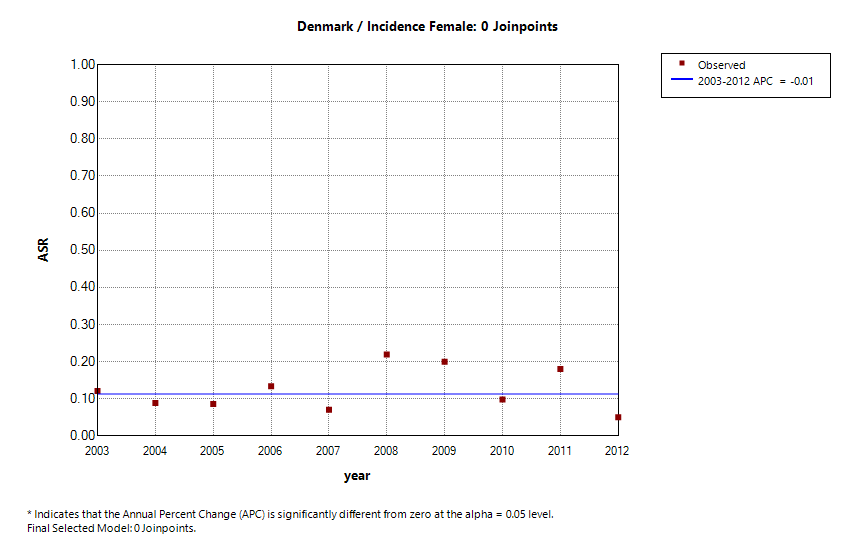 | 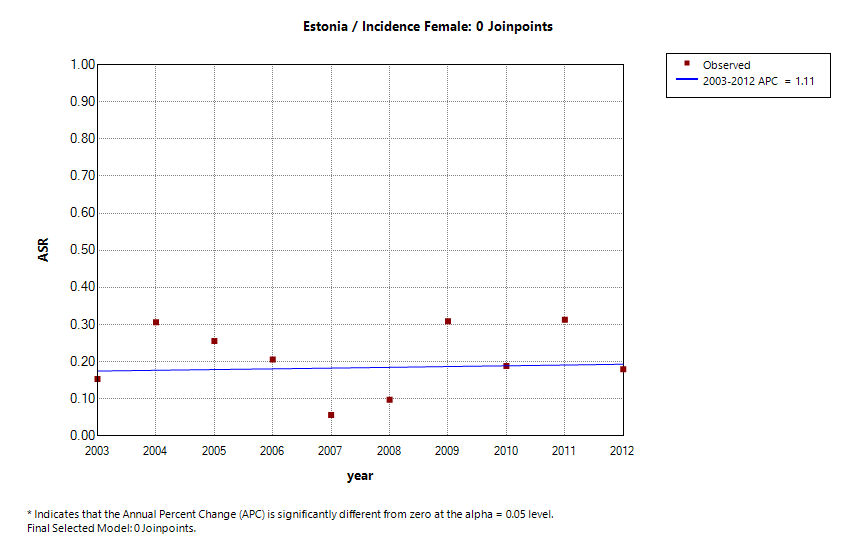 |
| 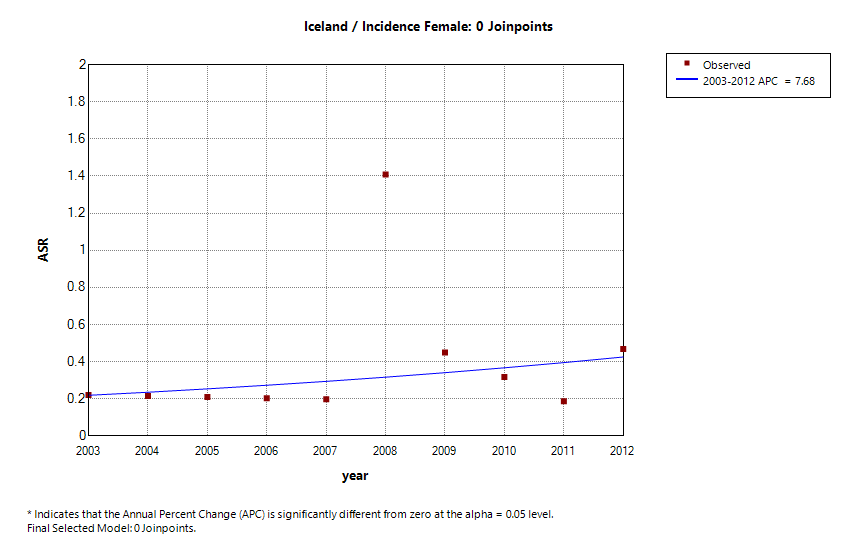 | 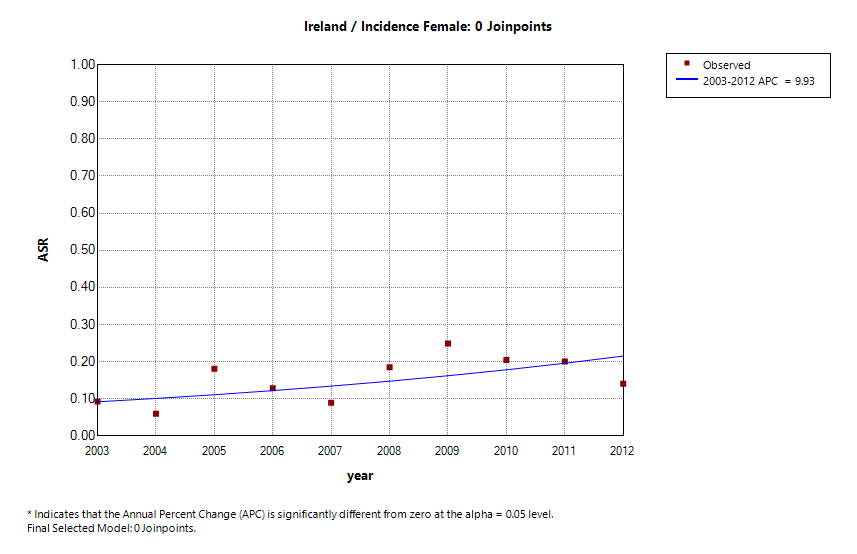 |
| 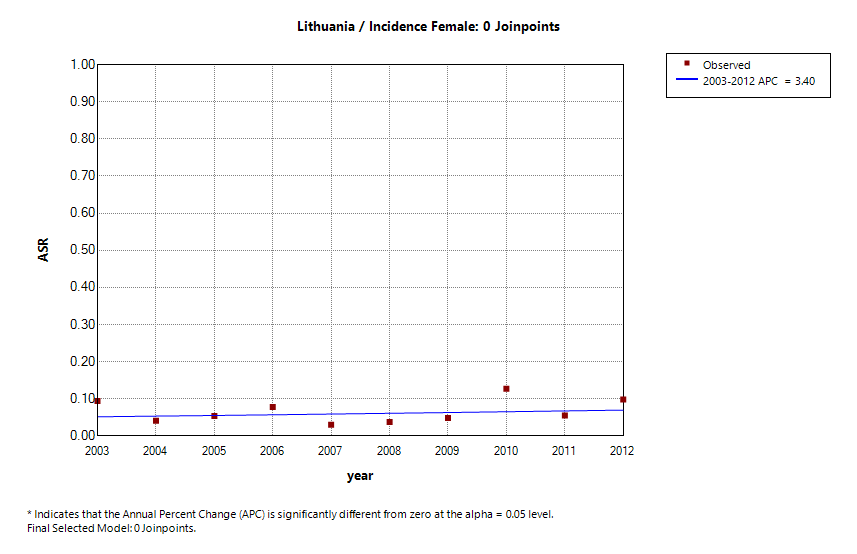 | 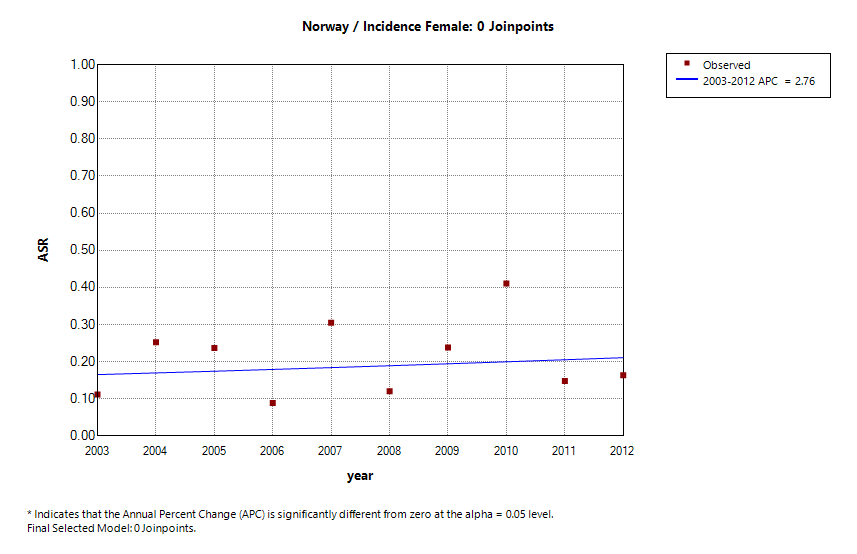 |

| 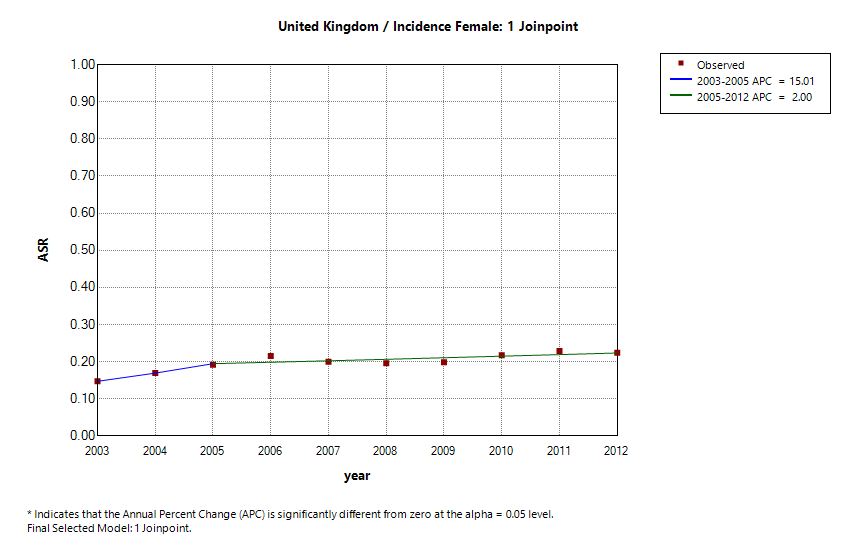 |  |
| --- | --- |
| **Western Europe** | |
| 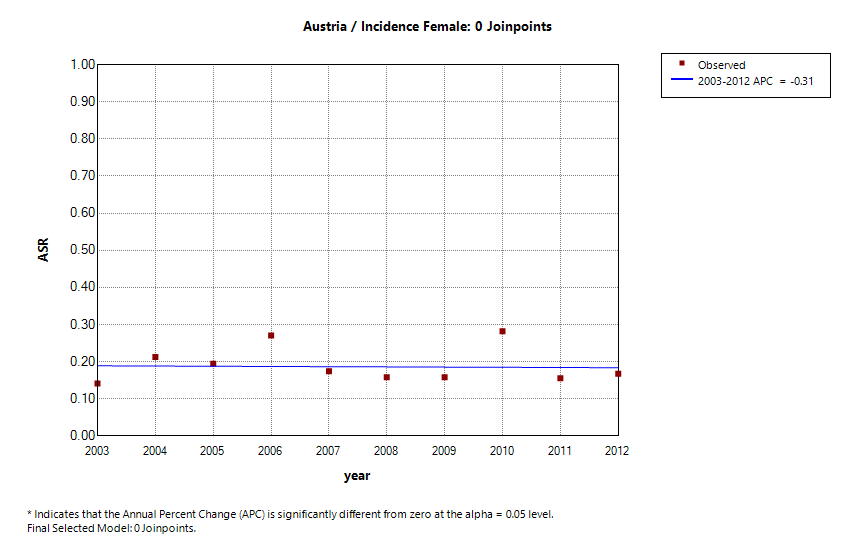 | 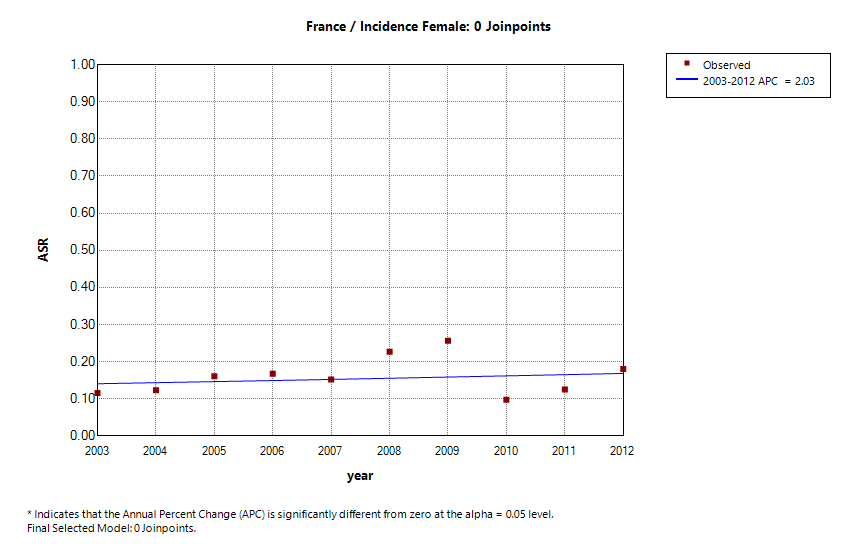 |
| 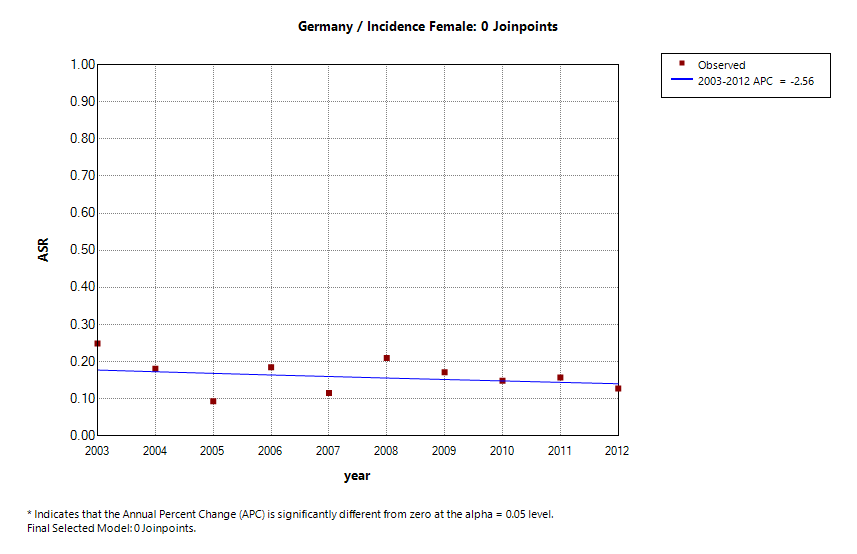 | 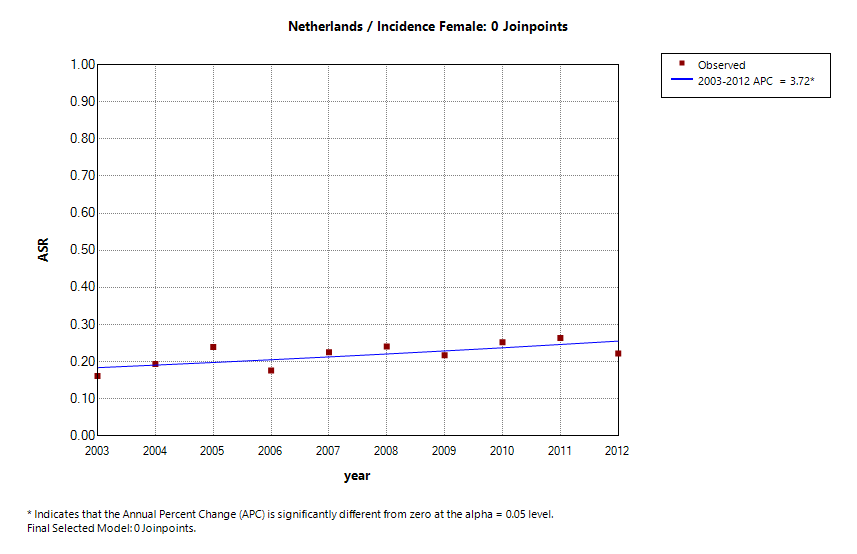 |

| 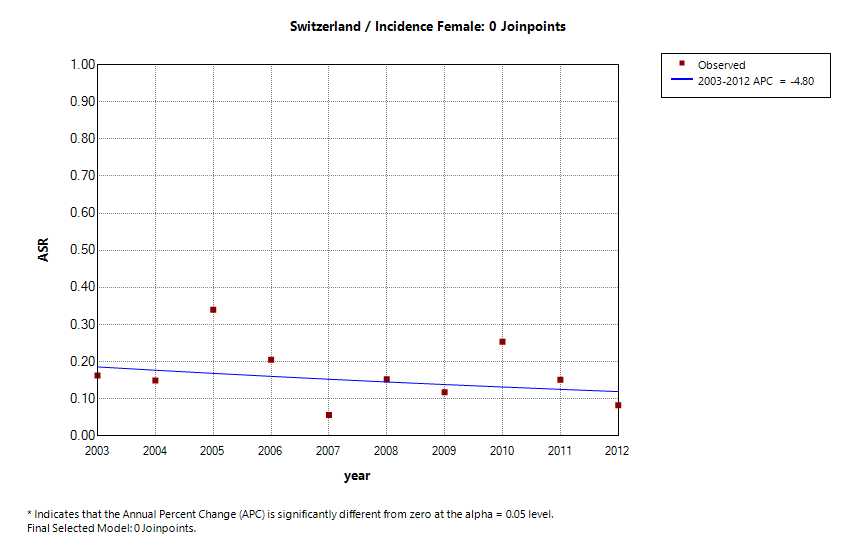 |  |
| --- | --- |
| **Southern Europe** | |
| 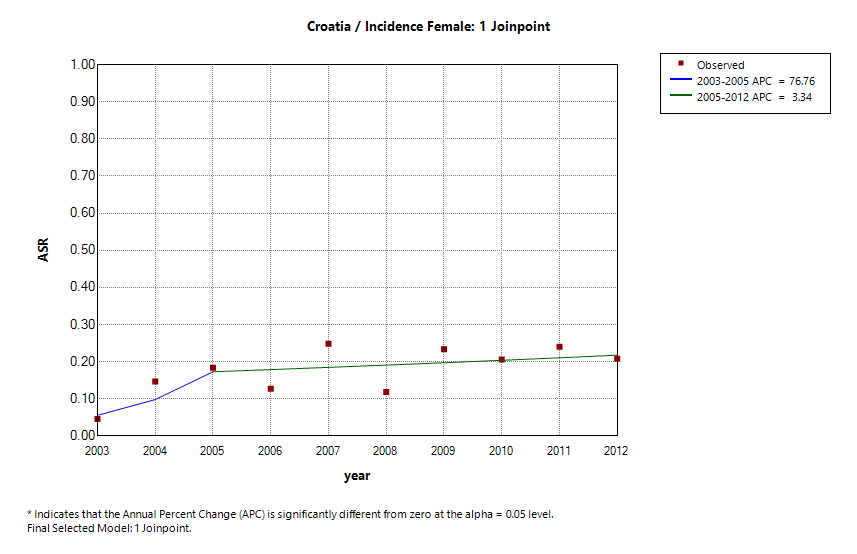 | 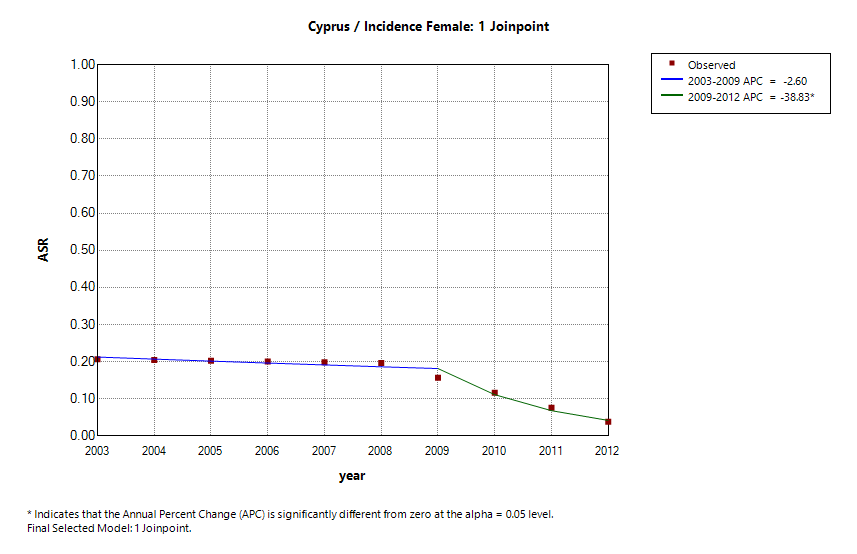 |
| 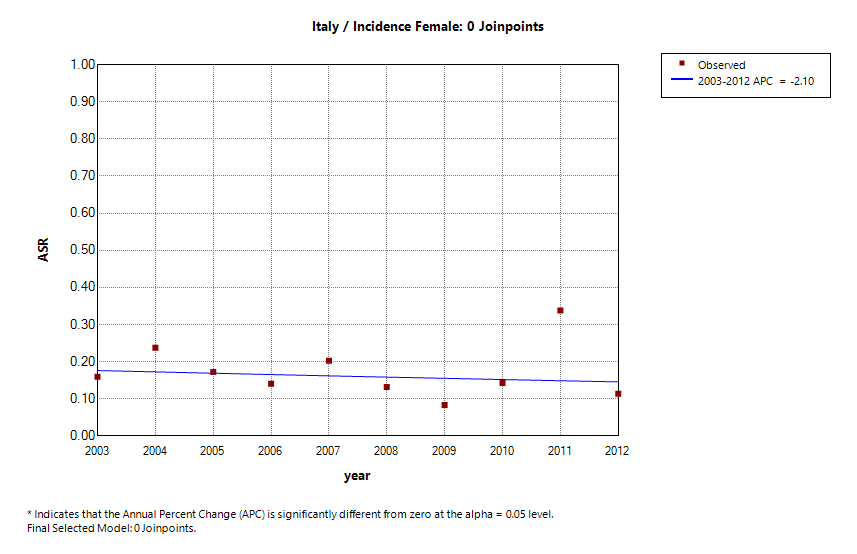 | 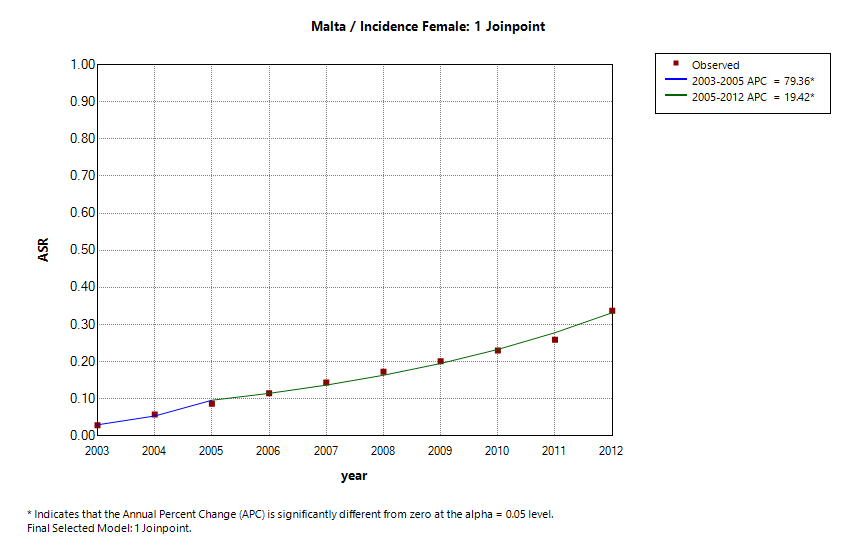 |

| 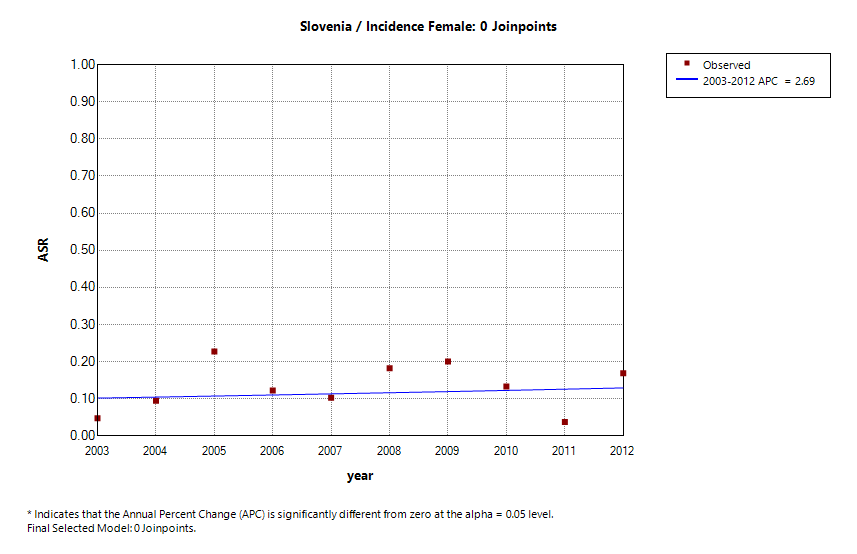 | 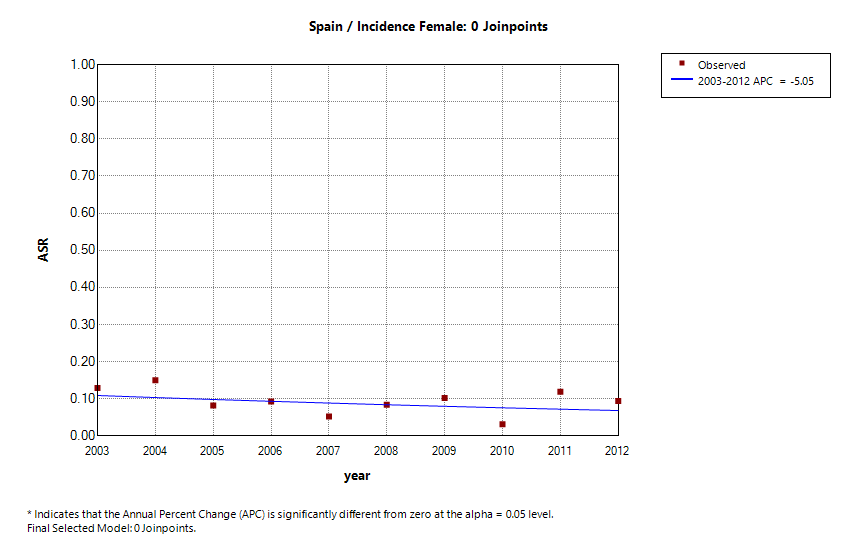 |
| --- | --- |
| **Eastern Europe** | |
| 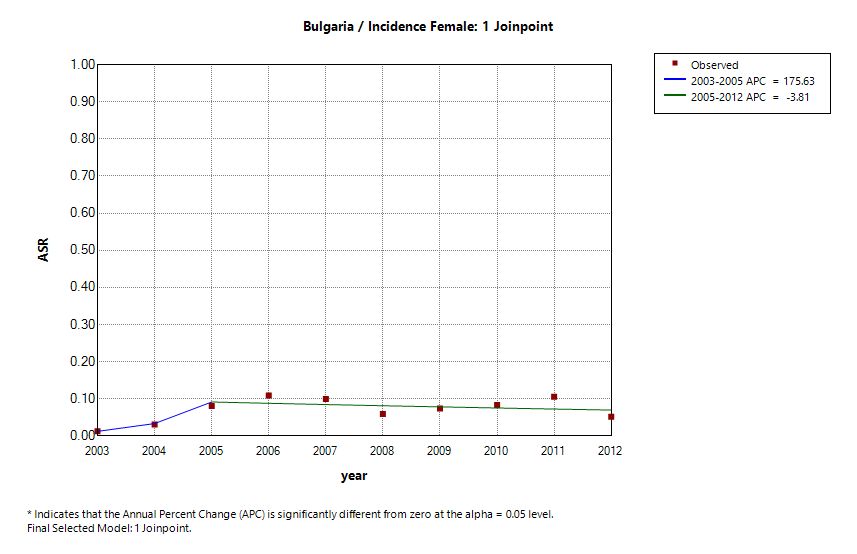 | 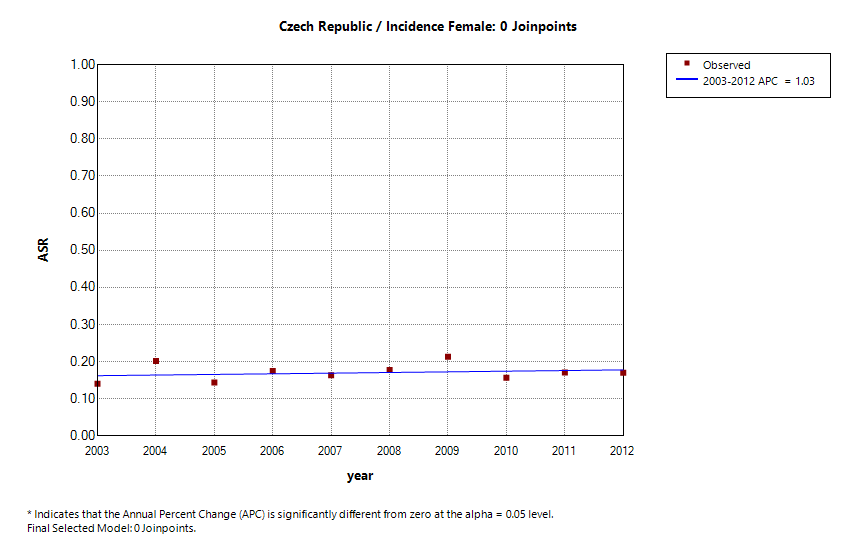 |
| 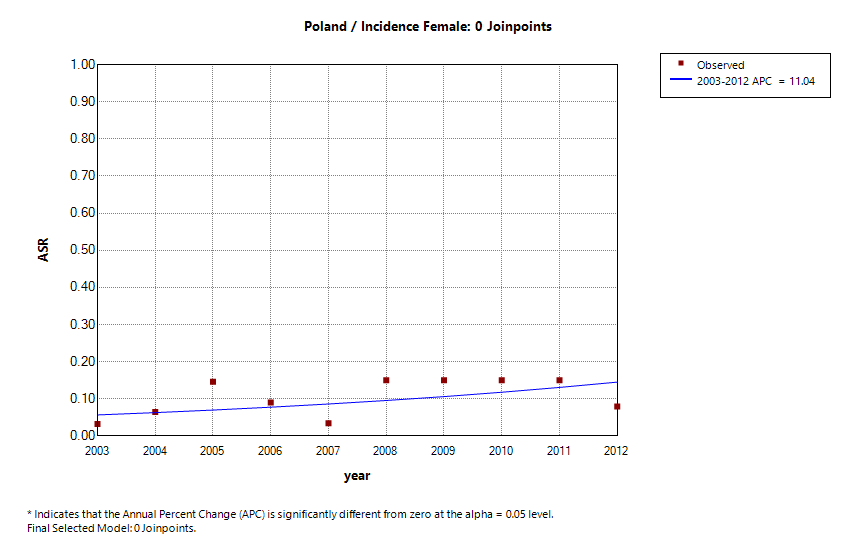 |  |

| **Africa** | |
| --- | --- |
| 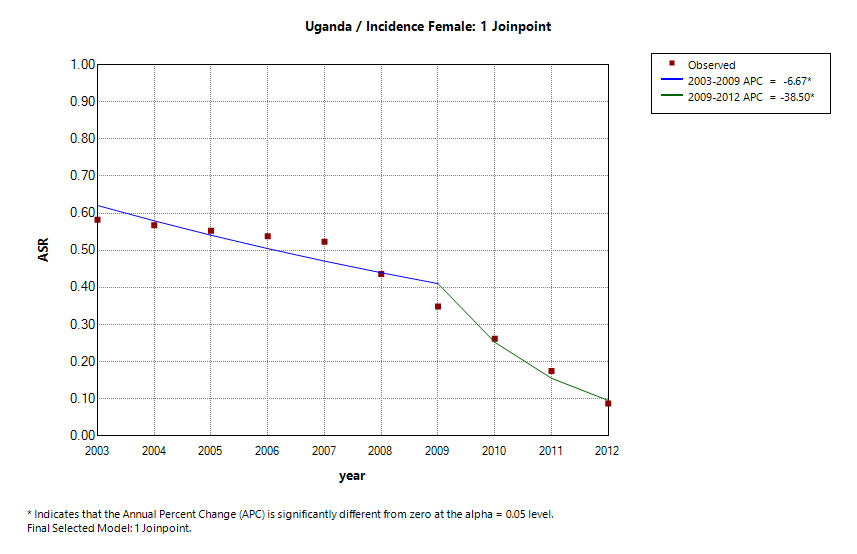 |  |

#### Ureter - Both

| **Asia** | |
| --- | --- |
| 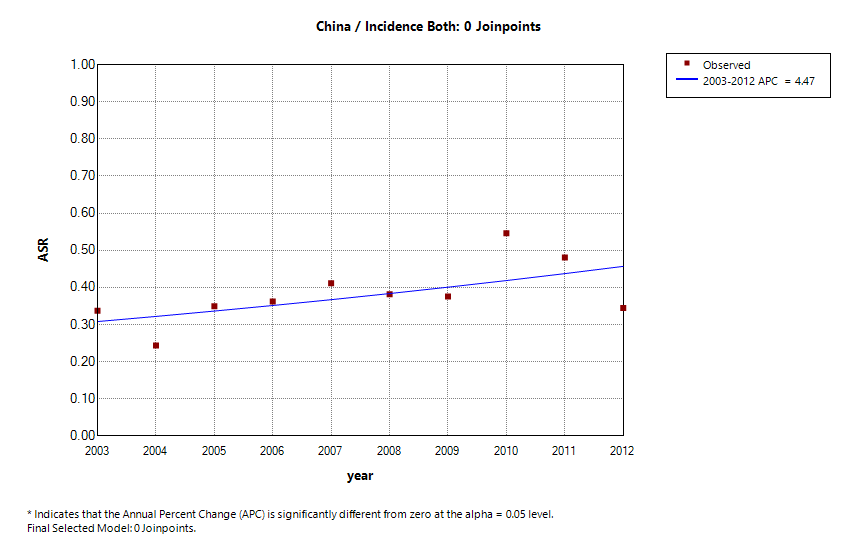 | 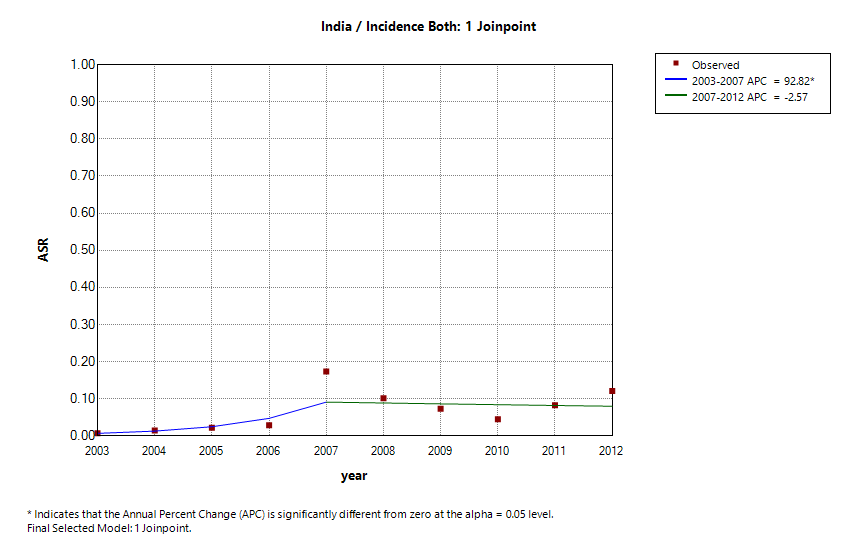 |
| 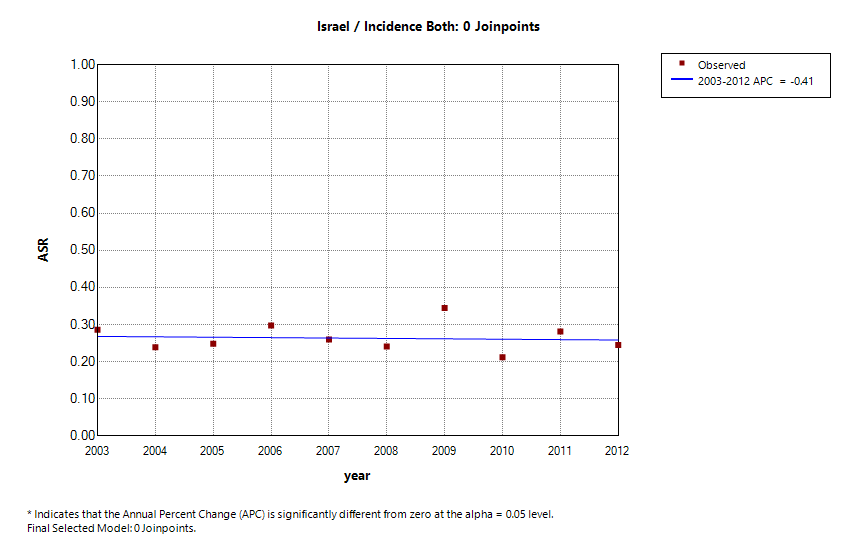 | 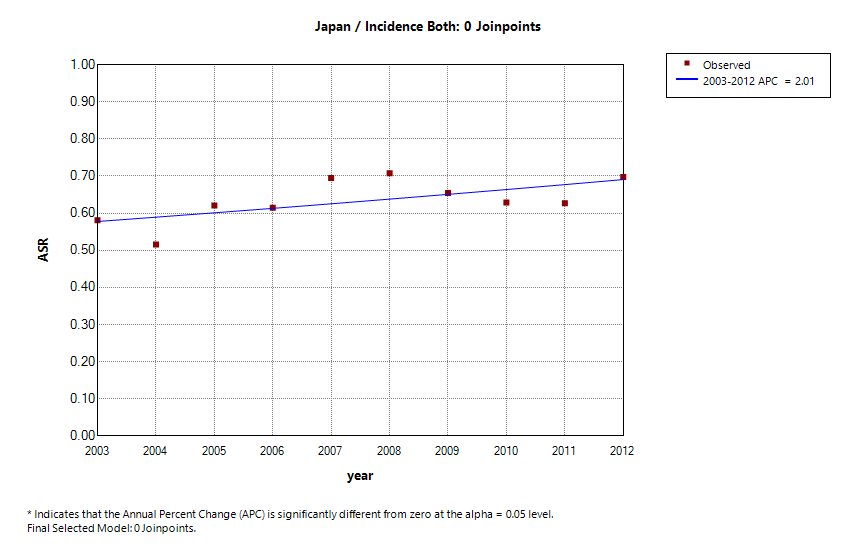 |
| 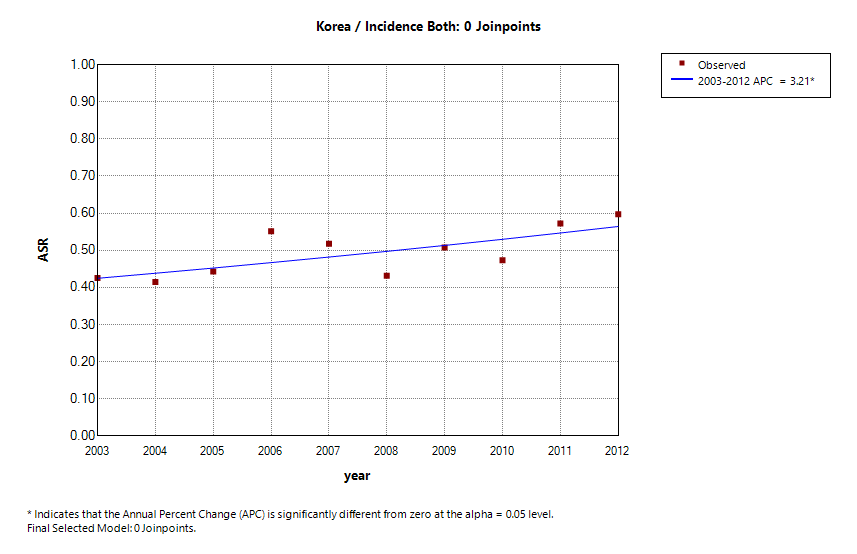 | 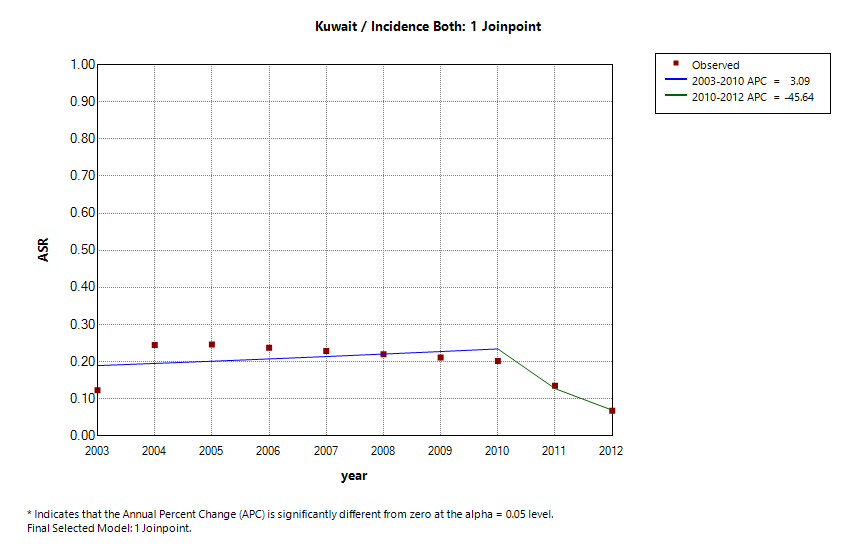 |

| 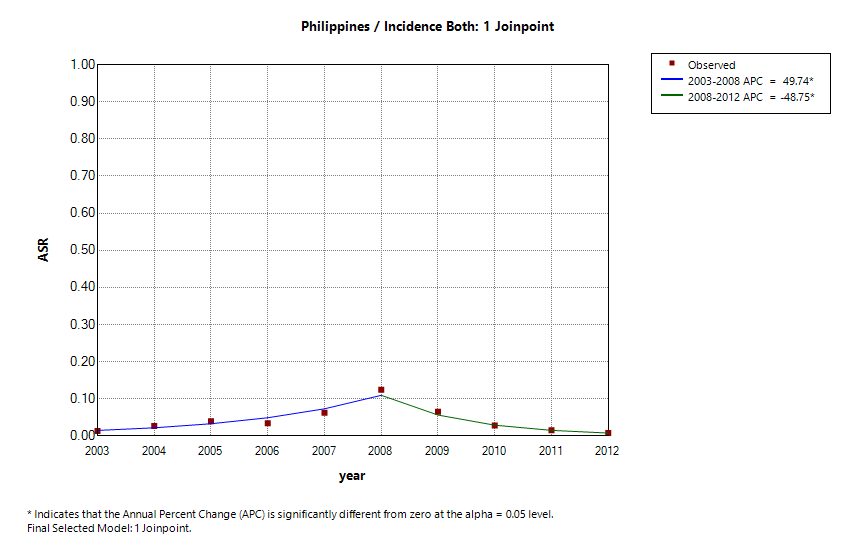 | 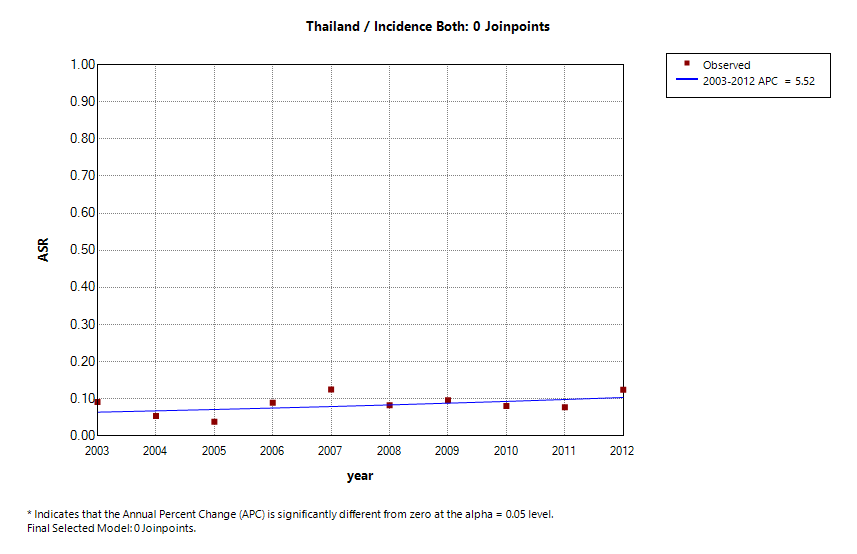 |
| --- | --- |
| 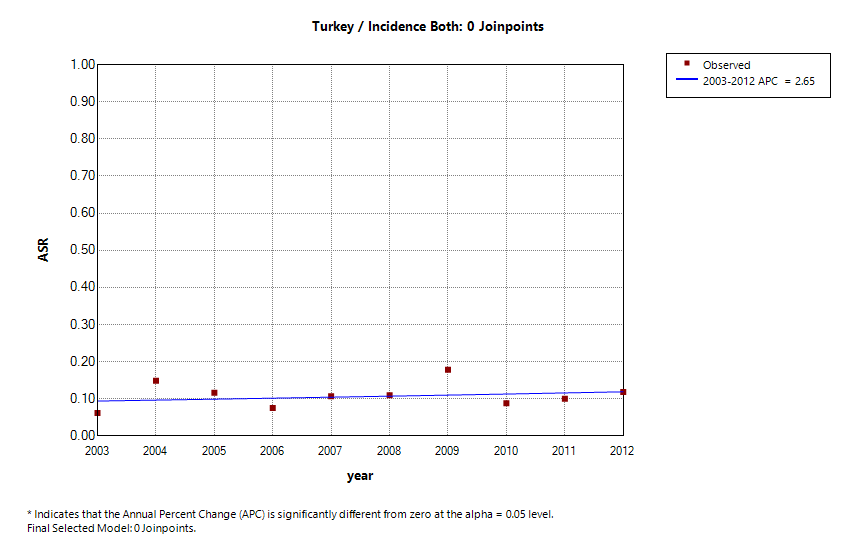 |  |
| **Oceania** | |
| 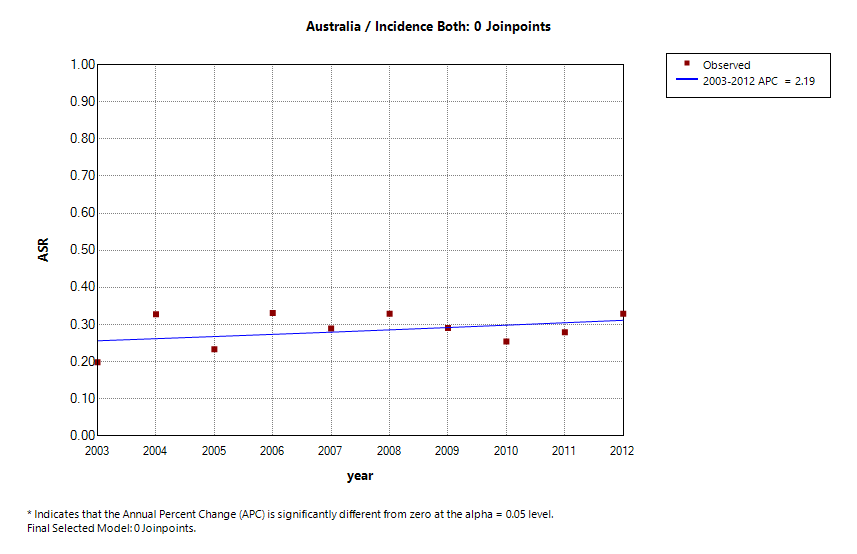 | 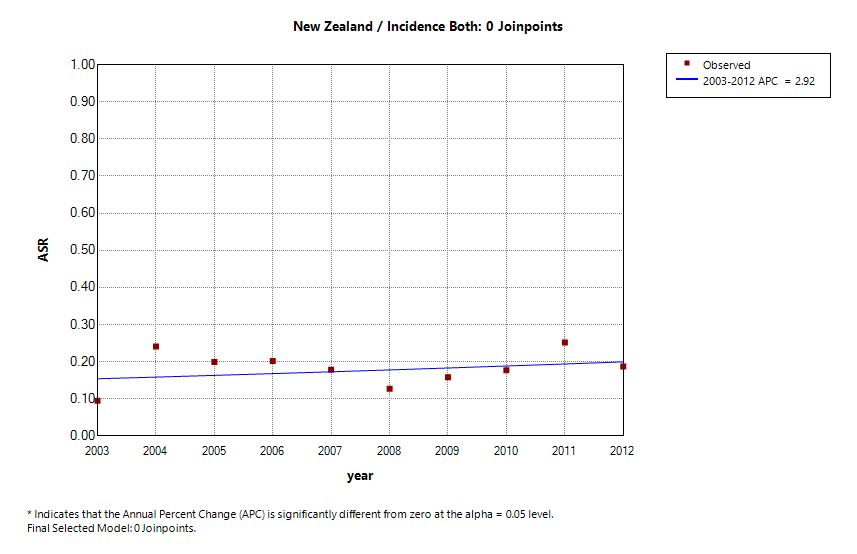 |

| **Northern America** | |
| --- | --- |
| 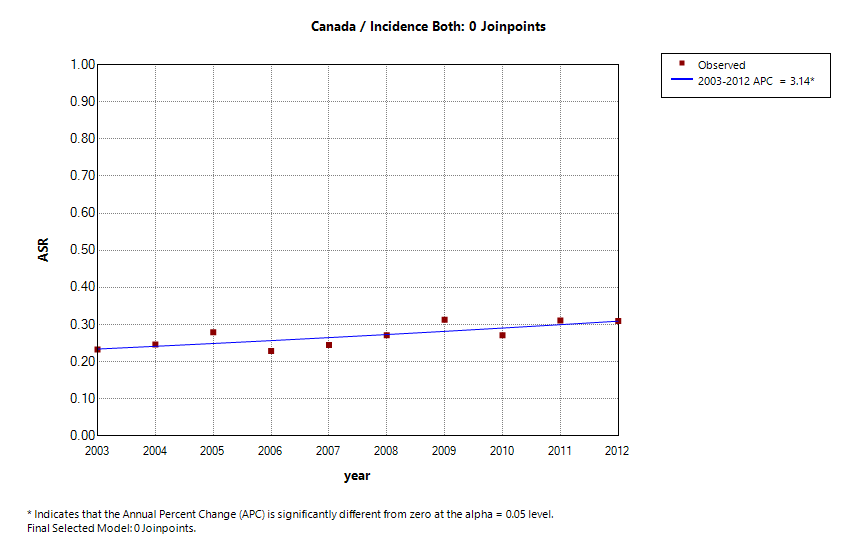 | 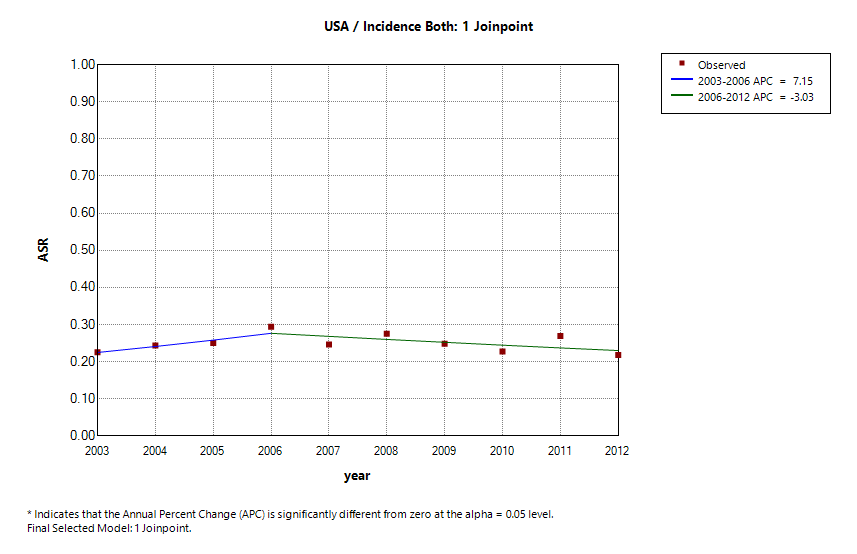 |
| **Southern America** | |
| 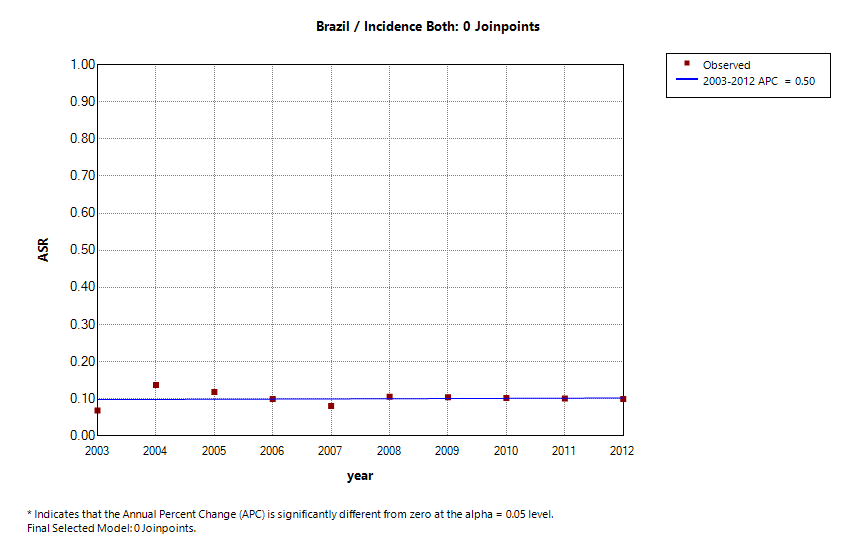 | 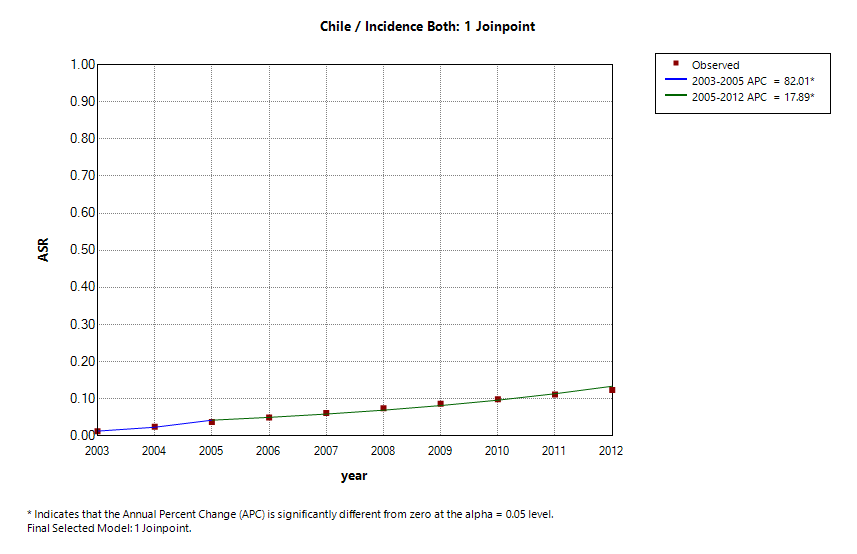 |
| 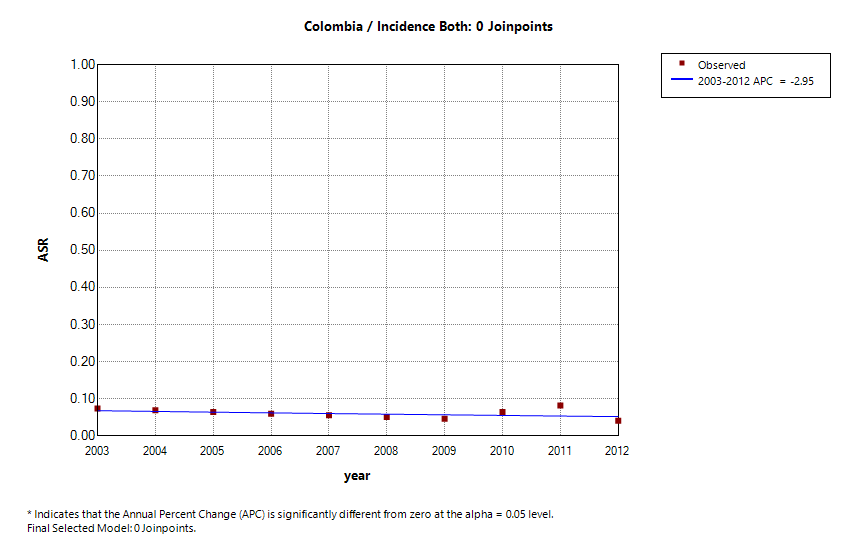 | 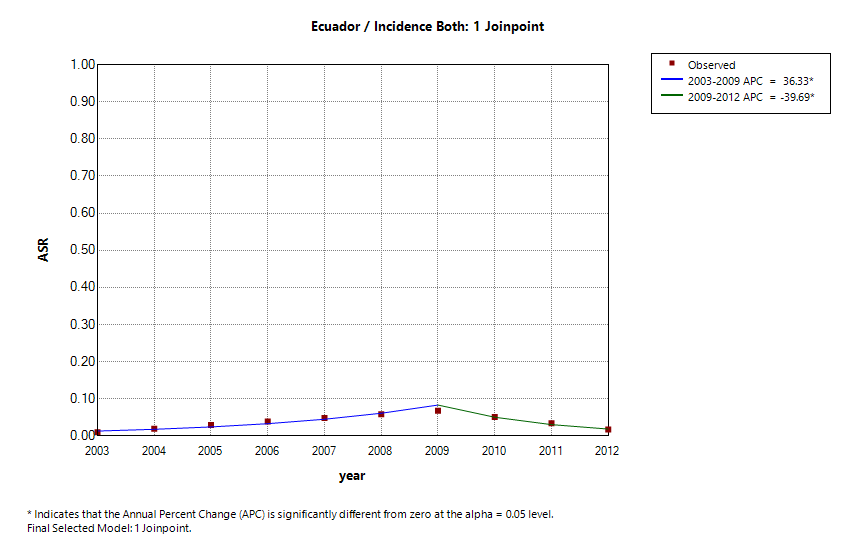 |

| 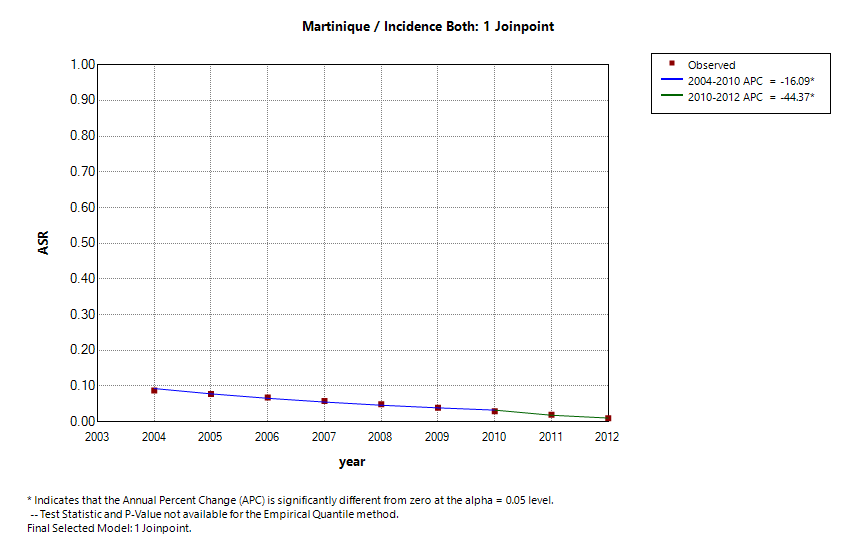  * 2003 data excluded as outliner |  |
| --- | --- |
| **Northern Europe** | |
| 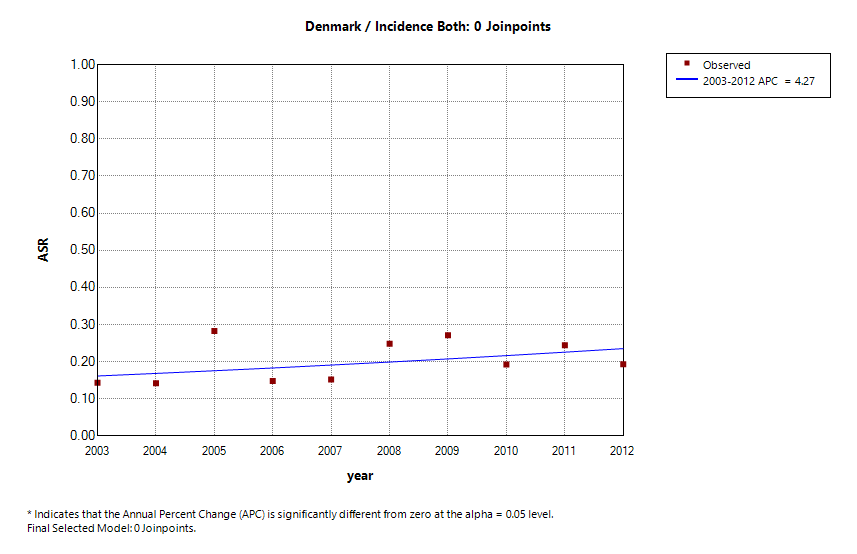 | 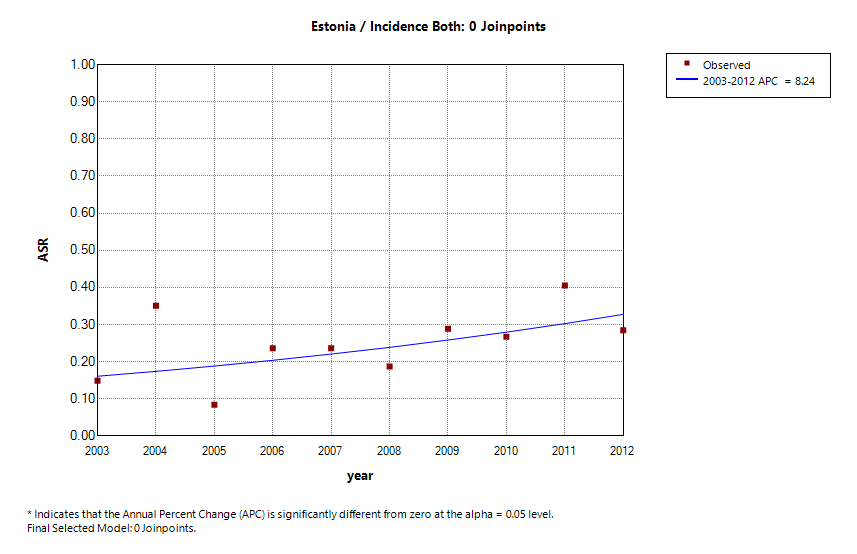 |
| 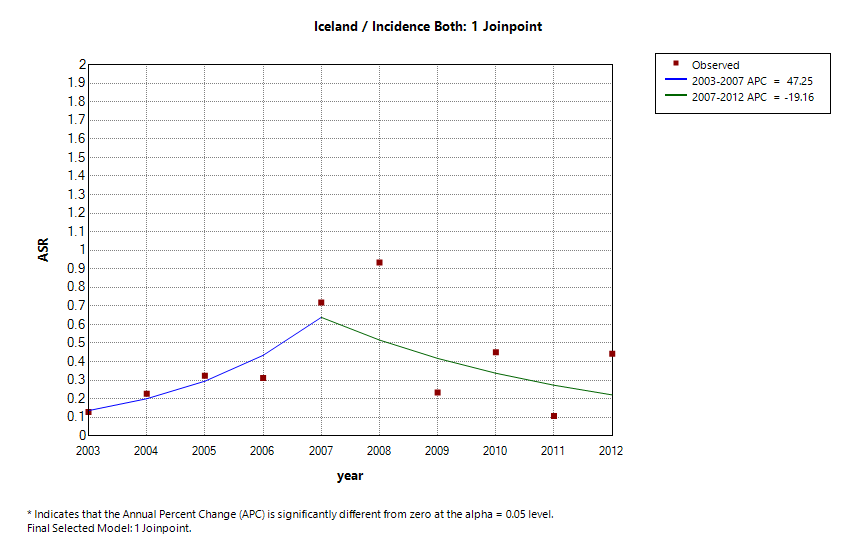 | 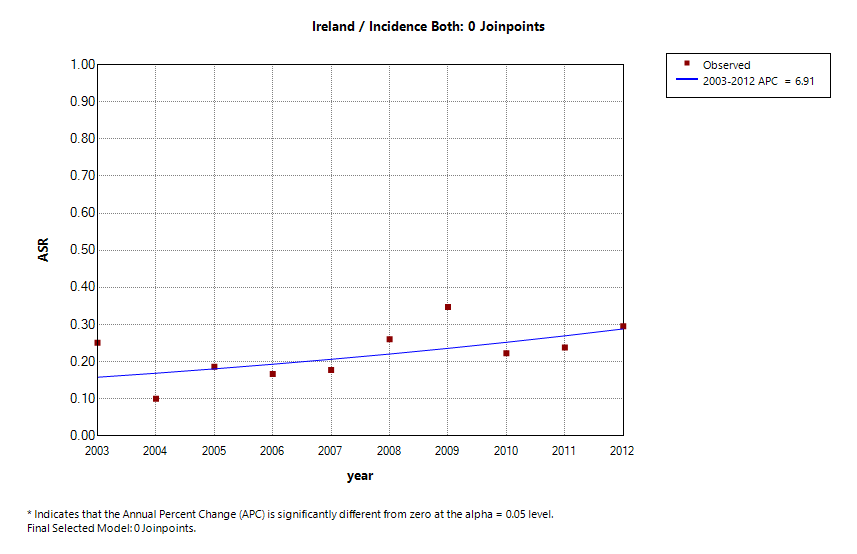 |

| 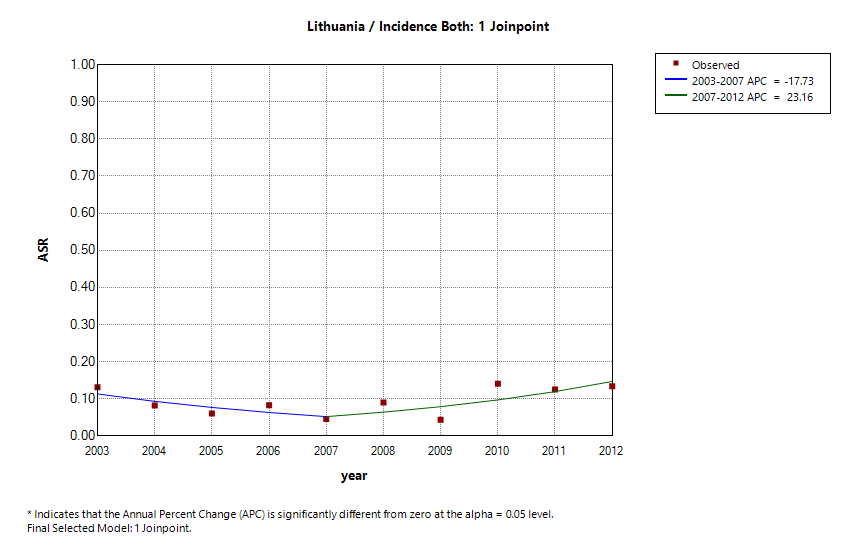 |  |
| --- | --- |
|  |  |
| **Western Europe** | |
|  |  |

|  |  |
| --- | --- |
|  |  |
| **Southern Europe** | |
|  |  |

|  |  |
| --- | --- |
|  |  |
| **Eastern Europe** | |
|  |  |

|  |  |
| --- | --- |
| **Africa** | |
|  |  |

1. Ureter - Young

| **Asia** | |
| --- | --- |
|  |  |
|  |  |
|  |  |

|  |  |
| --- | --- |
| **Oceania** | |
|  |  |
| **Northern America** | |
|  |  |

| **Northern Europe** | |
| --- | --- |
|  |  |
|  |  |
|  |  |

| **Western Europe** | |
| --- | --- |
|  |  |
|  |  |
|  |  |

| **Southern Europe** | |
| --- | --- |
|  |  |
|  |  |
|  |  |

| **Eastern Europe** | |
| --- | --- |
|  |  |
|  |  |

1. Ureter - Old

| **Asia** | |
| --- | --- |
|  |  |
|  |  |
|  | * 2003 data excluded as outliner |

|  |  |
| --- | --- |
|  |  |
| **Oceania** | |
|  |  |

| **Northern America** | |
| --- | --- |
|  |  |
| **Southern America** | |
| * 2003 data excluded as outliner |  |
|  |  |

| **Northern Europe** | |
| --- | --- |
|  |  |
|  |  |
|  |  |

|  |  |
| --- | --- |
| **Western Europe** | |
|  |  |
|  |  |

|  |  |
| --- | --- |
| **Southern Europe** | |
|  |  |
|  | * 2003 data excluded as outliner |

|  |  |
| --- | --- |
| **Eastern Europe** | |
|  |  |
|  |  |

| **Africa** | |
| --- | --- |
|  |  |
